# Unexpected Periodicity in Cationic Group 5 Initiators for the Ring-Opening Polymerization of Lactones

**DOI:** 10.1021/acs.inorgchem.3c03854

**Published:** 2023-12-20

**Authors:** Antoine Buchard, Matthew G. Davidson, Gerrit Gobius du Sart, Matthew D. Jones, Gabriele Kociok-Köhn, Strachan N. McCormick, Paul McKeown

**Affiliations:** † Institute for Sustainability, University of Bath, Bath BA2 7AY, United Kingdom; ‡ Department of Chemistry, University of Bath, Bath BA2 7AY, United Kingdom; § TotalEnergies Corbion, Stadhuisplein 70, Gorinchem 4203 NS, The Netherlands; ∥ Material and Chemical Characterization and Analysis Facility (MC^2^), University of Bath, Bath BA2 7AY, United Kingdom

## Abstract

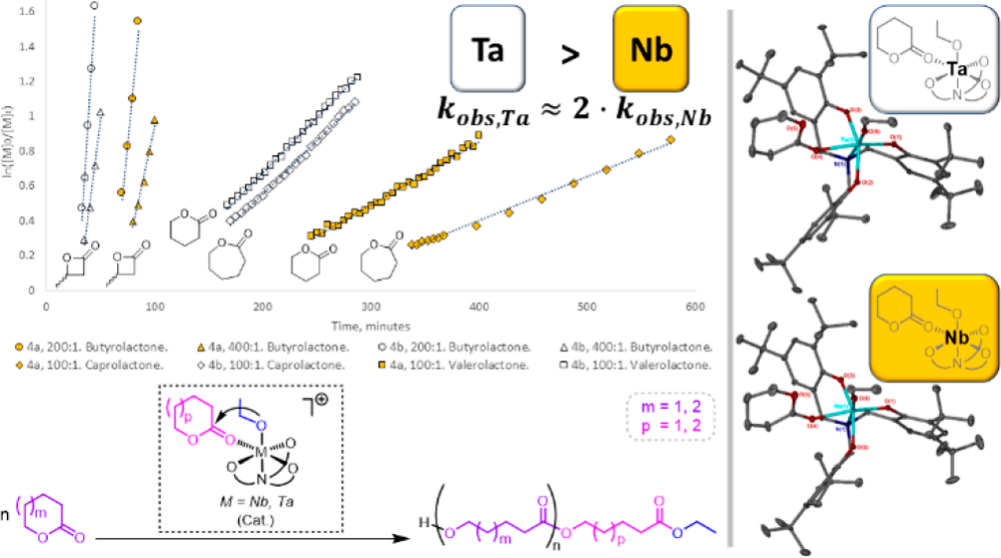

ε-Caprolactone (ε-CL) adducts of cationic, amine tris(phenolate)-supported niobium(V) and tantalum(V) ethoxides initiate the ring-opening polymerization of lactones. The Ta(V) species prepared and applied catalytically herein exhibits higher activity in the ring-opening polymerization (ROP) of ε-caprolactone than the previously reported, isostructural Nb(V) complex, contradicting literature comparisons of Nb(V)- and Ta(V)-based protocols. Both systems also initiate the ROP of δ-valerolactone and *rac*-β-butyrolactone, kinetic studies confirming retention of higher activity by the Ta congener. Polymerizations of *rac*-β-butyrolactone and δ-valerolactone were previously unrealized under Group V- or Ta-mediated conditions, respectively, although the former has afforded only low molecular weight, cyclic poly-3-hydroxybutyrate. Cationic ethoxo–Nb(V) and −Ta(V) δ-valerolactone adducts are also reported, demonstrating the facility of δ-valerolactone as a ligand and the generality of the synthetic method. Both δ-valerolactone-bearing complexes initiate the ROP of ε-caprolactone, δ-valerolactone, and *rac*-β-butyrolactone. Accordingly, we have elucidated trends in reactivity and investigated the initiation mechanism for such systems, the insertion event being predicated upon intramolecular nucleophilic attack on the coordinated lactone by the adjacent alkoxide moiety. This mechanism enables quantitative, stoichiometric installation of a single monomer residue distinct from the bulk of the polymer chain, and permits modification of polymer properties via both manipulation of the molecular architecture and tuning of the polymerization kinetics, and thus dispersity, through hitherto inaccessible independent control of the initiation event.

## Abstract 2

Niobium(V) and tantalum(V) alkoxide complexes bearing coordinated ε-caprolactone and δ-valerolactone molecules were applied to the catalytic ring-opening polymerization of ε-caprolactone, δ-valerolactone, and *rac*-β-butyrolactone. Each complex is stable at ambient temperature, initiation proceeding via ring opening of the coordinated monomer on heating. Contrary to literature precedent, all Ta-based systems exhibited markedly higher activity than their Nb analogues. Initiation of lactone polymerization by stoichiometrically ring opening a different (metal-coordinated) lactone provides opportunities for manipulation of the polymer architecture and properties.

## Introduction

The replacement of environmentally persistent, petrochemical-derived thermoplastics in low-cost and single-use applications is reliant upon provision of inexpensive, biodegradable alternatives with comparable thermomechanical properties. Biodegradable aliphatic polyesters, several of the most industrially relevant of which are typically prepared via the ring-opening polymerization (ROP) of cyclic esters, have received significant attention regarding their potential role in replacing traditional plastics. ROP processes of lactides, LA, and ε-caprolactone, ε-CL, are already commercialized, yielding the homopolymers poly(lactic acid), PLA, and poly(ε-caprolactone), PCL, as well as associated copolymers, such as those formed upon copolymerization with glycolide.^1−4^ These materials have been the subject of widespread research attention and commercial deployment for biomedical and rapid prototyping applications with packaging use a burgeoning market.^3,5,14−17,6−13^ Another aliphatic polyester, poly(3-hydroxybutyrate), P3HB, is produced by several species of bacteria as an energy storage medium. Bacterial P3HB is entirely isotactic and is thus a highly crystalline solid, with properties comparable to those of isotactic polypropylene.^18^ However, commercialization of this material has not been realized due to high production costs and limited scalability. Chemical syntheses of P3HB via the stereoselective metal-catalyzed ROP of racemic β-butyrolactone, *rac*-β-butyrolactone, β-BL,^19−23^ and, with notable success, of the corresponding cyclic diolide^18^ have therefore received interest as alternative strategies.

The living ring-opening polymerization of lactones is typically initiated by a metal complex and proceeds via either an activated monomer^24,25^ or a coordination–insertion mechanism.^3,16,21,26−30^ While many organocatalytic systems have also been reported, these are not the focus of the current work and will not be discussed in detail.^27,31−35^ In the former mechanistic scenario, the monomer carbonyl group is activated by the Lewis acidic metal center, facilitating nucleophilic attack by an exogenous alcohol nucleophile, a role occupied during propagation by the growing polymer chain. The molecular weight of the polymer product is, accordingly, determined by the molar ratio of the monomer and nucleophile.^21,24,25^ The coordination–insertion pathway relies upon introduction or in situ generation of a metal alkoxide precatalyst. In this case, coordination of the monomer to the metal center precedes intramolecular nucleophilic attack at the ester carbonyl group by the alkoxide moiety, which is followed by ring opening. With the exception of our recently reported stable alkoxo–niobium(V) ε-CL adduct (see below), the intermediate formed on coordination of the monomer, prior to the insertion event, is not typically observable.^36^ After insertion and ring opening occur, the growing chain remains coordinated to the metal center, fulfilling the role of the alkoxide in subsequent propagation events. An exogenous nucleophile is not required, and the chain length is determined by the precatalyst concentration, relative to that of the monomer. Some coordination–insertion regimes are immortal in character, meaning the polymer molecular weight can be reduced in a controlled manner by inclusion of an exogenous alcohol nucleophile (chain transfer agent).^3,16,26−28^

Examples in the literature of protocols for the ring-opening polymerization of cyclic esters catalyzed by complexes of the Group 5 metals are scarce, and to our knowledge, these elements have never been applied to the ROP of β-BL.^37−42^ There is only one example of the niobium- or tantalum-catalyzed ROP of δ-valerolactone, δ-VL, that concerning an unusual trihydroniobocene system reported by Otero and co-workers.^41^ Moreover, the authors are aware of only five reports describing application of structurally analogous Nb and Ta complexes to the polymerization of cyclic esters^37−40,43^ and from only three of those can any comparison of relative activity be drawn.^37−39^

Pampaloni and co-workers prepared Nb and Ta tetrakis(ethoxide) complexes supported by an α-amino acid-derived (l-phenylalanine) ancillary ligand and applied them to the solvent-free ROP of l-LA and *rac*-LA.^38^ The catalytic activity for all of those systems was marginally higher than that of the respective pentakis(ethoxide) precursors, with Nb exhibiting slightly higher activity than Ta (0.33 mol % of the Nb and Ta α-amino acidato complexes afforded 95% and 88% conversion, respectively, in the ROP of l-LA and 86% and 77% conversion, respectively, for the ROP of *rac*-LA after 15 h at 135 °C).

Chakraborty and co-workers, similarly, observed slightly higher activity for Nb than Ta in the ROP of *rac*-LA and l-LA initiated by zwitterionic aminophenolate complexes.^39^ Therein, ROP proceeded via a coordination–insertion mechanism involving the metal–phenolate bond, even when ethoxide ligands were also present at the metal center. Immortal polymerization kinetics were accessible in the presence of exogenous benzyl alcohol, and polymer dispersity remained low (*Đ*_M_ ≤ 1.20). The same group’s structurally related iminophenolate systems were more active than the aminophenolates and slightly more stereoselective in the ROP of *rac*-LA (*P*_*r*_ = 0.70–0.72 compared to *P*_*r*_ = 0.66–0.69). Those systems were also active for the ROP of ε-CL, providing PCL of high molecular weight and low dispersity (*Đ*_M_ ≤ 1.08). In the ROP of lactides and ε-CL, there was, again, a subtle increase in activity when Nb was used, compared to Ta.^37^

Chakraborty and co-workers have also described the controlled ROP of glycidol at the epoxide moiety, mediated variously by Nb and Ta benzotriazole and benzoxazole phenoxide complexes, producing a hyperbranched polyether. In that work, the Nb species exhibited higher activity than analogous Ta systems, attributed to the lighter metal’s greater electropositivity.^44^

Previous work in our group has been concerned with the catalytic application of highly stereoselective, amine tris(phenolate)-supported zirconium and hafnium alkoxide complexes to the ROP of *rac*-LA. Both at ambient temperature in toluene and under solvent-free conditions at 130 °C, the Zr system was significantly more active than its Hf counterpart (e.g., 50% versus 30% conversion after 48 h at ambient temperature, [*rac*-LA]/[Cat.] = 300). However, the corresponding titanium complex was much less active than either the Zr or the Hf species.^45^ A similar trend was reported by Kol and co-workers for Ti and Zr mono(alkoxide) and bis(alkoxide) complexes bearing amine tris(phenolate) and amine bis(phenolate) ancillary ligands, respectively, in the ROP of l-LA.^46^ We have also previously applied Zr and Hf alkoxides supported by various unsymmetrical amine tris(phenolate) scaffolds to the ROP of β-BL in toluene at 80 °C. While those systems exhibited moderate syndioselectivity, the Zr complexes were consistently much more active than their Hf equivalents.^19^ Most recently, Kol and co-workers have reported the application of Group 4 metal alkoxide complexes supported by a *C*_3_-symmetric amine tris(phenolate) ligand, bearing sterically demanding mesityl substituents, to the highly efficient solvent-free ROP of unpurified l-LA with the activity of the Zr system again apparently surpassing that of its Hf analogue.^47^

We have previously reported the facile isolation as the corresponding hexafluoroantimonate salt, **4a**, of a cationic amine tris(phenolate)-supported Nb(V) alkoxide complex bearing a coordinated molecule of ε-CL. This was the first reported example of a stable cyclic ester adduct of a metal alkoxide complex, and when heated to 80 °C in a solution of ε-CL in toluene, it initiated the controlled ROP of that monomer. Moreover, we demonstrated that the alkoxide moiety was the active initiating group and that ROP proceeded via a coordination–insertion mechanism.^36^ Encouraged by the results of that work, we report here the preparation of the analogous Ta(V) species, **4b**, and its utility in application to the catalytic ROP of ε-CL. Both Nb and Ta systems have also been applied to the ROP of δ-VL and β-BL. In marked contrast to trends reported for other mutually comparable Nb and Ta systems^37−39^ and for our group’s and for Kol and co-workers’ various amine tris(phenolate)-supported Zr and Hf isopropoxide ROP initiators,^19,45,47^ the catalytic activity of the Ta species was consistently much higher than that of the lighter Nb congener. Furthermore, we report the synthesis and catalytic application of structurally analogous δ-VL adducts of cationic amine tris(phenolate)-supported Nb and Ta alkoxides.

## Results and Discussion

**Scheme 1:**
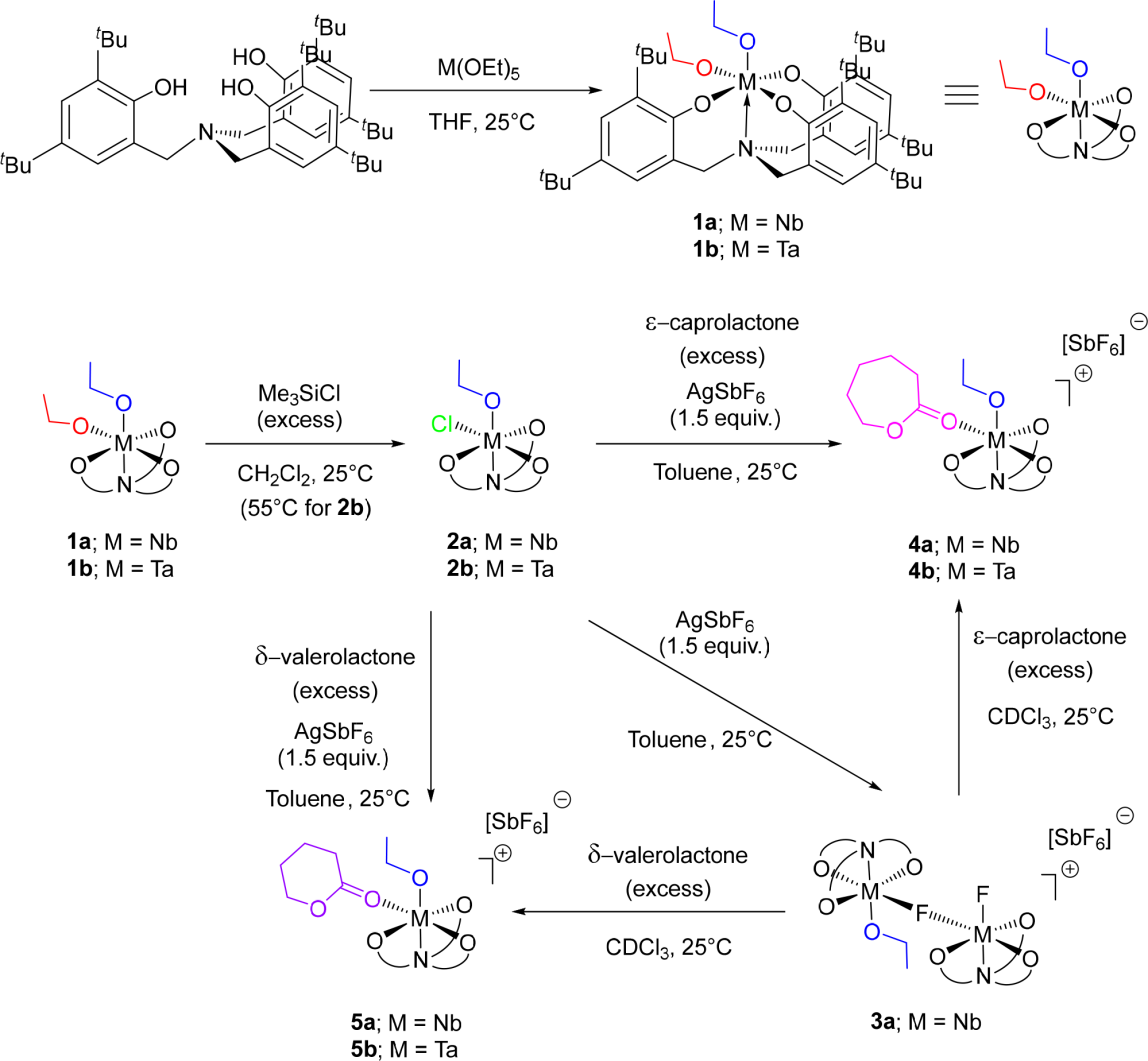
Synthesis of Nb and Ta Complexes **2b**, **4b**, **5a**, and **5b** from the Current Work

**Figure 1:**
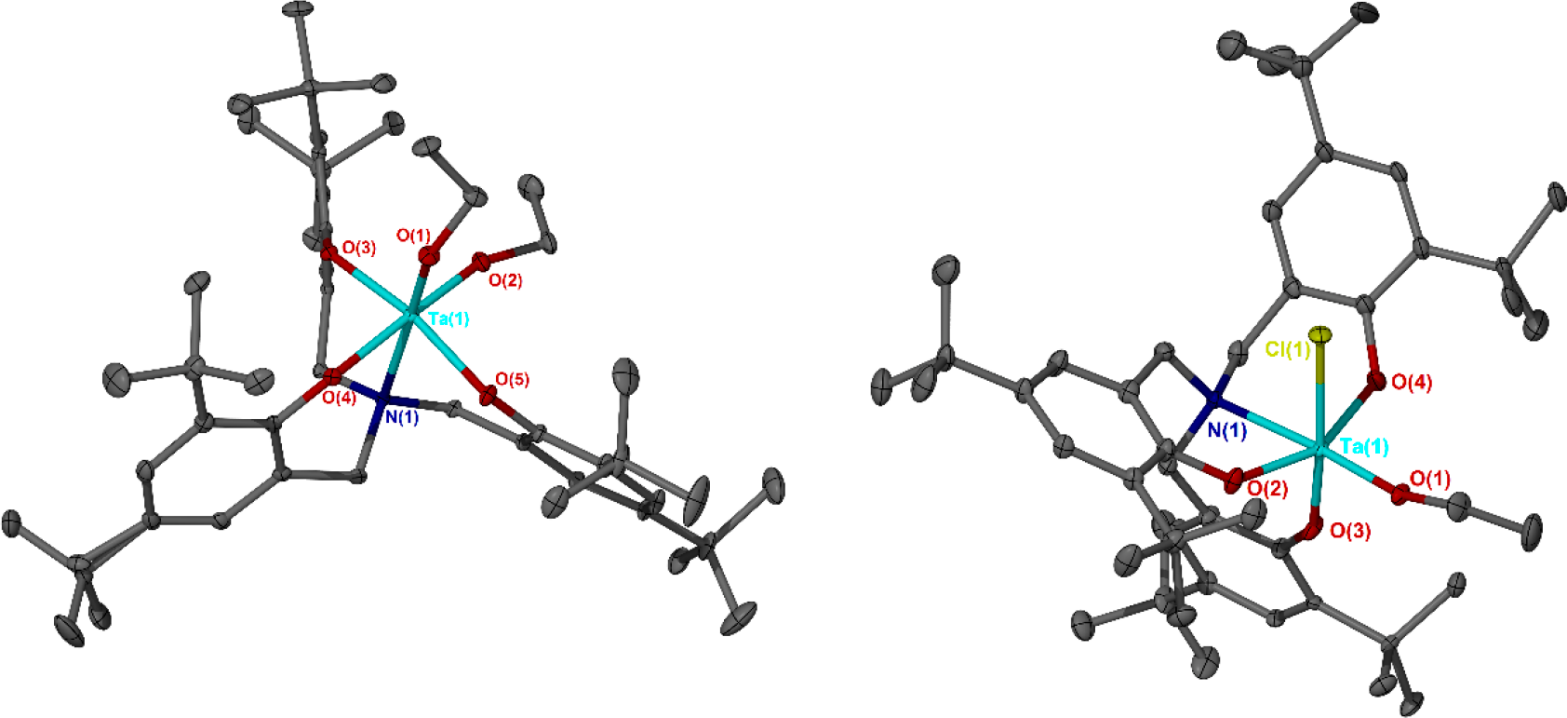
Solid-state structures of neutral Ta(V) complexes **1b** and **2b**. Ellipsoids shown at 30% probability level. Hydrogen atoms and lattice solvent have been omitted for clarity. Selected bond lengths (Angstroms) and angles (degrees): **1b** Ta(1)–O(1) 1.8741(18), Ta(1)–O(2) 1.8880(19), Ta(1)–N(1) 2.357(2), O(1)–Ta(1)–O(2) 97.82(8), O(1)–Ta(1)–N(1) 172.76(8), O(2)–Ta(1)–N(1) 89.01(7), O(1)–Ta(1)–O(4) 92.48(8), O(1)–Ta(1)–O(3) 100.99(8), O(1)–Ta(1)–O(5) 99.93(8), O(2)–Ta(1)–O(3) 86.99(8), O(2)–Ta(1)–O(5) 89.21(8); **2b** Ta(1)–O(1) 1.846(4), Ta(1)–Cl(1) 2.415(4), Ta(1)–N(1) 2.371(5), O(1)–Ta(1)–Cl(1) 93.49(16), N(1)–Ta(1)–O(1) 175.56(19), N(1)–Ta(1)–Cl(1) 90.64(15), O(1)–Ta(1)–O(3) 94.51(18), O(1)–Ta(1)–O(2) 101.0(2), O(1)–Ta(1)–O(4) 101.1(2), O(2)–Ta(1)–Cl(1) 85.39(17), O(4)–Ta(1)–Cl(1) 82.34(17).

Reaction of tantalum(V) ethoxide and the pro-ligand tris(2-hydroxy-3,5-di-*tert*-butylbenzyl)amine, H_3_L^tBu^, in THF yielded the bis(ethoxide) complex [L^tBu^Ta(OEt)_2_], **1b** (Scheme 1, Figure 1), and crystals suitable for X-ray diffraction were isolated from the mother liquor after volume reduction in vacuo (see Supporting Information). **1b** has previously been prepared by Kol and co-workers along with the corresponding bis(dimethylamido) system. However, in that work, a solid-state structure was not reported for the bis(ethoxide) species.^48^ In contrast to the *C*_1_-symmetric conformation adopted by the phenolate groups of Kol and co-workers’ bis(dimethylamido) complex, we observe that in the solid state, in structural analogy to our previously reported Nb complex [L^tBu^Nb(OEt)_2_], **1a**, the ligand scaffold of pseudo-octahedral complex **1b** exhibits pseudo-*C*_3_ symmetry about the tantalum–nitrogen axis.^36^ The difference in the symmetry adopted by the ancillary ligands of **1b** and of Kol’s bis(dimethylamido) system, respectively, is attributed to the greater steric demands of the dimethylamido ligand relative to the ethoxide moiety.^49^ Like **1a**, treatment of **1b** with excess TMSCl in the current work afforded the species [L^tBu^Nb(OEt)Cl], **2b**, monochlorinated by substitution of the ethoxide moiety cis to the bridgehead nitrogen of the ancillary ligand. Unexpectedly, however, quantitative conversion of **1b** required the reaction mixture to be heated to 55 °C for 3 days, whereas chlorination of **1a** in our previous work was facile at ambient temperature.^36^

**Figure 2:**
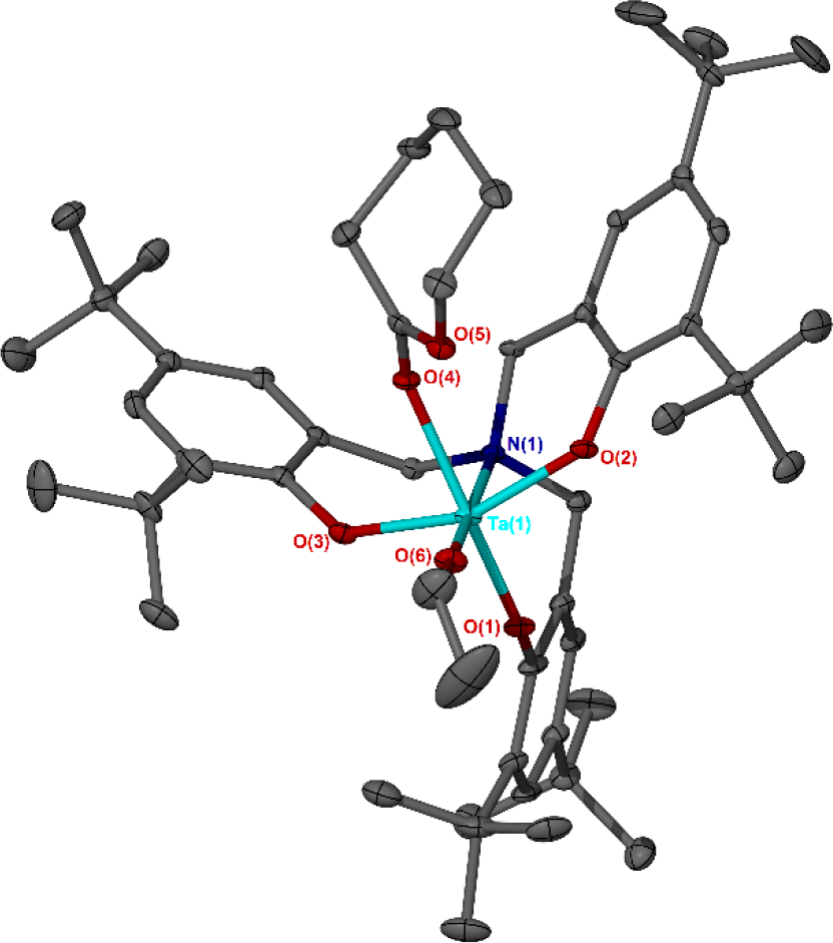
Solid-state structure of the cationic fragment of Ta(V)−ε-caprolactone adduct **4b**. Ellipsoids shown at 30% probability level. Hydrogen atoms, lattice solvent, and SbF_6_^–^ anion have been omitted for clarity. Selected bond lengths (Angstroms) and angles (degrees): Ta(1)–N(1) 2.327(4), Ta(1)–O(6) 1.835(4), Ta(1)–O(4) 2.147(4), O(4)–C(46) 1.237(6), O(5)–C(46) 1.287(6), O(6)–Ta(1)–N(1) 177.18(16), O(6)–Ta(1)–O(4) 95.72(16), O(1)–Ta(1)–O(4) 165.34(15), O(6)–Ta(1)–O(1) 98.49(17), O(2)–Ta(1)–O(3) 154.62(16), O(2)–Ta(1)–O(4) 83.13(15), O(3)–Ta(1)–O(4) 78.07(15), O(6)–Ta(1)–O(2) 99.30(17), O(6)–Ta(1)–O(3) 99.39(17), O(4)–Ta(1)–N(1) 82.31(15), C(46)–O(4)–Ta(1) 136.3(4).

While we have previously reported that treatment of Nb complex **2a** with 1.5 equiv of AgSbF_6_ afforded the hexafluoroantimonate salt [{L^tBu^Nb(OEt)}-μ_2_F-{L^tBu^NbF}]^+^[SbF_6_]^−^ of monocationic, bimetallic species **3a**,^36^ in the current work we were consistently unable to prepare the corresponding Ta complex. However, reaction of **2b** with AgSbF_6_ in the presence of excess ε-CL in toluene readily yielded from the filtered reaction mixture, as a colorless crystalline solid, the hexafluoroantimonate salt [L^tBu^Ta(OEt)(ε-CL)]^+^[SbF_6_]^−^, **4b**, of a pseudo-octahedral cationic ε-CL adduct of a monoalkoxo Ta amine tris(phenolate) complex (46% yield with respect to **2b**). The cationic fragment of **4b** was confirmed to be structurally analogous to that of our previously reported Nb system, **4a** (Scheme 1, Figure 2).^36^ Moreover, as with the preparation of **4a**, no ring opening of the ε-CL present was observed to occur during the synthesis of **4b**. In further similarity to **4a** and consistent with complexes **1a**, **1b**, **2a**, and **2b** of ligand (L^tBu^)^3–^, the ligand system of the cation of Ta species **4b** exhibited pseudo-*C*_3_ symmetry about the metal–nitrogen axis. Analogues of **1a**, **1b**, **2a**, and **2b** have also been prepared by us and others using the less bulky pro-ligand tris(2-hydroxy-3,5-di-*tert*-butylbenzyl)amine, H_3_L^Me^. However, in all of those systems, the ligand framework was *C*_1_ symmetric about the metal–nitrogen axis, and attempts to isolate the corresponding ε-CL adducts were consistently unsuccessful.^36,49,50^ These observations can presumably be attributed to the limited kinetic stabilization afforded by the lower steric profile of the methyl-substituted ancillary ligand.

When the bond lengths and bond angles about the Nb center and of the ester moiety of the coordinated ε-CL molecule were considered, there were no statistically significant differences between the solid-state structures of **4a** and **4b**. The retention of the solid-state structures of all Ta complexes in solution was confirmed by NMR spectroscopy, although acquisition of a well-resolved ^1^H NMR spectrum of **4b** was possible only on cooling to 223 K.

Despite extensive washing and repeated precipitation steps, **4b** could not be isolated without a small amount of residual uncoordinated ε-CL remaining present (0.15 equiv, quantified via ^1^H NMR spectroscopy). Both **4a** and **4b** are also thermally unstable, the coordinated monomer carbonyl group in both cases undergoing intramolecular nucleophilic attack by the adjacent alkoxide moiety on gentle heating (≤65 °C). This process is consistent with the insertion event of the coordination–insertion ROP mechanism, albeit without subsequent quantitative occupation of the vacant coordination site by a further equivalent of the monomer, which may be anticipated to stabilize the resulting species. Furthermore, the coordinated ε-CL molecule of **4b** appears to be lost on prolonged exposure to vacuum (see Supporting Information). Accordingly, vacuum drying of **4a** and **4b** was only carried out briefly, precluding complete removal of residual lattice toluene from the solid products. On heating to 65 °C in chloroform-*d*, intramolecular nucleophilic attack occurred more rapidly for **4b** than **4a**, indicating that the activation barrier to initiation of ROP is lower for the Ta complex than that for the structurally indistinguishable Nb system (see Supporting Information). ε-CL and solvent impurities present in samples of **4a** and **4b** were quantified by ^1^H NMR spectroscopy prior to catalytic use and accounted for when calculating catalyst loadings.

Highly colored species are generally undesirable in polymerization catalysis due to the likelihood of catalyst residues remaining within the polymer product. The Ta complexes **1b** and **2b** and the hexafluoroantimonate salt **4b** were all noted to be colorless, unlike the corresponding Nb species **1a**, **2a**, and **4a**, all of which are an intense yellow color, characteristic of Nb(V) species. Moreover, in catalytic use, **4b** yielded PCL, P3HB, and poly(δ-valerolactone), PVL, that were already colorless prior to purification. Like **4a**, **4b** was inactive for the polymerization of lactide at 80 °C in toluene (see Supporting Information).

## Polymerization Studies

### Polymerization of ε-Caprolactone

**Figure 3:**
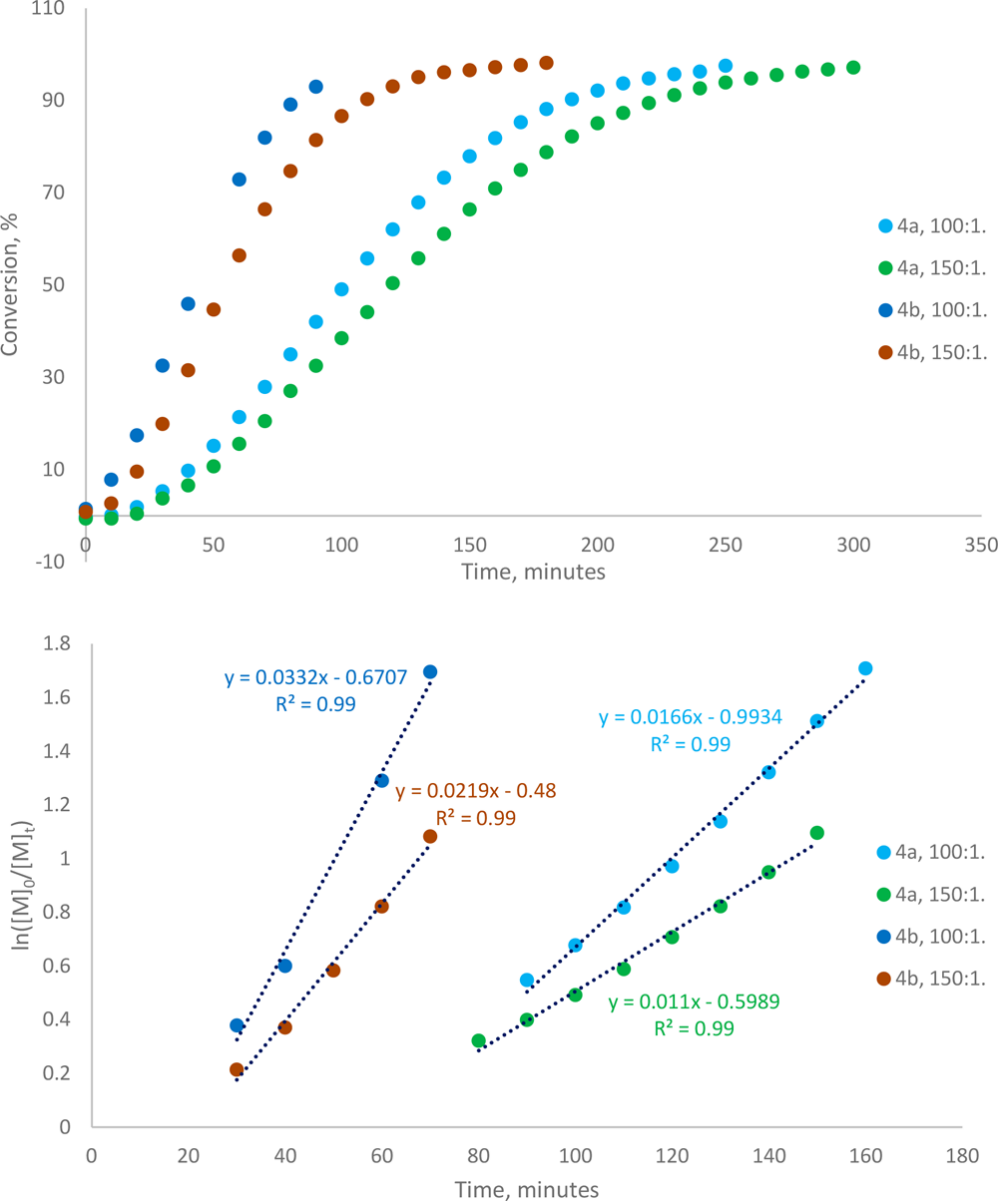
Reaction profiles and semilogarithmic plots for the ROP of ε-CL at 80 °C in toluene-*d*_8_ in the presence of initiators **4a** and **4b**. When [**4a**]:[ε-CL] = 100:1, *k*_obs_ = 0.017 min^–1^. When [**4a**]:[ε-CL] = 150:1, *k*_obs_ = 0.011 min^–1^. When [**4b**]:[ε-CL] = 100:1, *k*_obs_ = 0.033 min^–1^. When [**4b**]:[ε-CL] = 100:1, *k*_obs_ = 0.022 min^–1^.

When applied to the catalytic ring-opening polymerization of ε-CL in toluene-*d*_8_ at 80 °C, **4b** yielded a reaction profile similar to that of Nb species **4a**; the kinetics were pseudo-first order with respect to the monomer following a brief induction period (Table 1, Figure 3), attributed to homogenization of the reaction mixture. Both **4a** and **4b** are only sparingly soluble at ambient temperature under the conditions used for kinetic studies ([ε-CL] = 1.0 mol dm^–3^, unstirred in a J Young’s NMR tube), and increasing the catalyst loading above 1.0 mol % produced little increase in rate for either system, consistent with a solubility effect arising at higher concentrations.

**Table 1:** Polymerization Data for the ROP of ε-CL in the Presence of **4a** and **4b**

entry	catalyst	[monomer]/[Cat.]/[BnOH]	temp,°C	duration, min	conversion, %^f^	*M*_n_^Theo^,^g^ g mol^–1^	*M*_n_^GPC^,^h^ g mol^–1^	*Đ*_M_^h^	*k*_obs_,^i^ min^–1^	TOF,^j^ h^–1^
1^a^	**4b**	150/1/0	80	140	98	16 939	19 550	1.53		
2^a^	**4b**	200/1/0	80	190	97	22 303	23 600	1.61		
3^a^	**4b**	250/1/0	80	240	99	28 410	28 250	1.39		
4^b^	**4b**	500/1/0	80	450	99	56 660	41 850	1.44		
5^c^	**4b**	250/1/1.5	80	240	99	11 429	13 700	1.32		
6^c^	**4b**	250/1/4	80	240	99	5768	7950	1.24		
7^d^	**4b**	45/1/0	80	120	98	5194	9050	1.33		
8^d^	**4b**	67.5/1/0	80	120	98	7711	12 200	1.32		
9^d^	**4b**	90/1/0	80	120	99	10 330	14 650	1.32		
10^e^	**4a**	100/1/0	80	150	98	11 346	10 200	1.30	0.017	39.9
11^e^	**4a**	150/1/0	80	900	99	17 110	12 900	1.90	0.011	50.4
12^e^	**4b**	100/1/0	80	960	99	11 460	12 750	1.36	0.033	83.2
13^e^	**4b**	150/1/0	80	180	97	16 768	15 400	1.80	0.022	105
14^e^	**4a**	100/1/0	60	1200	98	11 346	6400	1.84	0.0025	8.46
15^e^	**4b**	100/1/0	60	300	71	8264	4400	1.71	0.0061	16.5

a 200 mg of ε-CL; 0.80 mol dm^–3^ in toluene.

b 400 mg of ε-CL; 0.80 mol dm^–3^ in toluene.

c 500 mg of ε-CL; 0.80 mol dm^–3^ in toluene.

d 200 mg of ε-CL; 0.32 mol dm^–3^ in toluene.

e 100 mg of ε-CL; 1.00 mol dm^–3^ in toluene-*d*_8_, in a J Young’s NMR tube, unstirred (kinetic studies).

f Conversion determined via ^1^H NMR spectroscopy by integration of the monomer and polymer OC*H*_2_ methylene resonances.

g *M*_n_^Theo^ calculated from conversion and catalyst concentration,  +  + .

h Determined via GPC analysis in THF using a refractive index detector and with application of a conversion factor of 0.56.^30^

i Determined via ^1^H NMR spectroscopic reaction monitoring.

j TOF calculated using the values of data points closest to 20% conversion and 70% conversion, respectively: .

Most significantly, the rate of polymerization of ε-CL in the presence of **4b** was twice as high as that observed for **4a**, a difference only ascribable to the identity of the metal center (when [initiator]:[ε-CL] = 100:1, *k*_obs_ = 0.017 and 0.033 min^–1^ for **4a** and **4b**, respectively, and when [initiator]:[ε-CL] = 150:1, *k*_obs_ = 0.011 and 0.022 min^–1^ for **4a** and **4b**, respectively). This difference was also observed at 60 °C under otherwise identical conditions ([initiator]:[ε-CL] = 100:1; *k*_obs_ = 0.0025 and 0.0061 min^–1^ for **4a** and **4b**, respectively). Furthermore, when respective solutions of **4a** and **4b** in chloroform-*d* were heated simultaneously to 75 °C, intramolecular nucleophilic attack at the coordinated ε-CL carbonyl group of **4b** by the adjacent alkoxide moiety proceeded more rapidly than in the case of **4a** (observed via ^1^H NMR spectroscopy; see Supporting Information). The origin of the increased activity of the Ta system, relative to Nb, has not been ascertained. However, it is plausible that such a discrepancy may uniquely apply to the cationic nature of the active species, in contrast to all relevant literature comparisons, which are variously concerned with initiators bearing neutral or negatively charged metal centers,^37−39^ and could, for example, find its basis in the character of the metal–alkoxide bond.^51^

**Figure 4:**
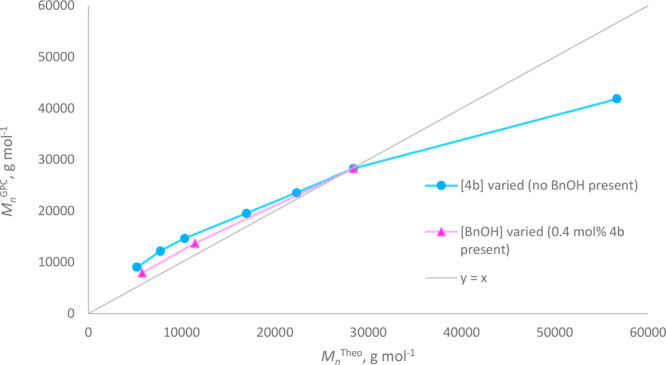
Plot of *M*_n_^Theo^ versus *M*_n_^GPC^ for PCL samples produced in the presence of initiator **4b** (see entries 1–9 in Table 1) and, where relevant, exogenous BnOH, showing good adherence to theoretical values.

When the ROP of ε-CL in the presence of **4b** was carried out on a larger scale, at 80 °C in stirred protio-toluene (≥200 mg of ε-CL), good molecular weight control was observed in response to variation of the catalyst loading, consistent with a well-controlled chain-growth polymerization process, exhibiting key characteristics of living polymerization kinetics (Figure 4). Furthermore, the molecular weight of the PCL product could be reduced in a controlled manner by addition of an exogenous benzyl alcohol nucleophile, while the polymer dispersity, *Đ*_M_, remained low. This is commensurate with the non-rate-determining chain transfer activity indicative of an immortal kinetic regime.^3,16,26−28^ While still narrow, the dispersity, *Đ*_M_, of the polymer products afforded by **4b** was broader than we have previously observed for PCL produced in the presence of **4a**. However, this effect, which is consistent with an increase in the rate of propagation without a concomitant acceleration of the initiation event, was readily ameliorated both when a co-initiator was used and on decreasing the concentration of the reaction mixture. Moreover, increasing the solvent volume allowed the catalyst concentration to be increased, relative to the monomer, without encountering solubility-related issues. Accordingly, PCL samples produced at catalyst loadings of 1.1–2.2 mol % were purified (by precipitation from, and copious washing with, methanol), and ^1^H NMR spectroscopy confirmed the quantitative incorporation of the catalyst’s ethoxide moiety as an ethoxyl end group of the polyester chain, indicative of a coordination–insertion mechanism (where the ratios [**4b**]:[ε-CL] were 45:1, 67.5:1, and 90:1; the ratios of ε-CL units in the PCL backbone to ethoxyl polymer end groups were determined by ^1^H NMR spectroscopy to be 46:1, 65:1, and 90:1, respectively, giving *M*_n_^NMR^ values of 5400, 7570, and 10 80 g mol^–1^, respectively; in close agreement with theoretical values).

### Polymerization of δ-Valerolactone

**Figure 5:**
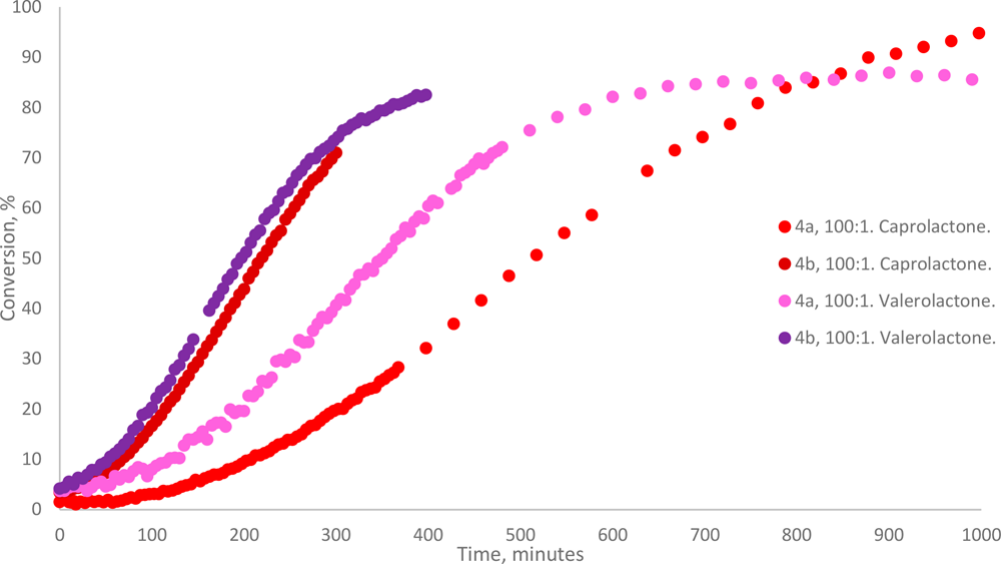
Complete reaction profiles for the ROP of ε-CL and δ-VL at 60 °C in toluene-*d*_8_ in the presence of 1 mol % of initiators **4a** and **4b**.

The ROP of δ-valerolactone was facile in the presence of both catalysts **4a** and **4b**, the latter case representing the first use of a Ta complex as an initiator for the ROP of that monomer (Table 2). Similar to the ROP of ε-CL, **4b** exhibited a near-2-fold increase in catalytic activity relative to **4a**. Furthermore, the observed rate of δ-VL polymerization at 60 °C in toluene-*d*_8_ in the presence of **4a** was significantly higher than that of ε-CL, whereas in the presence of **4b**, there was not a significant difference in the rates of polymerization of the two monomers (Figure 5, Figure 7).

**Table 2:** Polymerization Data for the ROP of δ-VL in the Presence of **4a** and **4b**

entry	catalyst	[δ-VL]/[Cat.]/[BnOH]	temp, °C	duration, min	conversion, %^d^	*M*_n_^Theo^,^e^ g mol^–1^	*M*_n_^GPC^,^f^ g mol^–1^	*Đ*_M_^f^	*k*_obs_,^g^ min^–1^	TOF,^h^ h^–1^
1^a^	**4a**	100/1/0	80	210	75	7669	8150	1.73		
2^b^	**4a**	250/1/0	80	330	80	20 184	11 700	1.88		
3^b^	**4a**	250/1/1.5	80	330	83	8439	8000	1.46		
4^b^	**4a**	250/1/4	80	330	85	4319	4800	1.42		
5^b^	**4a**	350/1/0	80	360	78	27 492	17 200	1.51		
6^b^	**4a**	500/1/0	80	840	96	48 216	24 550	1.48		
7^a^	**4b**	100/1/0	80	120	71	7269	6000	1.70		
8^b^	**4b**	250/1/0	80	180	76	19 182	11 500	1.74		
9^b^	**4b**	250/1/1.5	80	180	75	7638	6000	1.54		
10^b^	**4b**	250/1/4	80	180	79	4019	4400	1.38		
11^b^	**4b**	350/1/0	80	240	80	28 193	13450	1.68		
12^b^	**4b**	500/1/0	80	360	83	41 709	16 600	1.66		
13^c^	**4a**	86/1/0	60	360	68	6015	6250	1.46	0.0041	11.9
14^c^	**4b**	86/1/0	60	810	87	7651	11 750	1.67	0.0075	18.3
15^c^	**4a**	100/1/0	60	1000	86	8770	9300	1.50	0.0036	11.6
16^c^	**4b**	100/1/0	60	400	82	8370	3550	1.71	0.0063	17.2

a 500 mg of δ-VL; 0.90 mol dm^–3^ in toluene.

b 1000 mg of δ-VL; 0.90 mol dm^–3^ in toluene.

c 100 mg of δ-VL; 1.00 mol dm^–3^ in toluene-*d*_8_, in a J Young’s NMR tube, unstirred (kinetic studies).

d Conversion determined via ^1^H NMR spectroscopy by integration of the monomer and polymer OC*H*_2_ methylene resonances.

e *M*_n_^Theo^ calculated from conversion and catalyst concentration,  +  + .

f Determined via GPC analysis in THF using a refractive index detector and with application of a conversion factor of 0.57.^30^

g Determined via ^1^H NMR spectroscopic reaction monitoring.

h TOF calculated using the values of data points closest to 20% conversion and 70% conversion, respectively: .

When the ROP of δ-VL initiated by **4a** or **4b** was carried out in stirred protio-toluene at 80 °C (Table 2), the polymer molecular weights determined by GPC (*M*_n_^GPC^) were typically lower than the theoretical values (*M*_n_^Theo^). With both initiators, however, there was a linear relationship between *M*_n_^Theo^ and *M*_n_^GPC^ (see Supporting Information), allowing the molecular weight to be controlled in a predictable manner. Furthermore, when exogenous benzyl alcohol was present, a controlled reduction in *M*_n_^GPC^ was observed, suggestive of an immortal polymerization process. Values of *Đ*_M_ were comparable for PVL samples produced in the presence of **4a** and **4b** but, in general, described broader molecular weight distributions than those recorded for the ROP of ε-CL (*Đ*_M_ = 1.42–1.88). The latter characteristic we attribute to the relatively slow rate of ε-CL ring opening during initiation in comparison to the rate of δ-VL ring-opening events occurring during propagation. Importantly, this represents a deviation from true living ROP kinetics, although good control of the polymer molecular weight remained accessible, presumably due to the discrepancy between the reactivities of ε-CL and δ-VL being quite modest. Nonetheless, similar to observations made in the ROP of ε-CL, PVL of lower *Đ*_M_ was obtained in the presence of a co-initiator than where this species was absent. Furthermore, an increase in *Đ*_M_ concomitant with the greater reactivity of the bulk monomer feed, relative to that of the lactone initially coordinated at the metal center, indicates that the ring-opening event does not occur in competition with any simultaneous monomer exchange process.

The authors are unaware of any other catalytic system in the presence of which the ROP of one cyclic ester can be accessed via an initiation event predicated upon the quantitative, stoichiometric ring opening of a different cyclic ester. When no co-initiator is employed, this reactivity ensures incorporation of a single ε-CL residue into each polymer chain present in the system. In the case of a kinetic regime under which extensive transesterification activity is unable to proliferate, as indicated for the current protocol by the relatively low *Đ*_M_ values obtained for the ROP of ε-CL and δ-VL, the differentiated monomer residue can be surmised to occupy the position directly adjacent to the initiator-derived ethoxyl end group.

### Synthesis of δ-Valerolactone Adducts of Nb and Ta

**Figure 6:**
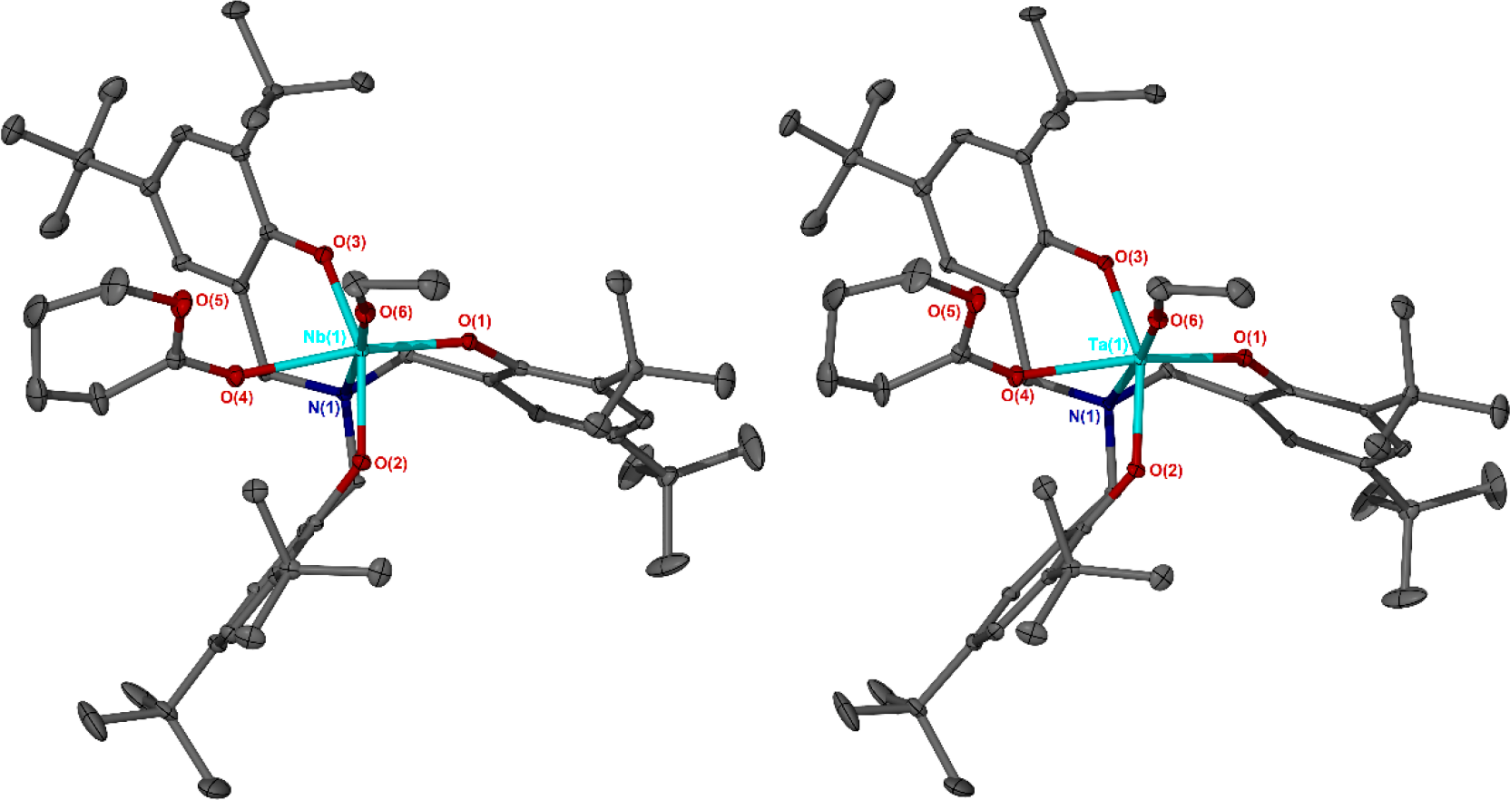
Solid-state structures of the cationic fragments of Nb(V)– and Ta(V)–-δ-valerolactone adducts **5a** and **5b**. Ellipsoids shown at 30% probability level. Hydrogen atoms and SbF_6_^–^ anions have been omitted for clarity. Selected bond lengths (Angstroms) and angles (degrees): **5a** Nb(1)–N(1) 2.343(3), Nb(1)–O(6) 1.833(2), Nb(1)–O(4) 2.167(3), O(4)–C(46) 1.220(5), O(5)–C(46) 1.303(5), O(6)–Nb(1)–N(1) 175.12(10), O(6)–Nb(1)–O(4) 95.83(11), O(1)–Nb(1)–O(4) 162.95(10), O(6)–Nb(1)–O(1) 101.08(11), O(3)–Nb(1)–O(2) 154.49(10), O(3)–Nb(1)–O(4) 81.46(10), O(2)–Nb(1)–O(4) 78.38(10), O(6)–Nb(1)–O(3) 98.78(11), O(6)–Nb(1)–O(2) 98.67(11), O(4)–Nb(1)–N(1) 79.71(10), C(46)–O(4)–Nb(1) 139.3(3); **5b** Ta(1)–N(1) 2.318(4), Ta(1)–O(6) 1.844(3), Ta(1)–O(4) 2.146(3), O(4)–C(46) 1.236(6), O(5)–C(46) 1.283(6), O(6)–Ta(1)–N(1) 175.53(13), O(6)–Ta(1)–O(4) 96.19(13), O(1)–Ta(1)–O(4) 163.72(13), O(6)–Ta(1)–O(1) 99.95(14), O(3)–Ta(1)–O(2) 155.35(12), O(3)–Ta(1)–O(4) 82.03(12), O(2)–Ta(1)–O(4) 78.65(12), O(6)–Ta(1)–O(3) 98.40(14), O(6)–Ta(1)–O(2) 98.77(13), O(4)–Ta(1)–N(1) 79.84(12), C(46)–O(4)–Ta(1) 139.5(3).

Encouraged by the similar reactivity of δ-VL and ε-CL with both **4a** and with **4b**, we have successfully prepared the hexafluoroantimonate salts [L^tBu^Nb(OEt)(δ-VL)]^+^[SbF_6_]^−^, **5a**, and [L^tBu^Ta(OEt)(δ-VL)]^+^[SbF_6_]^−^, **5b**, of δ-VL adducts of amine tris(phenolate)-supported Nb and Ta ethoxides, respectively. **5a** and **5b** were prepared via synthetic methodologies consistent with those employed for ε-CL-bearing species **4a** and **4b**, respectively. Like **4a** and **4b**, both δ-VL adducts, yellow crystalline solid **5a** and colorless crystalline solid **5b**, were prepared in good yield (40% and 58% with respect to **2a** and **2b**, respectively) without any apparent ring opening of the lactone occurring. Analogous to the ε-CL adducts **4a** and **4b**, solid- and solution-state characterization showed complexes **5a** and **5b** to, similarly, be mutually isostructural (Figure 6). Notably, in addition to being only the third and fourth known species in which both an alkoxide initiating group and a cyclic ester are concurrently coordinated at a metal center, following **4a** and **4b**,^36^ to our knowledge, **5a** and **5b** are the first metal complexes of δ-VL to be isolated and represent the first examples of solid-state structures containing δ-VL. The Nb–O and Ta–O bond lengths of the coordinated δ-VL and ethoxide moieties of **5a** and **5b** did not exhibit any statistically significant differences, either with one another or when compared to the relevant bonds of **4a** and **4b**. ^1^H NMR spectroscopy showed that treatment at ambient temperature of a solution of **3a** in chloroform-*d* with excess δ-VL yielded **5a**.

As anticipated, both **5a** and **5b** were active initiators for the ROP of ε-CL and δ-VL (Table 3), the propagating species presumably being identical to those generated in the cases of **4a** and **4b**, respectively. Notably, both **5a** and **5b** afforded PVL of a narrower molecular weight distribution than was produced by the corresponding ε-CL adducts, **4a** and **4b**. Such improved adherence to living ROP kinetics is consistent with a faster initiation event, proceeding via ring opening of the more reactive lactone. This presents the possibility that the dispersity of aliphatic polyesters produced in the presence of systems such as those reported here could be deliberately tuned and, in particular, reduced by careful selection of the coordinated monomer. It is similarly feasible that catalytic application of Nb and Ta complexes bearing coordinated lactones distinct from the monomer feed could enable facile modification of polymer properties through the aforementioned stoichiometric (or, where a co-initiator is used, substoichiometric) synthesis-stage installation of single differentiated monomer residues via intramolecular reaction with the alkoxide initiating group. The authors are unaware of any alternative one-pot routes to achieving precise single-unit differentiation in the ROP of lactones or lactides (ring-opening transesterification polymerization, ROTEP), and while such sequence control has been reported for other polymerization types, such as the ring-opening metathesis polymerization (ROMP) of cyclic olefins,^52−54^ among others,^55,56^ this has typically been reliant upon reactivities in which the addition of the desired single-monomer unit reversibly deactivates the active species.^52,53,55^ One notable exception to this general requirement is Foster and co-workers’ recent report concerning the ROMP of oxanorbornene imide monomers, wherein discrepancies between different monomer stereoisomers’ binding affinity for the Hoveyda–Grubbs catalyst used and the rates at which they respectively underwent propagation were exploited to achieve single-unit addition.^53^ The current system, similarly, does not necessitate such a deactivation event. However, in this case stoichiometric introduction of the differentiated monomer residue instead arises directly as a result of the catalytic induction.

**Table 3:** Polymerization Data for the ROP of δ-VL and ε-CL in the Presence of **5a** and **5b**

entry	catalyst	monomer	duration, min	conversion, %^d^	*M*_n_^Theo^,^e^ g mol^–1^	*M*_n_^GPC^,^f^ g mol^–1^	*Đ*_M_^f^
1^a^	**5a**	δ-VL	375	93	18 768	16 600	1.37
3^a^	**5b**	δ-VL	150	97	19 569	13 600	1.26
3^b^	**5a**	ε-CL	300	>99	11 560	15 250	1.53
4^c^	**5b**	ε-CL	150	99	11 446	13 600	1.45

a 100 mg of δ-VL; 0.80 mol dm^–3^ in toluene at 80 °C. [δ-VL]/[Cat.] = 200.

b 500 mg of ε-CL; 0.80 mol dm^–3^ in toluene at 80 °C. [ε-CL]/[Cat.] = 100.

c 250 mg of ε-CL; 0.80 mol dm^–3^ in toluene at 80 °C. [ε-CL]/[Cat.] = 100.

d Conversion determined via ^1^H NMR spectroscopy, by integration of the monomer and polymer OC*H*_2_ methylene resonances.

e *M*_n_^Theo^ calculated from conversion and catalyst concentration, .

f Determined via GPC analysis in THF using a refractive index detector and with application of a conversion factor of 0.57 (PVL) or 0.56 (PCL).^30^

### Polymerization of *rac*-β-Butyrolactone

The ROP of *rac*-β-butyrolactone readily occurred in the presence of **4a** and **4b** at 80 °C in toluene-*d*_8_. With both initiators, however, the reaction proceeded extremely rapidly, precluding acquisition of useful kinetic data (see Supporting Information). **4a** and **4b** were therefore applied to the ROP of β-BL in toluene-*d*_8_ at 60 °C for kinetic studies. Polymerization reactions of β-BL that were not subject to kinetic analysis were typically undertaken at 65 °C in protio-toluene (see Table S1 in the Supporting Information). Although highly active, neither **4a** nor **4b** exhibited any stereoselectivity, and the molecular weights of the P3HB products obtained were consistently much lower than theoretical values, which were calculated assuming a living coordination–insertion regime with negligible side reactions. Moreover, while *M*_n_^GPC^ was reduced at higher catalyst concentrations, there was a complete absence of satisfactory molecular weight control, with all values being in the range 1350–2100 g mol^–1^ (determined via GPC with refractive index detector, calibrated against polystyrene standards of known molecular weight, with application of a conversion factor).^30^ Falling significantly below *M*_n_^Theo^, the magnitude of these values is reminiscent of the propensity we have previously noted of a *C*_3_-symmetric amine tris(phenolate)-supported Zr isopropoxide initiator to consistently yield low molecular weight P3HB in the ROP of β-BL.^19^ Such similar reactivity may be unsurprising given the surmised isostructural and isoelectronic nature of the propagating species originating from the neutral Group 4 and cationic Group 5 systems. MALDI-TOF-MS analysis confirmed the presence of cyclic P3HB, presumably originating from cyclization of linear, ethoxyl-terminated P3HB via backbiting events (see Supporting Information). Analogous behavior was observed in the presence of Nb catalyst **4a**, although similar to the ROP of ε-CL, the reaction rate was much reduced for that species relative to the heavier congener, **4b**.

**Figure 7:**
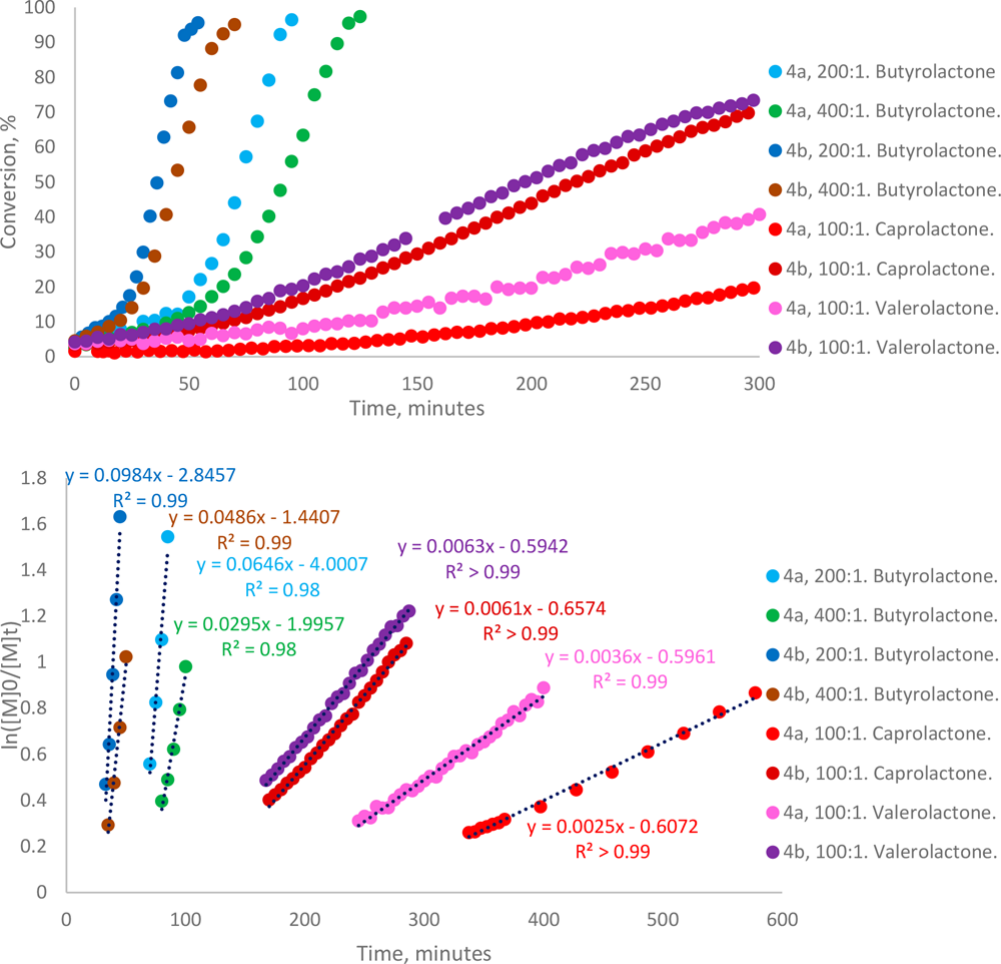
Reaction profiles and semilogarithmic plots for the ROP of β-BL, ε-CL and δ-VL at 60 °C in toluene-*d*_8_ in the presence of initiators **4a** and **4b**. When [**4a**]:[β-BL] = 200:1, *k*_obs_ = 0.065 min^–1^, TOF = 282 h^–1^. When [**4a**]:[β-BL] = 400:1, *k*_obs_ = 0.030 min^–1^, TOF = 379 h^–1^. When [**4b**]:[β-BL] = 200:1, *k*_obs_ = 0.098 min^–1^, TOF = 414 h^–1^. When [**4b**]:[β-BL] = 400:1, *k*_obs_ = 0.049 min^–1^, TOF = 571 h^–1^. When [**4a**]:[ε-CL] = 100:1, *k*_obs_ = 0.0025 min^–1^. When [**4b**]:[ε-CL] = 100:1, *k*_obs_ = 0.0061 min^–1^. When [**4a**]:[δ-VL] = 100:1, *k*_obs_ = 0.0036 min^–1^. When [**4b**]:[δ-VL] = 100:1, *k*_obs_ = 0.0063 min^–1^. TOF for β-BL ROP calculated using the values of data points closest to 30% conversion and 90% conversion, respectively: .

The kinetic profile for the ROP of β-BL at 60 °C in toluene-*d*_8_ in the presence of both **4a** and **4b** consistently described a long induction period followed by high activity until quantitative conversion was reached (Figure 7). Empirically similar profiles were observed at 80 °C, despite the unsatisfactorily low resolution of the kinetic data acquired at that temperature.

**Scheme 2:**
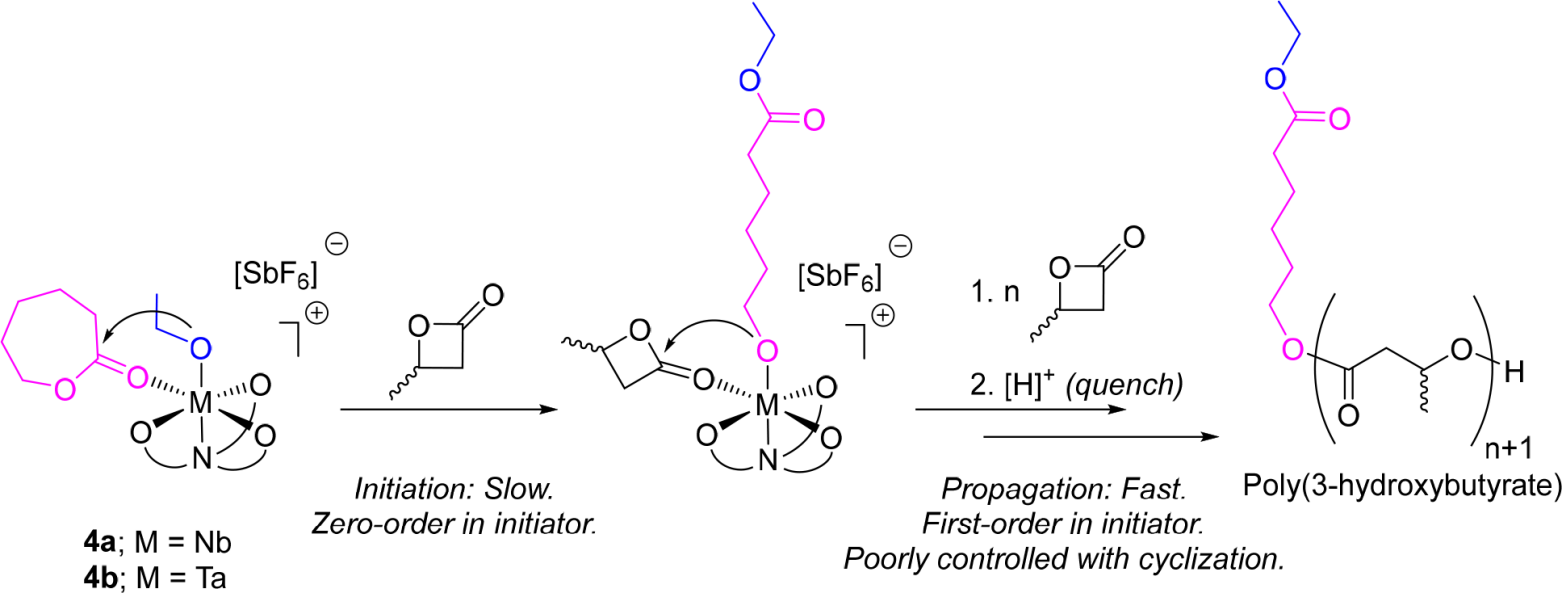
Proposed Mechanism for the ROP of *rac*-β-BL in the Presence of **4a** or **4b**, Proceeding First via the Slow Ring Opening of the Coordinated ε-CL Moiety, Followed by Rapid Coordination–Insertion Polymerization of the Monomer Feed The presence of *n* + 1 repeat units in P3HB product assumes negligible side reactions (cyclization) and ideal living kinetics.

The induction period was considerably longer for **4a** than **4b**, reflecting the known greater reactivity of the Ta system relative to Nb with respect to the ring opening of ε-CL. This observation is, therefore, compatible with initiation occurring via slow intramolecular nucleophilic attack and ring opening of the metal-coordinated ε-CL molecule of **4a** or **4b** with subsequent rapid polymerization of the more reactive β-BL monomer feed (Scheme 2). Significantly, such a low rate of initiation relative to that of propagation is entirely irreconcilable with the characteristics of an ideal living or immortal kinetic regime. Accordingly, both **4a** and **4b** (and **5a** and **5b**) would be anticipated to offer only very poor molecular weight control and to afford P3HB of broad dispersity in the ROP of β-BL, irrespective of the proliferation of cyclization processes and other undesirable side reactions.

Varying the catalyst loading appeared to have no effect on the length of the induction period in the ROP of β-BL for either **4a** or **4b**. This is further consistent with initiation proceeding via the described intramolecular ring-opening event, which is expected to be zero order with respect to the catalyst concentration. Nonetheless, an initiation event predicated first upon displacement of (unreacted) ε-CL at the metal center by the more reactive monomer was considered as an alternative explanation for the delayed attainment of the maximum rate in the ROP of β-BL. However, the induction periods corresponding to the use of both **4a** and **4b**, respectively, were determined to be sufficiently long for stoichiometric (intramolecular) ring opening of the metal-coordinated ε-CL to have occurred, based on observations made in the course of catalytic studies described elsewhere in this work and given that *k*_initiation_ ≥ *k*_propagation_ for the ROP of ε-CL. Additionally, the absence of any significant conversion of the highly polymerizable β-BL feed during the induction period suggests that any possible monomer exchange at the metal center could not have taken place on a sufficiently short time scale for ε-CL to have remained completely unreacted. Moreover, when the ROP of β-BL in protio-toluene in the presence of 0.5 mol % **4b** was quenched at 65% conversion (15 min, ∼130 equiv of β-BL ring opened, with respect to the catalyst), the ^1^H NMR spectrum of the purified P3HB product also contained signals corresponding to a ring-opened ε-CL residue in an approximate molar ratio of 130:1 relative to the resonances originating from the P3HB backbone. This quantitative incorporation of the much less reactive monomer species, while a 70-fold excess of unreacted β-BL remained present, is indicative of initiation proceeding exclusively via ring opening of the coordinated ε-CL moiety rather than ε-CL only being fully incorporated into the polymer chain following complete consumption of the significantly more reactive β-BL feed.

Whereas the activities of both **4a** and **4b** were negligible at 25 °C in chloroform-*d*, the hexafluoroantimonate salt, **3a**, of a monocationic, monoalkoxo, bimetallic Nb complex readily polymerized β-BL both at 60 °C in toluene-*d*_8_ and at 25 °C in chloroform-*d*, proceeding in both cases without a significant induction period (see Supporting Information). As we have reported previously for the ROP of ε-CL,^36^ the precatalyst **3a** is activated via cleavage of a labile Nb−μ_2_F bond and coordination of the monomer to the cationic fragment, the active propagating species being identical with that for both **4a** and **5a**. Accordingly, the lack of an induction period associated with deployment of this species to the ROP of β-BL further confirms the role of the coordinated ε-CL molecule of **4a** and **4b** in the slow initiation observed for those systems, with monomer exchange at the coordinatively saturated metal center clearly being unfavorable.

As anticipated, **3a** consistently yielded low molecular weight, cyclic P3HB at all reaction temperatures assessed, and there was no indication that the system was adherent to a living kinetic regime. However, values of *M*_n_^GPC^ were generally slightly higher than those obtained in the presence of **4a** and **4b** (*M*_n_^GPC^ ≤ 3000 g mol^–1^; DP ≤ 35 for cyclic species). While the kinetic profile of the ROP of β-BL in the presence of **3a** demonstrated qualitatively that there was, indeed, no clear induction period, the poor solubility of the highly crystalline precatalyst in toluene-*d*_8_ at 60 °C and its empirically very high catalytic activity together precluded attainment of a consistent rate. Accordingly, determination of a valid rate constant pertaining to the use of **3a**, for comparison with the activity of **4a** under the conditions used for kinetic studies, was not possible. Although **3a** is presumably able to deliver P3HB on a shorter time scale than **4a** or **4b** at an equimolar catalyst concentration, by elimination of the induction period, the maximum rate cannot, presumably, exceed that of **4a**, the active species being identical. Furthermore, activation of the bimetallic cation of **3a** is not atom economical, cleavage yielding two Nb amine tris(phenolate) fragments, of which only the cationic, alkoxide-bearing one is catalytically active.^36^ Attempts to prepare an analogous Ta-based system, [{L^tBu^Ta(OEt)}-μ_2_F-{L^tBu^TaF}]^+^[SbF_6_]^−^, by an equivalent synthetic route to that used for the preparation of **3a** were consistently unsuccessful, this being reconcilable with the greater reactivity of the Ta systems relative to their Nb analogues observed throughout the current work.

A cursory study has shown Nb and Ta δ-VL adducts **5a** and **5b** to be active initiators for the ROP of β-BL, producing low molecular weight P3HB, similar to **4a** and **4b**. Accordingly, further β-BL polymerization studies were not undertaken with **5a** and **5b**.

## Conclusions

We have prepared an ε-caprolactone adduct of a cationic amine tris(phenolate)-supported tantalum ethoxide complex, structurally identical to our previously reported niobium system.^36^ In the controlled ring-opening polymerization of ε-caprolactone, the Ta system unexpectedly exhibited much higher activity than the Nb congener. Both the Nb and the Ta species were also active for the ROP of *rac*-β-butyrolactone and of δ-valerolactone, affording facile molecular weight control in the latter case. Consistent with the polymerization of ε-caprolactone, the Ta species exhibited a significantly increased rate, relative to the Nb system, in the ROP of both rac-β-butyrolactone and δ-valerolactone. The expansion of the monomer scope in this work, in comparison to our previous studies,^36^ and differentiation of the metal-coordinated lactone from the wider monomer feed has permitted further illumination of the initiation mechanism and specifically of the intramolecular insertion event. This work represents the first examples of the Group 5-catalyzed ROP of *rac*-β-butyrolactone and of the tantalum-catalyzed ROP of δ-valerolactone. We have also prepared cationic δ-valerolactone adducts **5a** and **5b** of ethoxo–Nb and ethoxo–Ta amine tris(phenolate) complexes, respectively. Exhibiting similar reactivity to ε-caprolactone adducts **4a** and **4b**, those species are, to our knowledge, the first examples of metal complexes of δ-valerolactone and the first solid-state structures containing a molecule of δ-valerolactone. Both **5a** and **5b** are active initiators for the ROP of *rac*-β-butyrolactone, δ-valerolactone, and ε-caprolactone. Moreover, the preparation and catalytic application of structurally analogous Nb and Ta complexes bearing different coordinated lactones offers both further insight into the mechanism of lactone ROP initiation via coordination–insertion and a potentially useful new capability for controlled polymer synthesis. Specifically, the rate of the initiation event can be controlled independently of that of propagation, facilitating manipulation of polymer dispersity, and quantitative, synthesis-stage installation of a single-monomer residue, distinct from those comprising the bulk monomer feed, is readily achievable.

## Supporting Information Available

Complete synthetic procedures and characterization data for Nb and Ta species reported herein, polymerization procedures, polymer characterization data, further discussion, and kinetic data; relevant safety statements are included in the Materials and Methods section (PDF)
