# Supplementary material for: Unexpected Periodicity in Cationic Group 5 Initiators for the Ring-Opening Polymerization of Lactones
Source: Inorg Chem. 2023 Dec 20;63(1):27–38. doi: 10.1021/acs.inorgchem.3c03854 (PMC10777398; doi:10.1021/acs.inorgchem.3c03854)
Supplement: Supplementary file 1 — ic3c03854_si_001.pdf [file ic3c03854_si_001.pdf]

Supporting Information

Unexpected Periodicity in Cationic Group 5 Initiators for the Ring-Opening Polymerization of Lactones

Antoine Buchard,[a,b] Matthew G. Davidson,[a,b] Gerrit Gobius du Sart,[c] Matthew D. Jones,[a,b] Gabriele Kociok-Köhn,[d] Strachan N. McCormick*[a,b] and Paul McKeown,[b]*

[a] Institute for Sustainability, University of Bath, Bath BA2 7AY, United Kingdom

[b] Department of Chemistry, University of Bath, Bath BA2 7AY, United Kingdom

[c] TotalEnergies Corbion, Stadhuisplein 70, 4203 NS Gorinchem, The Netherlands

[d] Material and Chemical Characterization and Analysis Facility (MC^2), University of Bath, Bath BA2 7AY, United Kingdom

E-mail: S.N.McCormick@bath.ac.uk, M.G.Davidson@bath.ac.uk

Contents

Materials and methods 4

Synthetic procedures and analytical data 7

Synthesis of amine tris(phenolate) pro-ligands 7

Synthesis of pro-ligand tris(2-hydroxy-3,5-di-*tert*-butylbenzyl)amine, H_3L^{tBu} 7

Synthesis of niobium and tantalum amine tris(phenolate) complexes 7

Synthesis of niobium species [$L^{tBu}Nb(OEt)_2$], 1a 7

Synthesis of niobium species [$L^{tBu}Nb(OEt)Cl$], 2a 7

Synthesis of tantalum species [$L^{tBu}Ta(OEt)_2$], 1b 8

Synthesis of tantalum species [$L^{tBu}Ta(OEt)Cl$], 2b 11

Synthesis of niobium species [{$L^{tBu}NbF$}-μ_2F-{$L^{tBu}Nb(OEt)$}]$^+$[SbF_6]$^-$, 3a 14

Synthesis of niobium species [$L^{tBu}Nb(OEt)$(ε-CL)]$^+$[SbF_6]$^-$, 4a 14

Synthesis of tantalum species [$L^{tBu}Ta(OEt)$(ε-CL)]$^+$[SbF_6]$^-$, 4b 14

Synthesis of niobium species [$L^{tBu}Nb(OEt)$(δ-VL)]$^+$[SbF_6]$^-$, 5a 18

Synthesis of tantalum species [$L^{tBu}Ta(OEt)$(δ-VL)]$^+$[SbF_6]$^-$, 5b 24

Stoichiometric Experiments 29

Preparation of 5a from 3a by addition of δ-valerolactone 29

Thermal stability of Nb complex 4a and Ta complex 4b 30

Polymerisation studies 32

Example polymerisation procedure 32

Polymer analysis 32

General procedure for kinetic studies 33

Polymer analysis 33

Polymerization of δ-valerolactone 34

Polymerization of *rac*-β-butyrolactone 35

Kinetics of *rac*-β-butyrolactone ROP at 80 °C in toluene-*d8* 37

Comparison of *rac*-β-butyrolactone ROP kinetics in the presence of 3a and 4a, at 60 °C in toluene-*d8* 38

Comparison of of *rac*-β-butyrolactone ROP kinetics in the presence of 3a, 4a and 4b, at 25 °C in $CDCl_3$ 39

Additional data for δ-valerolactone ROP at 60 °C in toluene-*d8* 40

Control experiments 41

Attempted ROP of *L*-lactide and *rac*-lactide 41

Attempted catalytic use of neutral Nb and Ta complexes for ROP of lactones 41

End-group analysis 42

MALDI-TOF-MS spectra 45

Example GPC Traces 51
Crystallographic Parameters 56
References 56

Materials and methods

Caution! *All solvents and reagents should be handled in accordance with standard laboratory procedures, and according to their associated GHS Hazard Statements and Precautionary Statements. Reagents presenting GHS category 1 hazards (including sub-categories 1A and 1B) are specifically identified below.*

Caution! *Chlorotrimethylsilane presents a GHS category 1 hazard of serious eye damage, and a GHS sub-category 1A hazard of skin corrosion.*

Caution! *Niobium(V) ethoxide presents a GHS category 1B hazard of skin corrosion.*

Caution! *2,4-di-tert-butylphenol presents GHS category 1 hazards of serious eye damage, short-term aquatic hazard, and long-term aquatic hazard.*

Caution! *Paraformaldehyde presents GHS category 1 hazards of serious eye damage and skin sensitization, and a GHS sub-category 1B hazard of carcinogenicity.*

Caution! *Hexamethylenetetramine presents a GHS sub-category 1B hazard of skin sensitization.*

Caution! *δ-Valerolactone presents a GHS category 1 hazard of serious eye damage.*

Caution! *β-Butyrolactone is a suspected carcinogen (GHS Category 2) and should be handled with care to avoid exposure.*

Caution! *Use of a Schlenk line (vacuum manifold) requires care due to the presence of a liquid nitrogen-cooled solvent trap, and the hazards associated both with the use of a cryogenic fluid and with the potential for condensation of liquid oxygen in the presence of organic solvents.*

Caution! *Transfer, handling and, specifically, syringe filtration of hazardous materials in a glove box with use of hypodermic needles should be undertaken with care to mitigate the risk of personal injury.*

Caution! *All Niobium(V) and Tantalum(V) complexes prepared in the current work should be assumed to be highly toxic by all exposure routes and handled according to best laboratory practice. In particular, hexafluoroantimonate salts **3a**, **4a**, **4b**, **5a** and **5b** have been observed to be highly Lewis acidic and, accordingly, may readily react with biological tissues on exposure.*

Manipulations, unless otherwise specified, were carried out under an atmosphere of dry argon using standard Schlenk line and glove box techniques. An MBraun glove box was used, equipped with internal taps supplying dry toluene, dichloromethane and hexane directly from an adjacent MBraun solvent purification system (SPS). The glove box atmosphere was maintained with O_2 and H_2O concentrations <0.1 ppm respectively.

Tetrahydrofuran, toluene, dichloromethane and hexane were drawn from an MBraun solvent purification system and stored over molecular sieves under a dry argon atmosphere unless otherwise specified. Anhydrous pentane was purchased from Sigma Aldrich and stored over molecular sieves under a dry argon atmosphere.

Niobium(V) ethoxide, $Nb(OEt)_5$, and silver hexafluoroantimonate, $AgSbF_6$, were purchased from Strem Chemicals (UK) and used under a dry argon atmosphere without further purification. Tantalum(V) ethoxide, $Ta(OEt)_5$, was purchased both from Fischer Scientific and Strem Chemicals (UK), and used under a dry argon atmosphere without further purification. Chlorotrimethylsilane, TMSCl, was purchased from Sigma Aldrich and used under a dry argon atmosphere without further purification.

Anhydrous benzyl alcohol was purchased from Sigma Aldrich and degassed under dynamic vacuum for 20 h before storage and use under a dry argon atmosphere. 2,4-dimethylphenol, 2,4-di-*tert*-butylphenol, hexamethylenetetramine (HMTA), and para-formaldehyde were all purchased from Sigma Aldrich and used without further purification.

rac-lactide was purchased from Sigma Aldrich and recrystallized 3 times from dry toluene. This was carried out under ambient air, and each recrystallization was followed by washing sparingly with dry toluene over a sintered glass frit. The recrystallized *rac*-lactide was then dried under dynamic vacuum for 16 h and stored under a dry argon atmosphere. *L*-lactide was supplied by Total Corbion and purified in the same manner described for *rac*-lactide. ε-caprolactone was purchased from Sigma Aldrich and dried over calcium hydride, then filtered and vacuum distilled before storage and use under a dry argon atmosphere. δ-valerolactone was purchased from Acros Organics, and dried over calcium hydride, then filtered and vacuum distilled before storage and use under a dry argon atmosphere. β-butyrolactone was purchased from TCI, and dried over calcium hydride, then filtered and vacuum distilled before storage and use under a dry argon atmosphere.

All NMR spectra were acquired using a 400 MHz (^{1}H), 101 MHz (^{13}C), or 500 MHz (^{1}H), 126 MHz (^{13}C) Bruker Avance spectrometer, unless otherwise stated. $CDCl_3$ and C_6D_6 were purchased from Sigma Aldrich and used as received for polymer analysis. For analysis of metal complexes, $CDCl_3$ was dried over calcium hydride, distilled under vacuum, and stored over 4 Å molecular sieves under a dry argon atmosphere. Toluene-d_8 was purchased from Sigma Aldrich and stored over 4 Å molecular sieves under a dry argon atmosphere. Processing of all NMR data, including kinetic studies, was carried out using Mestrelab Research MestReNova Version 11.0.2-18153.

CHN (elemental) microanalysis services were performed, variously, by Mr Stephen Boyer of London Metropolitan University, or by Elemental Lab, a division of Elemental Microanalysis Ltd, of Okehampton, Devon UK.

All crystallographic data was collected on a SuperNova, EOS detector diffractometer using radiation Cu-Kα (λ = 1.54184 Å) or Mo-Kα (λ = 0.71073 Å) all recorded at 150(2) K. All structures were solved by direct methods and refined on all F2 data using the SHELXL-2014 suite of programs. All hydrogen atoms were included in idealized positions and refined using the riding model.

Special refinement details:

1b: One t-Bu (C31/A) group shows rotational disorder in the ratio 77:23. One OEt (O1) group shows rotational disorder in the ratio 70:15:15. One solvent molecule is disordered over two sites sitting on a 4-fold axis. Atoms therein have been refined with 1/8 occupation. All disordered atoms have been refined with ADP restraints.

2b: Cl(1) disordered over two sites in a ratio of 80:20.One tBu group (C38) also disordered over two positions (70:30), the minor component has been left isotropic. Crystal is twinned by rotation of 179.9° around [0.99 -0.00 -0.13] (reciprocal) or [1.00 0.00 -0.00] (direct).

5a: Unit cell dimensions are close to that of an orthorhombic crystal system (β = 90.0800(10)°). However, solving and refinement of the data as a monoclinic system (β = 90)°) yielded the best results and unambiguous structure determination.

4b: Two molecules of toluene contained within unit cell, one of which is disordered and has been treated isotropically. Relatively large q peak related to Sb atom centre.

5b: One tBu group (C11) had less than ideal anisotropic parameters for the methyl carbons.

Polymer molecular weight data was acquired using an Agilent 1260 Gel Permeation Chromatography (GPC) system with refractive index detector, and calibrated against 12 polystyrene standards. A PLgel 5 µm MIXED-D 300 x 7.5 mm column was used, with a PLgel 5 µm MIXED Guard 50 x 7.5 mm guard column. The mobile phase was THF, at a flow rate of 1 ml min^{-1}. Columns and detectors were maintained at 35 °C. Data was processed using Agilent's GPC/SEC Software, Revision A.02.01.

Matrix-assisted laser-desorption time-of-flight (MALDI-ToF) analysis was performed using a Bruker Autoflex speed instrument using a DCTB matrix (trans-2-[3-(4-tertbutylphenyl)-2-methyl-2-propenylidene]malononitrile) and NaTFA to ionise the sample.

High-resolution mass spectra were acquired using a MaXis HD quadrupole electrospray time-of-flight (ESI-QTOF) mass spectrometer (Bruker Daltonik GmbH, Bremen, Germany) for infusions. Analyses were performed in ESI positive mode. The capillary voltage was set to 4500 V, nebulizing gas at 0.4 bar, drying gas at 4 L/min at 180°C. The TOF scan range was from 300 – 2500 mass-to-charge ratio (m/z). Infusions were performed at 3µL/min. The MS instrument was calibrated using sodium formate calibrant solution. The calibrant solution consisted of 3 parts of 1 M NaOH to 97 parts of 50:50 water:isopropanol with 2% formic acid. The observed mass and isotope pattern matched the corresponding theoretical values as calculated from the expected elemental formula within 2 ppm mass accuracy. Mass features were detected as [M]+ ions. Data processing was performed using the Compass Data Analysis software version 4.3 (Bruker Daltonik GmbH, Bremen, Germany). Samples were dissolved in methanol at a concentration of 10 µg mL^{-1}, and positive ionisation mode was used.

Synthetic procedures and analytical data

Synthesis of amine tris(phenolate) pro-ligands

Synthesis of pro-ligand tris(2-hydroxy-3,5-di-*tert*-butylbenzyl)amine, H_3L^{tBu}

Pro-ligand H_3L^{tBu} was synthesised according to the following adapted literature procedure.[1–4] To hexamethylenetetramine (97 mmol, 13.5 g, 1 equivalent), was added 2,4-di-*tert*-butylphenol (1163 mmol, 40 g, 12 equivalents) paraformaldehyde (775 mmol, 23.3 g, 8 equivalents), and deionised water (3.89 mol, 71.1 ml, 40 equivalents). The mixture was then refluxed with vigorous stirring for 120 hours in an oil bath at 150 °C, with further 10 ml aliquots of 2,4-dimethylphenol added daily. The reaction mixture was cooled and the resulting yellow-white solid washed over a glass frit with MeOH (5 x 800 ml), to yield a white powder. The powder was then dried under dynamic vacuum for 24 hours. ^{1}H and $^{13}C\{^{1}H\}$ NMR data in $CDCl_3$ was in agreement with the literature.[1]

Yield: 171 g, 65 %

Synthesis of niobium and tantalum amine tris(phenolate) complexes

Synthesis of niobium species [$L^{tBu}Nb(OEt)_2$], 1a

1a was prepared according to literature procedure, and all characterisation data was consistent with literature values.[3]

Synthesis of niobium species [$L^{tBu}Nb(OEt)Cl$], 2a

2a was prepared according to literature procedure, and all characterisation data was consistent with literature values.[3]

Synthesis of tantalum species [$L^{tBu}Ta(OEt)_2$], 1b

1b was synthesized according to the following adapted literature procedure:[5] To a solution of pro-ligand H_3L^{tBu} (24.6 mmol, 16.55 g) in THF (20 ml), a solution of $Ta(OEt)_5$ (24.6 mmol, 10.00 g) in THF (10 ml) was added by cannula. The resulting mixture was stirred at ambient temperature for 16 hours. The solvent was then removed under dynamic vacuum, until the total volume was ~25 ml. After standing for 16 hours crystals of **1b** suitable for diffraction were obtained. The remaining solvent was then removed under dynamic vacuum, and the solid material washed with dry pentane (~20 ml) and dried under dynamic vacuum. Further crystalline product, **1b**, was isolated from the washings.

Yield: 19.75 g, 85 %

Spectroscopic data is included for ease of comparison with related species described in the current work.

^{1}H NMR (400 MHz, $CDCl_3$, 298 K, δ_H, ppm); 7.26 (1H, *s*, *obscured by residual $CHCl_3$,* Ar*H*), 7.24 (2H, *s*, Ar*H*), 6.94 (2H, *s*, Ar*H*), 6.86 (1H, *s*, Ar*H*), 4.85 (2H, *q*, *J* = 6.98 Hz, OCH_2), 3.62-3.90 (6H, *broad*, NCH_2) , 3.53 (2H, *q*, *J* = 6.96 Hz, OCH_2), 1.51 (9H, *s*, $C(CH_3)_3$), 1.44 (3H, *t*, *J* = 6.98 Hz, OCH_2CH_3), 1.39 (18H, *s*, $C(CH_3)_3$), 1.28 (18H, *s*, $C(CH_3)_3$), 1.23 (9H, *s*, $C(CH_3)_3$), 0.55 (3H, *t*, *J* = 6.96 Hz, OCH_2CH_3). **^{13}C NMR** (101 MHz, $CDCl_3$, 298 K, δ_C, ppm); 156.1 (*Ar*O), 155.8 (*Ar*O), 141.5 (*Ar*), 141.2 (*Ar*), 137.8 (*Ar*), 137.2 (*Ar*), 125.2 (*Ar*H), 124.4 (*Ar*H), 123.9 (*Ar*H), 123.7 (*Ar*H), 123.5 (*Ar*H), 70.2 (OCH_2), 65.9 (OCH_2), 62.3 (NCH_2), 61.8 (NCH_2), 35.1 ($C(CH_3)_3$), 34.3 ($C(CH_3)_3$), 34.3 ($C(CH_3)_3$), 31.9 ($C(CH_3)_3$), 31.9 ($C(CH_3)_3$), 30.1 ($C(CH_3)_3$), 19.1 (OCH_2CH_3), 18.5 (OCH_2CH_3).

Elemental (CHN) Analysis (Calculated, for $C_{49}H_{76}NO_5Ta$); C: 62.60 %, H: 8.15 %, N: 1.49 %, (Experimental); C: 62.64 %, H: 8.28 %, N: 1.64 %.

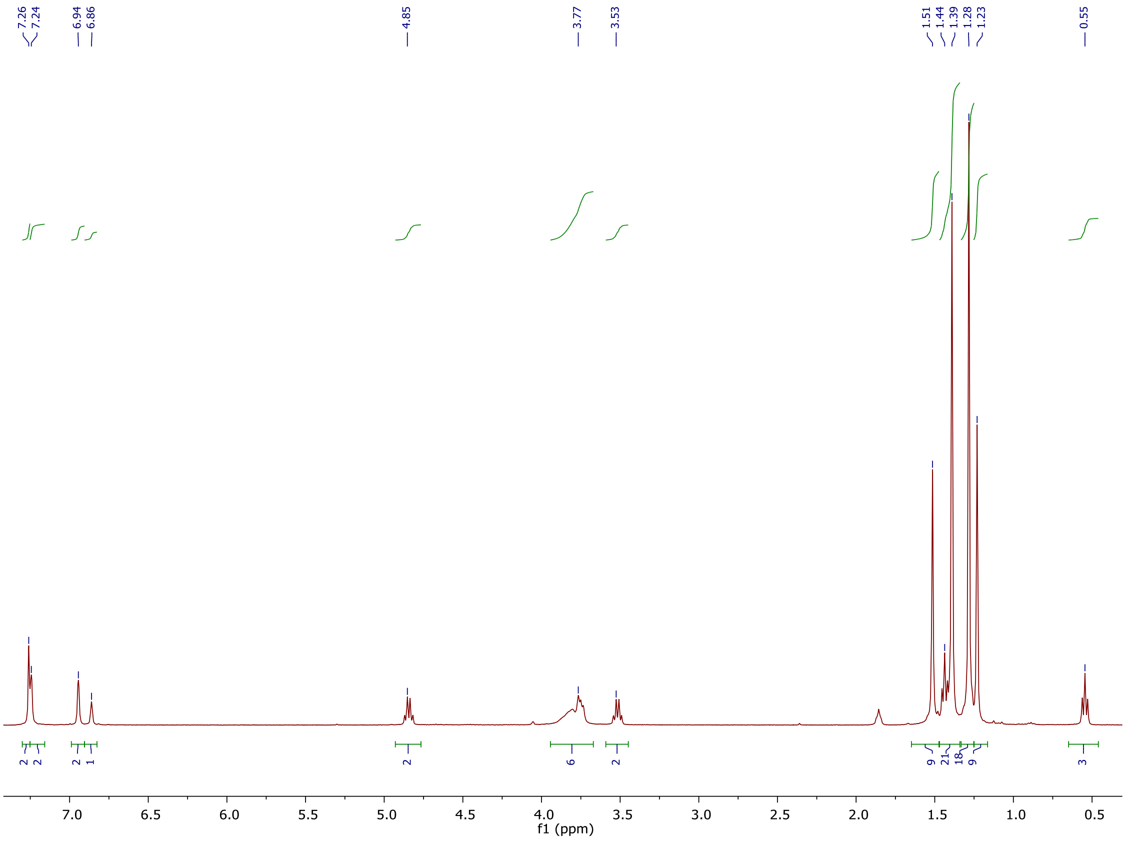

Figure S1. ^{1}H NMR spectrum of **1b** at 298 K in $CDCl_3$

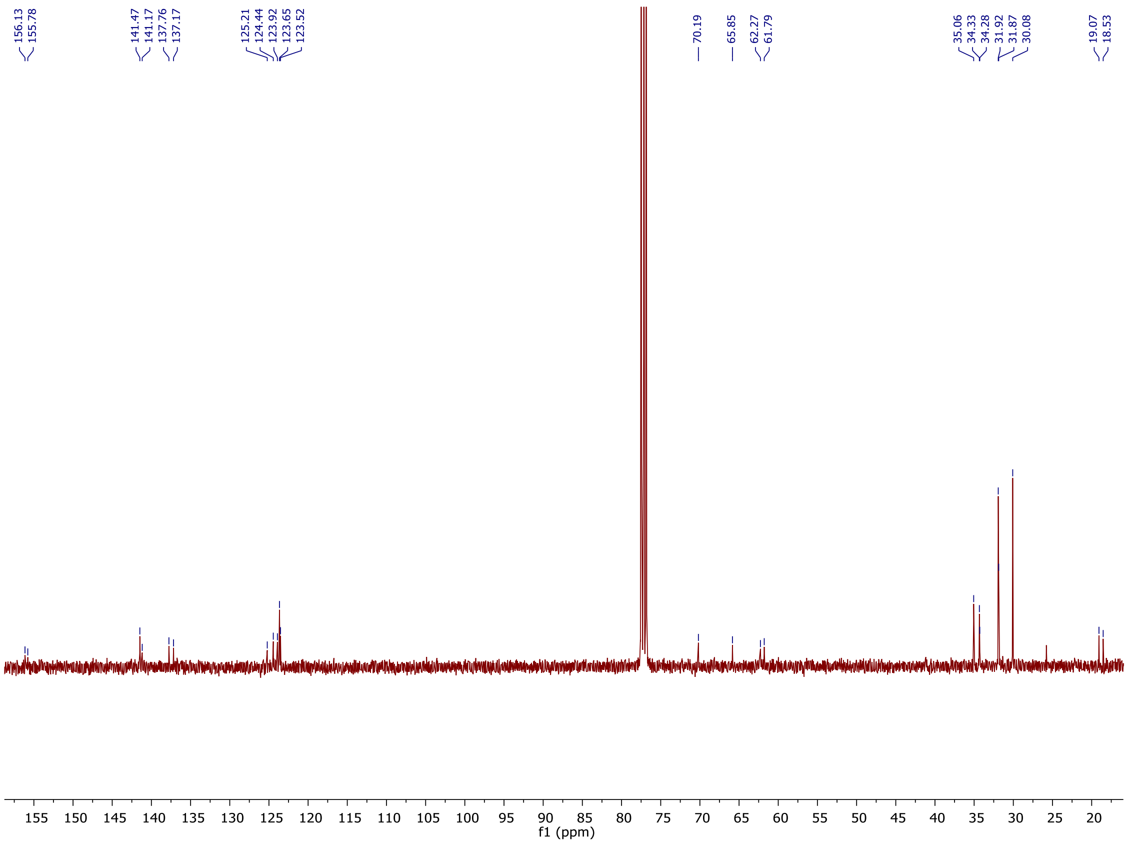

Figure S2. $^{13}C\{^1H\}$ NMR spectrum of **1b** at 298 K in $CDCl_3$

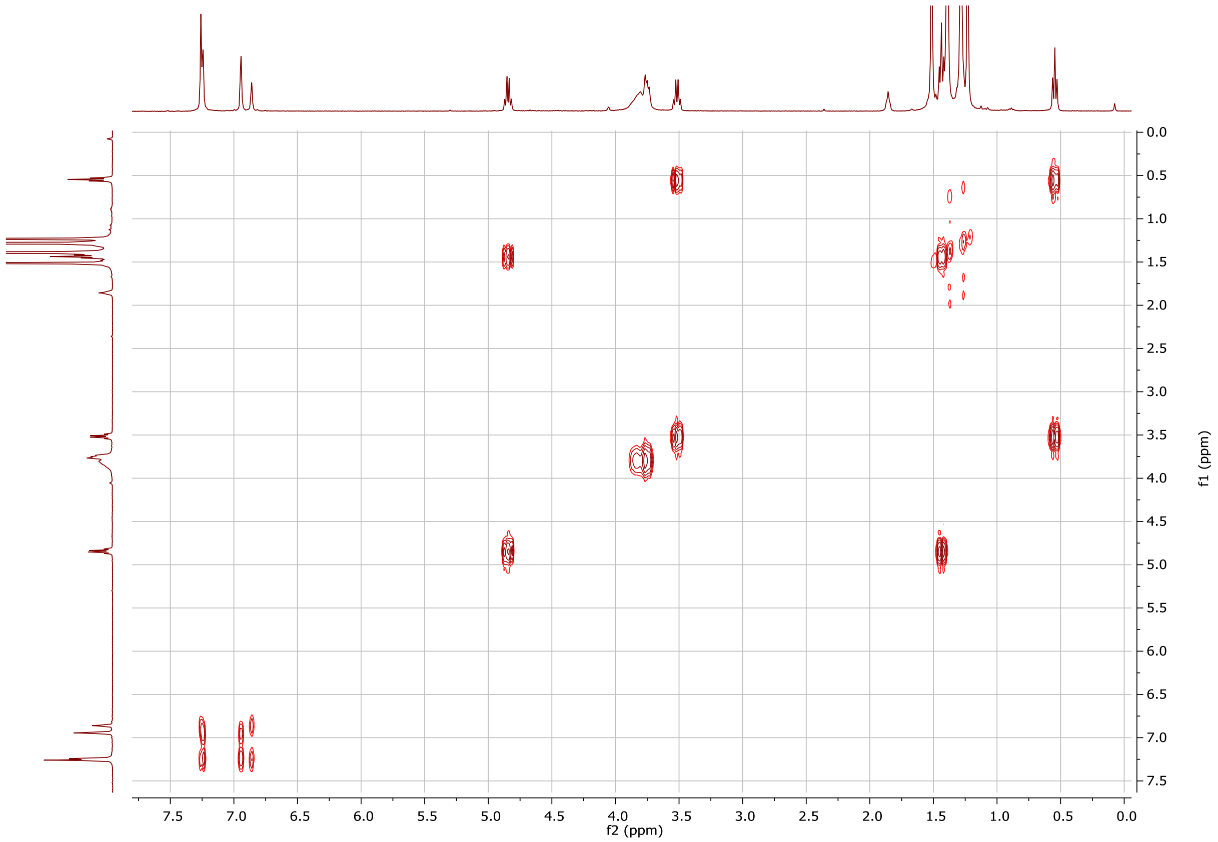

Figure S3. COSY NMR spectrum of **1b** at 298 K in $CDCl_3$

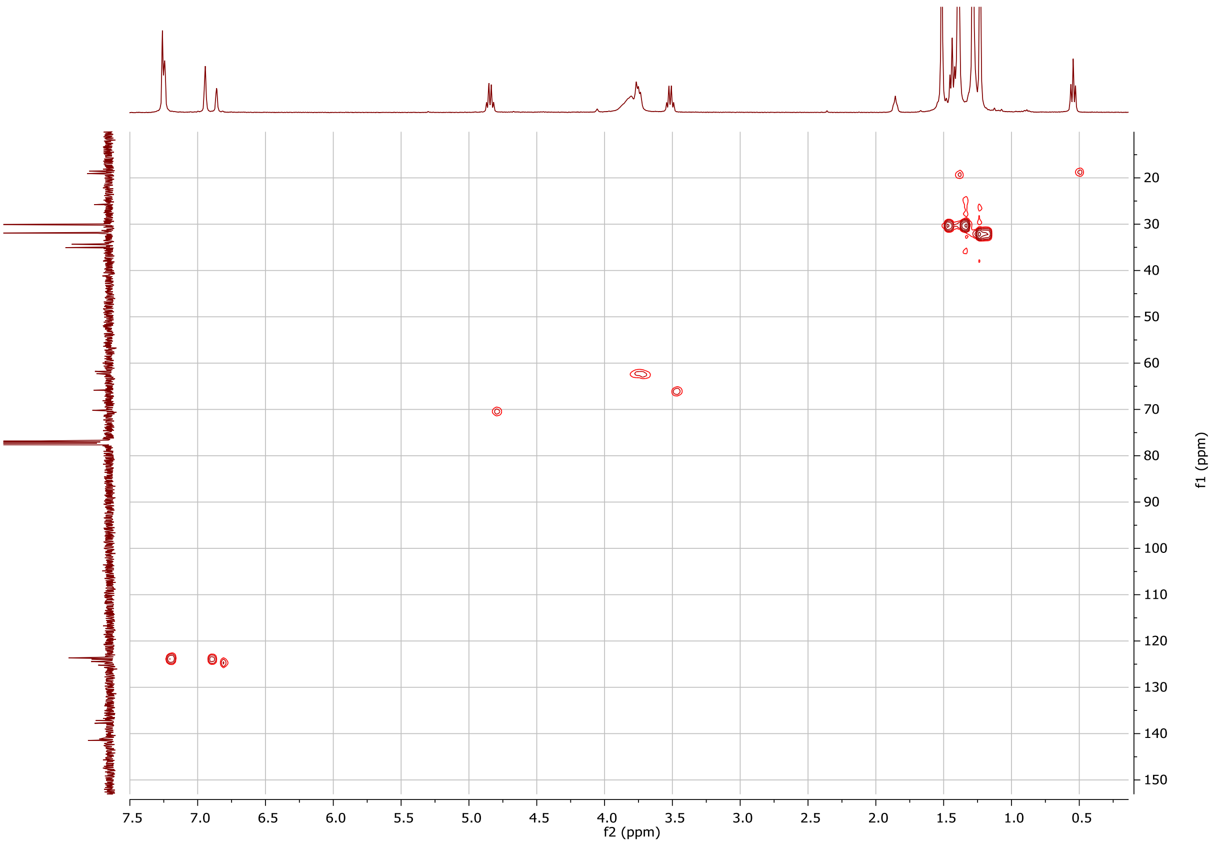

Figure S4. HSQC NMR spectrum of **1b** at 298 K in $CDCl_3$

Synthesis of tantalum species [L^{tBu}Ta(OEt)Cl], 2b

A large excess of chlorotrimethylsilane (TMSCl) (46.6 mmol, 5.00 ml) was added by syringe to a solution of **1b** (10.6 mmol, 10.00 g) in DCM (20 ml). The mixture was stirred at ambient temperature for 16 hours, before removal of volatiles under dynamic vacuum, washing with dry pentane, and drying under dynamic vacuum. ^{1}H analysis of the solid product (6.80 g) showed only 10% conversion to **2b**. The solid material was therefore re-dissolved in DCM (10 ml), in a Schlenk flask fitted with a J Young's PTFE tap. TMSCl (6.00 ml, 55.9 mmol) was added, and the vessel sealed and heated to 55 °C for 3 days. The resulting yellow solution was then transferred by cannula to a Schlenk flask, and the solvent removed under dynamic vacuum to ~15 ml. The solution was then transferred by filter cannula to another Schlenk flask, and the solvent removed under dynamic vacuum. The yellow solid produced was then dissolved in minimum dry pentane and allowed to crystallise. Crystals of **2b** suitable for diffraction were obtained. The solvent was then removed by cannula transfer, and the solid material dried under dynamic vacuum.

Yield: 4.0 g, 40 %

^{1}H NMR (400 MHz, $CDCl_3$, 298 K, δ_H, ppm); 7.29 (2H, *d*, *J* = 1.97 Hz, Ar*H*) 7.27 (1H, *d*, *J* = 2.02 Hz, Ar*H*), 6.97 (2H, *d*, *J* = 1.92 Hz, Ar*H*), 6.89 (1H, *d*, *J* = 2.02 Hz, Ar*H*), 5.00 (2H, *q*, *J* = 7.03 Hz, OC*H₂*), 4.34 (2H, *d*, *J* = 13.28 Hz, NCH_2), 3.82 (2H, *s*, NCH_2), 3.73 (2H, *d*, *J* = 13.84 Hz, NCH_2), 1.55 (3H, *t*, *J* = 7.03 Hz, OCH_2CH_3), 1.48 (9H, *s*, $C(CH_3)_3$), 1.42 (18H, *s*, $C(CH_3)_3$), 1.30 (18H, *s*, $C(CH_3)_3$), 1.22 (9H, *s*, $C(CH_3)_3$). **^{13}C NMR** (101 MHz, $CDCl_3$, 298 K, δ_C, ppm); 155.0 (*Ar*O), 154.7 (*Ar*O), 143.4 (*Ar*), 143.3 (*Ar*), 137.6 (*Ar*), 137.5 (*Ar*), 125.4 (*Ar*H), 124.9 (*Ar*H), 124.4 (*Ar*H), 124.0 (*Ar*H), 123.9 (*Ar*H), 73.0 (O*C*H₂), 63.5 (N*C*H₂), 61.4 (N*C*H₂), 35.1 ($C(CH_3)_3$), 35.0 ($C(CH_3)_3$), 34.4 ($C(CH_3)_3$), 34.4 ($C(CH_3)_3$), 31.9 ($C(CH_3)_3$), 31.8 ($C(CH_3)_3$), 30.4 ($C(CH_3)_3$), 30.2 ($C(CH_3)_3$), 18.4 (OCH_2CH_3).

Elemental (CHN) Analysis (Calculated, for $C_{47}H_{71}NO_4TaCl$); C: 60.67 %, H: 7.69 %, N: 1.51 %, (Experimental); C: 60.02 %, H: 7.62 %, N: 1.69 %.

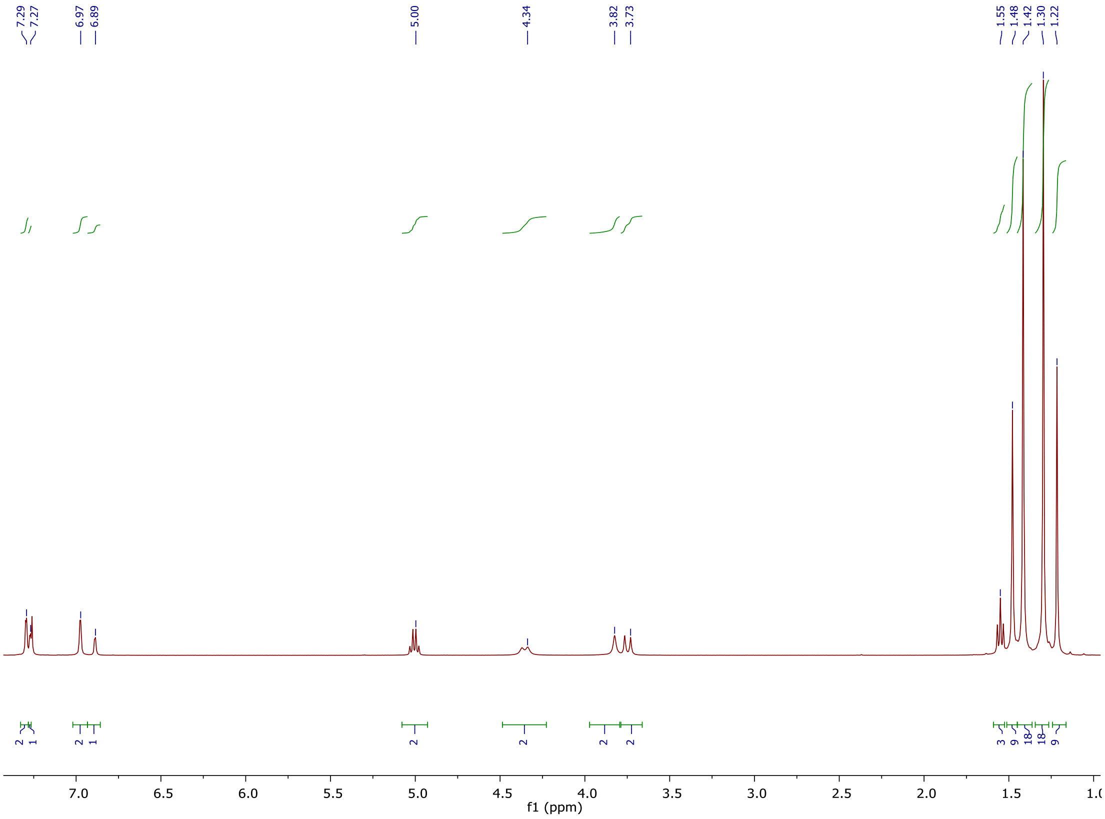

Figure S5. ^{1}H NMR spectrum of **2b** at 298 K in $CDCl_3$

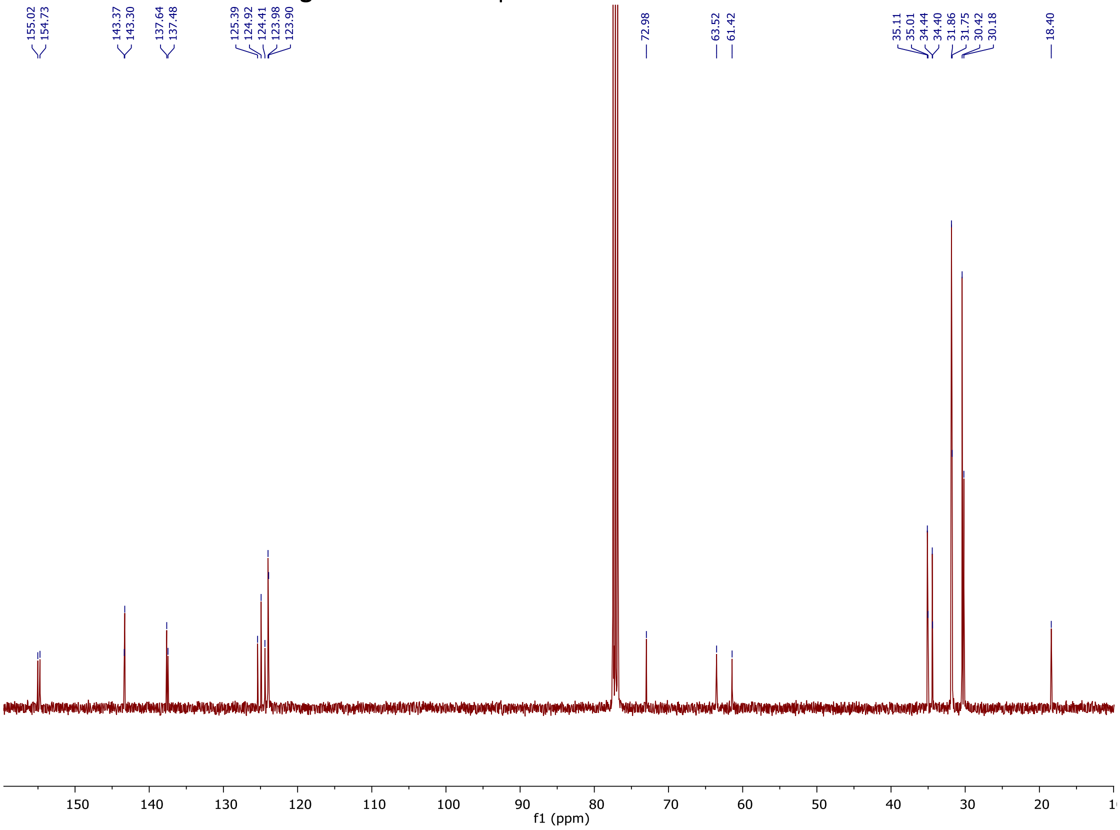

Figure S6. $^{13}C\{^{1}H\}$ NMR spectrum of **2b** at 298 K in $CDCl_3$

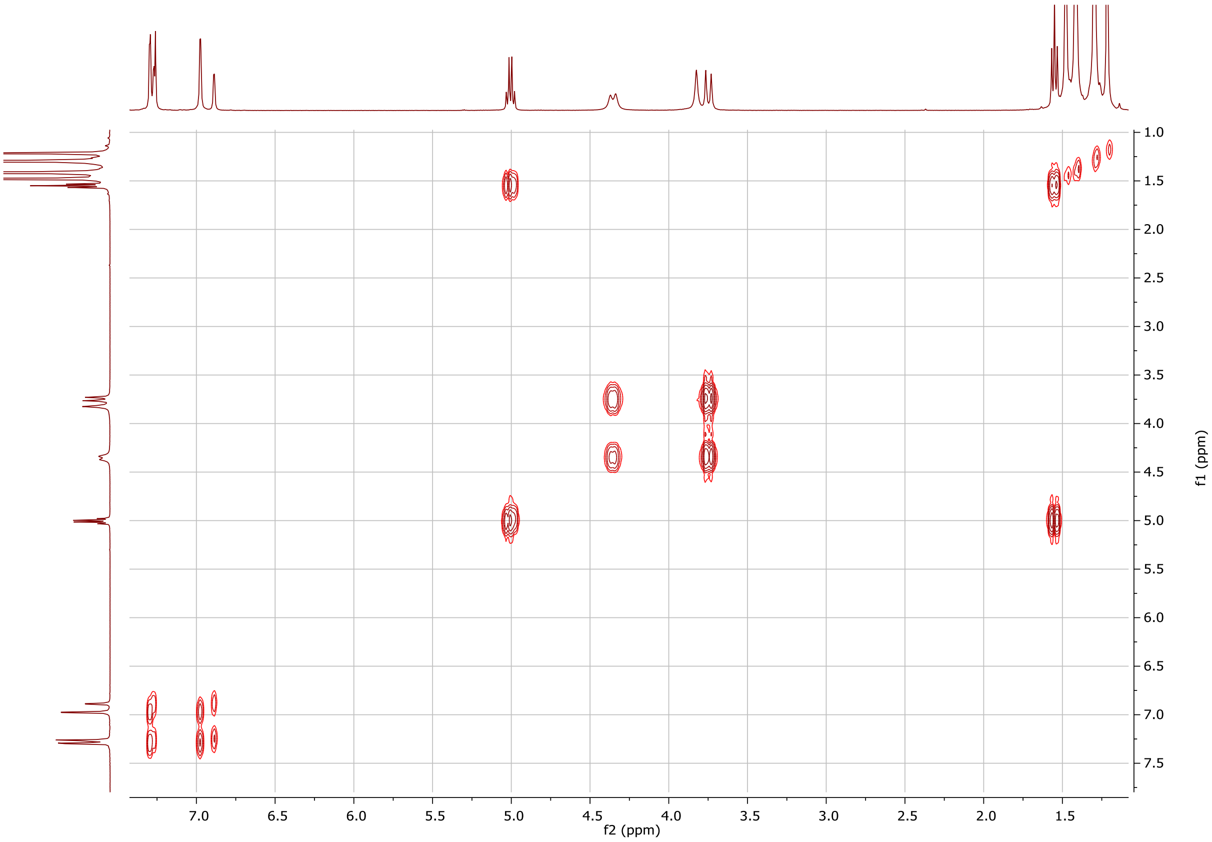

Figure S7. COSY NMR spectrum of **2b** at 298 K in $CDCl_3$

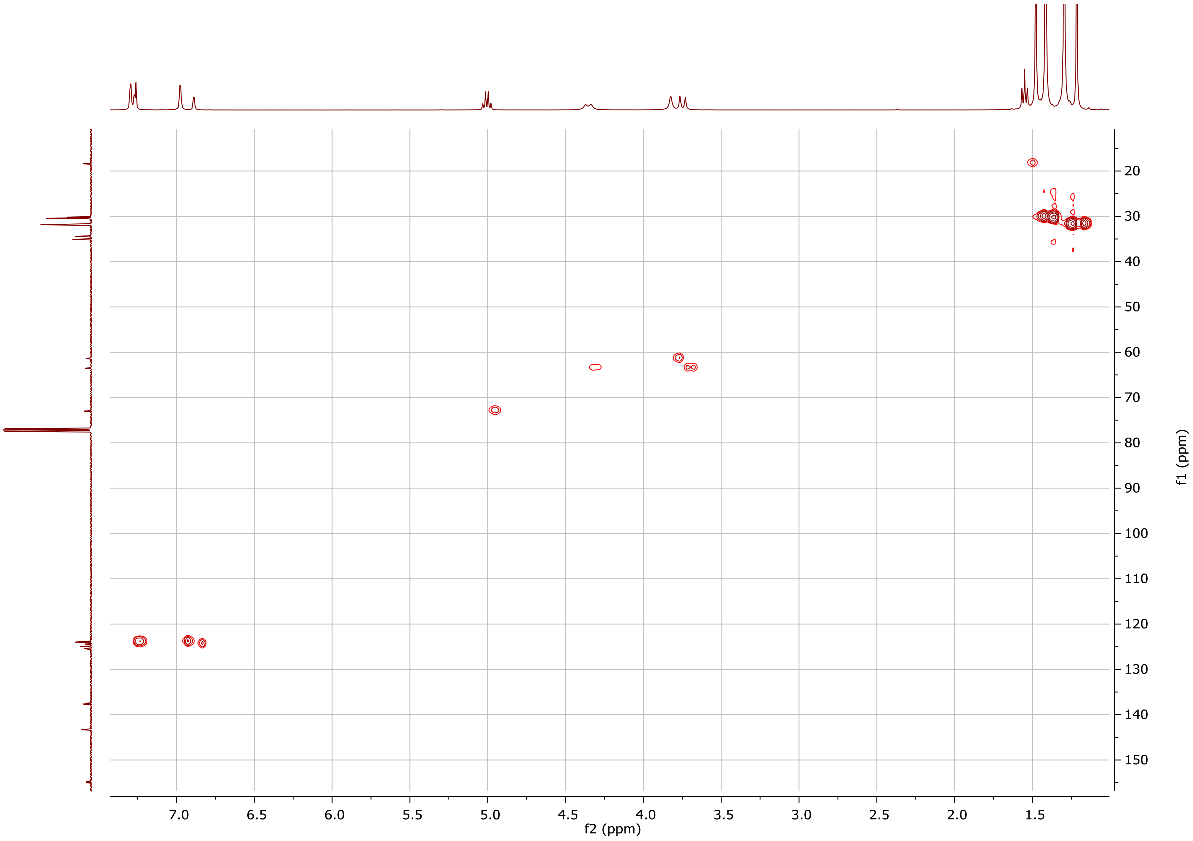

Figure S8. HSQC spectrum of **2b** at 298 K in $CDCl_3$

Synthesis of niobium species [{L^{tBu}NbF}-μ_2F-{L^{tBu}Nb(OEt)}]$^+$[SbF_6]$^-$, 3a

3a was prepared according to literature procedure, and all characterisation data was consistent with literature values.[3]

Synthesis of niobium species [L^{tBu}Nb(OEt)(ε-CL)]$^+$[SbF_6]$^-$, 4a

4a was prepared according to literature procedure, and all characterisation data was consistent with literature values.[3]

Synthesis of tantalum species [L^{tBu}Ta(OEt)(ε-CL)]$^+$[SbF_6]$^-$, 4b

To a solution of **2b** (2.15 mmol, 2.00 g) and ε-CL (9.90 mmol, 1.13 g) in toluene (20 ml) in a vial in the glove box at ambient temperature, was added $AgSbF_6$ (3.29 mmol, 1.13 g). The mixture was then shaken vigorously for 30 seconds, and syringe filtered through a 0.2 µm PTFE filter to remove the AgCl by-product. Then, hexane was added (10 ml), and the product allowed to crystallise for 16 hours, the solvent then being removed by syringe and needle. Extensive purification steps were required to minimise contamination with uncoordinated ε-CL: The solid product was washed three times with a mixture containing equal volumes of toluene and hexane (3x20 ml), which was then removed each time using a syringe and needle. The solid product was then dissolved in DCM, and syringe filtered into a Schlenk, and dried briefly under dynamic vacuum. The solid material was then washed again with toluene and hexane (4x 50 ml) and then with pentane (1x50 ml), before drying briefly under dynamic vacuum. The solid was again dissolved in DCM, and precipitated from toluene and hexane. The solvent was decanted, and the solid washed with toluene and hexane (2x50 ml) and dried briefly under dynamic vacuum. The solid was dissolved a final time in DCM, cannula filtered, and the solvent removed under dynamic vacuum. Crystals suitable for diffraction were obtained by an identical procedure, but without the purification steps described above.

Yield: 1.27 g, 46%

^{1}H NMR (400 MHz, $CDCl_3$, 298 K, δ_H, ppm); 7.40 (1H, *d*, *J* = 1.73 Hz, Ar*H*), 7.35 (2H, *d*, *J* = 1.60 Hz, Ar*H*), 7.14 (3H, *broad*, Ar*H*), 5.02 (2H, *q*, *J* = 7.11 Hz, OCH_2), 4.43 (2H, *s*, *broad*, Ta-ε-CL OCH_2), 4.28 (<1H uncoordinated ε-CL OCH_2)*, 4.07 (<1H, *obscured by* NCH_2 *signals*, PCL OCH_2)**, 3.60-4.24 (6H, *broad*, NCH_2), 2.66 (<1H, uncoordinated ε-CL C(O)CH_2)*, 2.32 (<1H, *obscured by toluene signal*, PCL CH_2), 2.12 (2H, *broad*, Ta-ε-CL CH_2), 1.86 (<1H, uncoordinated ε-CL CH_2)*, 1.76 (<1H, uncoordinated ε-CL CH_2)*, 1.68 (<1H, PCL CH_2)**, 1.58 (3H, *t*, *J* = 7.10 Hz, OCH_2CH_3), 1.52 (11H, *s*, $C(CH_3)_3$ *and* Ta-ε-CL CH_2), 1.39 (2H, *t*, *partially obscured by* $C(CH_3)_3$ *signal*, *J* = 7.14 Hz, Ta-ε-CL OCH_2CH_2), 1.36 (18H, *s*, $C(CH_3)_3$), 1.28 (27H, *s*, $C(CH_3)_3$). **^{1}H NMR** (400 MHz, $CDCl_3$, 233 K, δ_H, ppm); 7.00-7.40 (6H, Ar*H*), 4.97 (2H, *q*, *J* = 6.77 Hz, OCH_2), 4.51 (3H, *d*, *broad*, *J* = 12.49 Hz, 2NC*H*H *and* Ta-ε-CL OC*H*H), 4.41 (1H, *broad*, Ta-ε-CL OC*H*H), 4.31 (<1H, uncoordinated ε-CL OCH_2)*, 4.11 (1H, *d*, *J* = 12.67 Hz, NCH*H*), 4.03 (<1H, PCL OCH_2)**, 3.66 (1H, *d*, *J* = 16.10 Hz, NCH*H*), 3.44 (1H, *d*, *J* = 13.18 Hz, NCH*H*), 3.32 (1H, *d*, *J* = 13.43 Hz, NCH*H*), 2.66 (<1H, uncoordinated ε-CL C(O)CH_2)*, 2.52 (1H, *s*, *broad*, Ta-ε-CL CH*H*), 2.33 (<1H, PCL CH_2)**, 1.84 (<1H uncoordinated ε-CL CH_2)*, 1.61-1.78 (2H, *broad*, Ta-ε-CL CH*H*, *and* uncoordinated ε-CL CH_2,* *and* PCL CH_2**), 1.57 (3H, *t*, *J* = 6.94, OCH_2CH_3), 1.49 (11H, *s*, *broad*, Ta-ε-CL CH_2 *and* $C(CH_3)_3$), 1.34 (11H, *s*, *broad*, Ta-ε-CL CH_2 *and* $C(CH_3)_3$), 1.24 (27H, *s*, $C(CH_3)_3$), 1.22 (9H, *s*, $C(CH_3)_3$). **^{13}C NMR** (101 MHz, $CDCl_3$, 298 K, δ_C, ppm); 187.9 (Nb-ε-CL O=*C*), 155.3 (*Ar*O), 154.7 (*Ar*O), 146.1 (*Ar*), 145.7 (*Ar*), 143.2 (*Ar*), 138.2 (*Ar*), 138.1 (*Ar*), 129.2 (*Ar*), 125.3 (*Ar*H), 125.2 (*Ar*H), 124.8 (*Ar*H), 124.7 (*Ar*H), 124.6 (*Ar*H), 124.5 (*Ar*H), 75.9 (Nb-ε-CL OCH_2), 74.1 (OCH_2CH_3), 61.8 (NCH_2), 60.4 (NCH_2), 58.4 (NCH_2), 35.1 ($C(CH_3)_3$), 35.1 ($C(CH_3)_3$), 34.7 ($C(CH_3)_3$), 34.7 ($C(CH_3)_3$), 34.3 (Ta-ε-CL CH_2) 31.7 ($C(CH_3)_3$), 30.0 ($C(CH_3)_3$), 30.0 ($C(CH_3)_3$), 27.4 ($C(CH_3)_3$), 27.1 ($C(CH_3)_3$), 18.7 (OCH_2CH_3).

Elemental (CHN) Analysis (Calculated, for $C_{53}H_{81}NO_6F_6SbTa$); C: 51.13 %, H: 6.56 %, N: 1.13 %, (Calculated, for $C_{47}H_{71}NO_4F_6SbTa$); C: 49.92 %, H: 6.33 %, N: 1.24 %, (Experimental); C: 50.06 %, H: 6.42 %, N: 1.22 %.***

ESI-MS (m/z): 1008.5336; calc. for $[C_{53}H_{81}NO_6Ta]^+$ (***4b***): 1008.5538.

(m/z): 894.4891; calc. for $[C_{47}H_{71}NO_4Ta]^+$ (***4b** after loss of ε-CL*): 894.4858.

* *Residual ε-CL that was not removed by repeated washing with apolar solvents (toluene and hexane). ~0.20 equivalents.*

** *PCL formed in-situ in $CDCl_3$ solution via ROP of residual ε-CL. The absence of any significant ethoxy end group signal in the ^1H NMR spectrum indicates a negligible fraction of the sample of **4b** underwent initiation to produce this contaminant. ~0.15 equivalents.*

*** *Elemental Analysis data was consistently compatible with the species $[L^{tBu}Ta(OEt)]^+[SbF_6]^-$ indicating quantitative removal of coordinated ε-CL had occurred during sample preparation (prolonged vacuum drying). High resolution mass spectrometry, for which samples were prepared in wet methanol, under ambient air, contained peaks corresponding to both $[L^{tBu}Ta(OEt)]^+$ and $[L^{tBu}Ta(OEt)(ε\text{-}CL)]^+$. However, ^1H and ^{13}C NMR analysis unambiguously shows $[L^{tBu}Ta(OEt)(ε\text{-}CL)]^+[SbF_6]^-$ to be the only complex present in our bulk samples of **4b** (used for catalysis). The absence of $[L^{tBu}Ta(OEt)]^+[SbF_6]^-$ (i.e. retention of coordinated ε-CL) in bulk samples of **4b** is also confirmed by the inactivity of **4b** in the ROP of β-BL at 25 °C, whereas **3a** is active (see main paper). The presence of inorganic impurities was also discounted because samples of **4b** were consistently fully soluble in DCM, $CDCl_3$ and, on heating during catalytic use, in toluene (and toluene-d_8), yielding colourless solutions.*

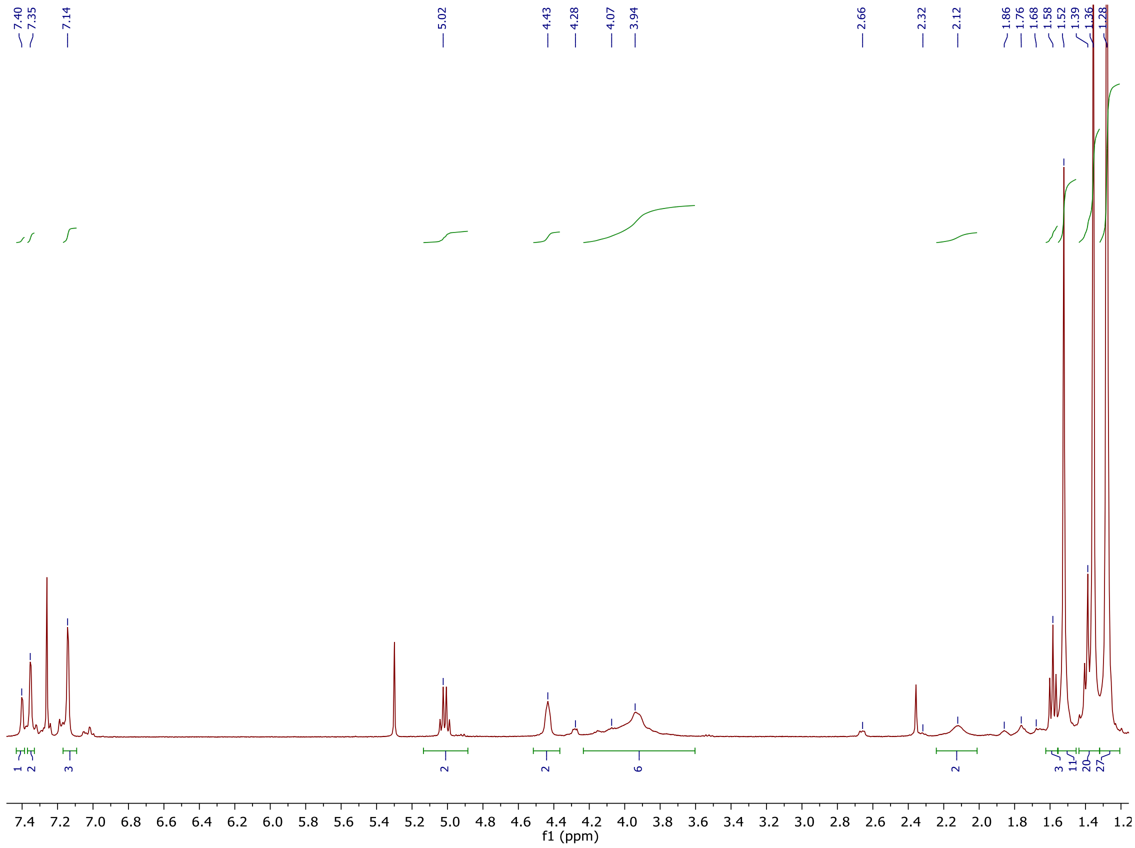

Figure S9. ^1H NMR spectrum of **4b** at 298 K in $CDCl_3$

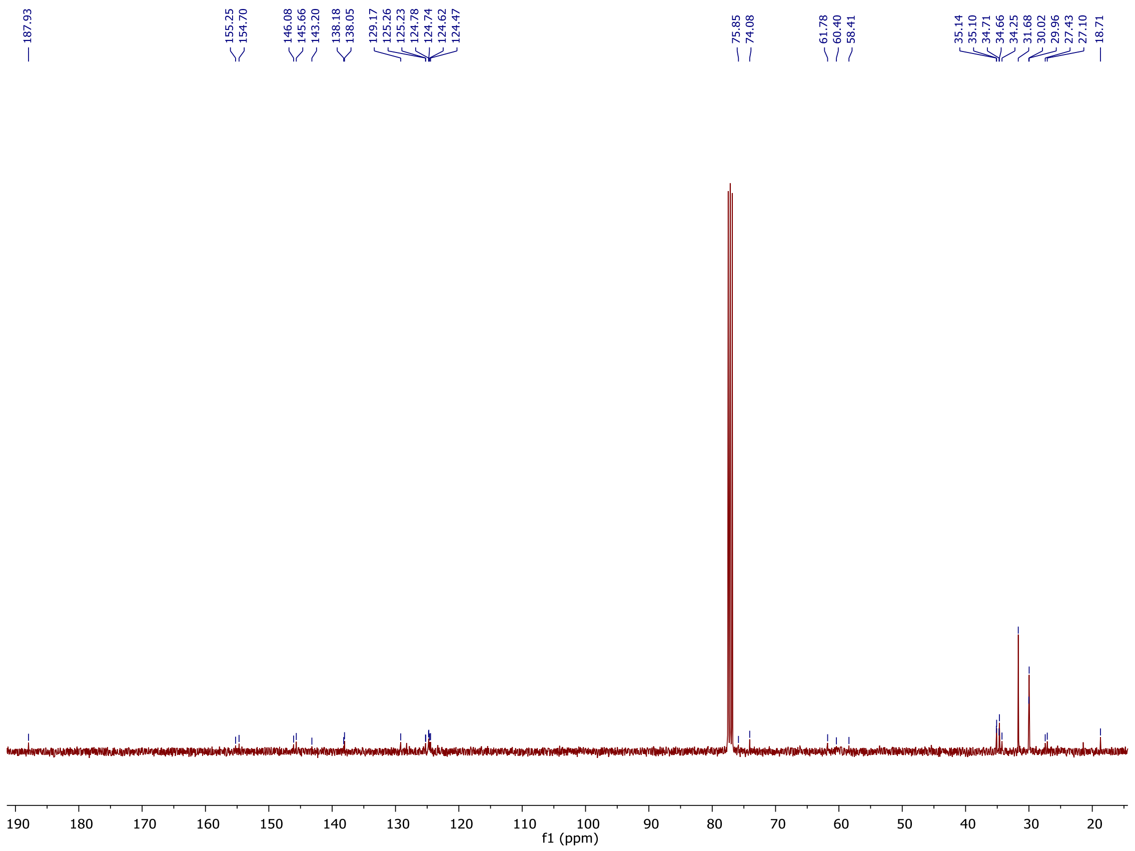

Figure S10. $^{13}C\{^{1}H\}$ NMR spectrum of **4b** at 298 K in $CDCl_3$

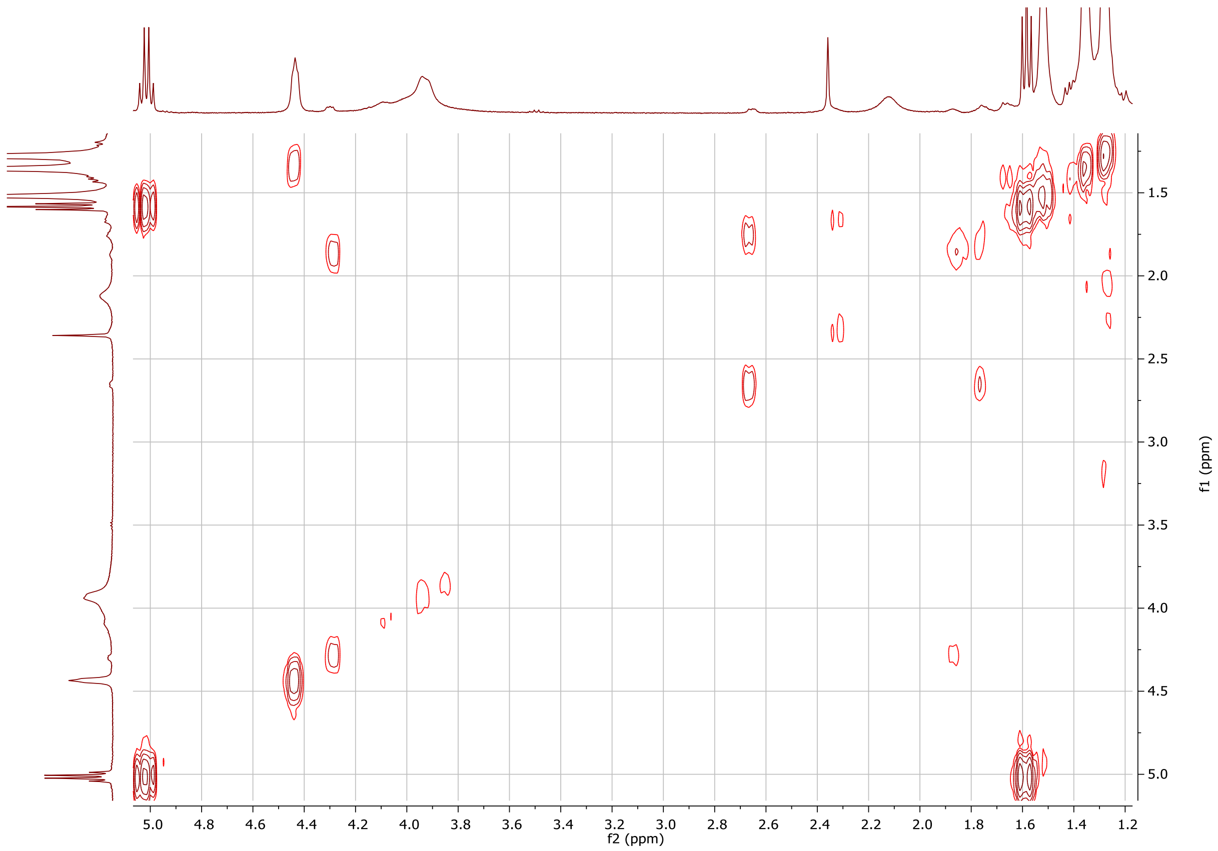

Figure S11. COSYNMR spectrum of **4b** at 298 K in $CDCl_3$

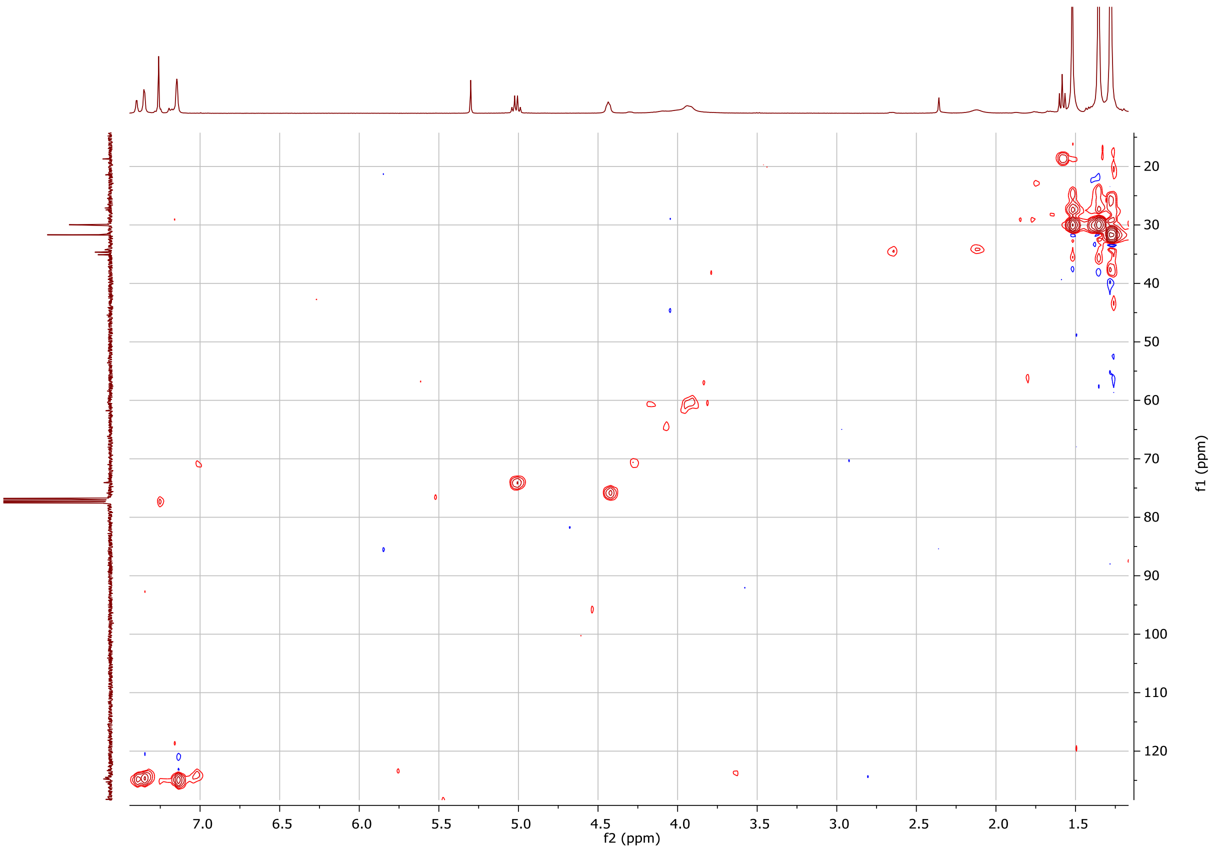

Figure S12. HSQC NMR spectrum of **4b** at 298 K in $CDCl_3$

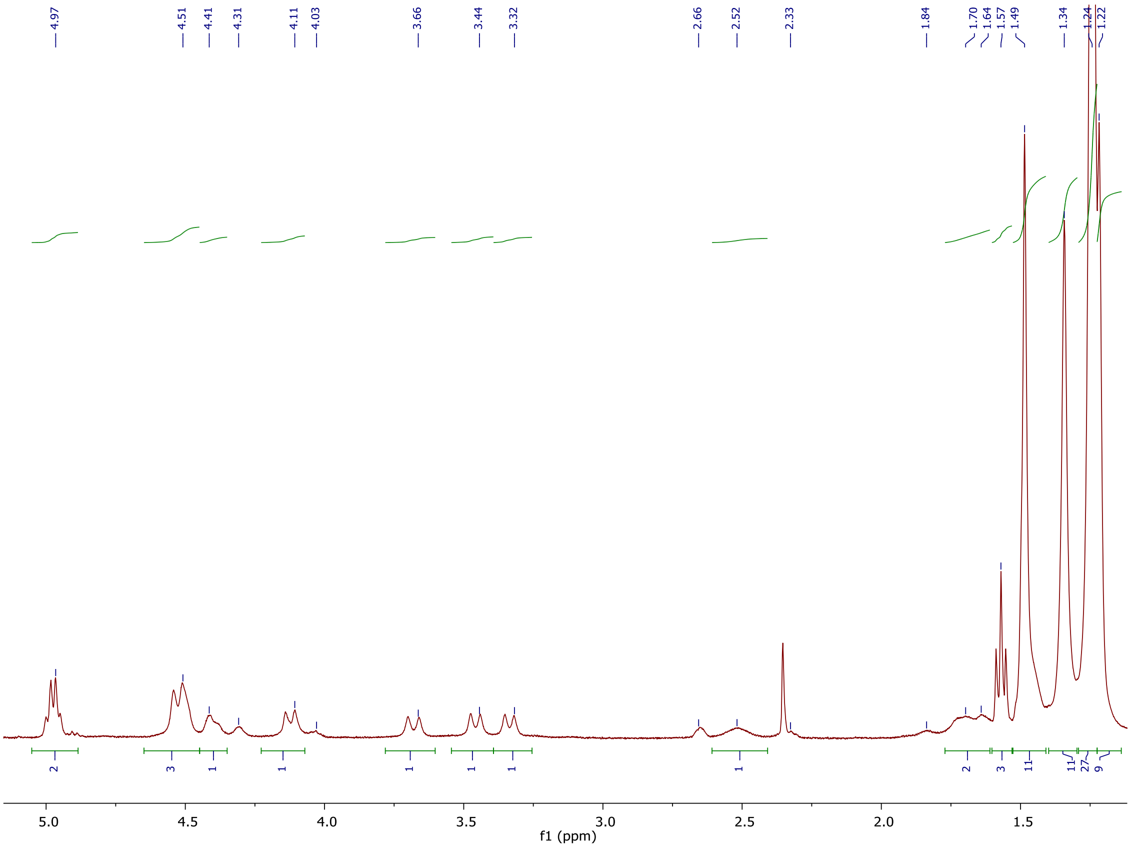

Figure S13. ^{1}H NMR spectrum of **4b** at 233 K in $CDCl_3$

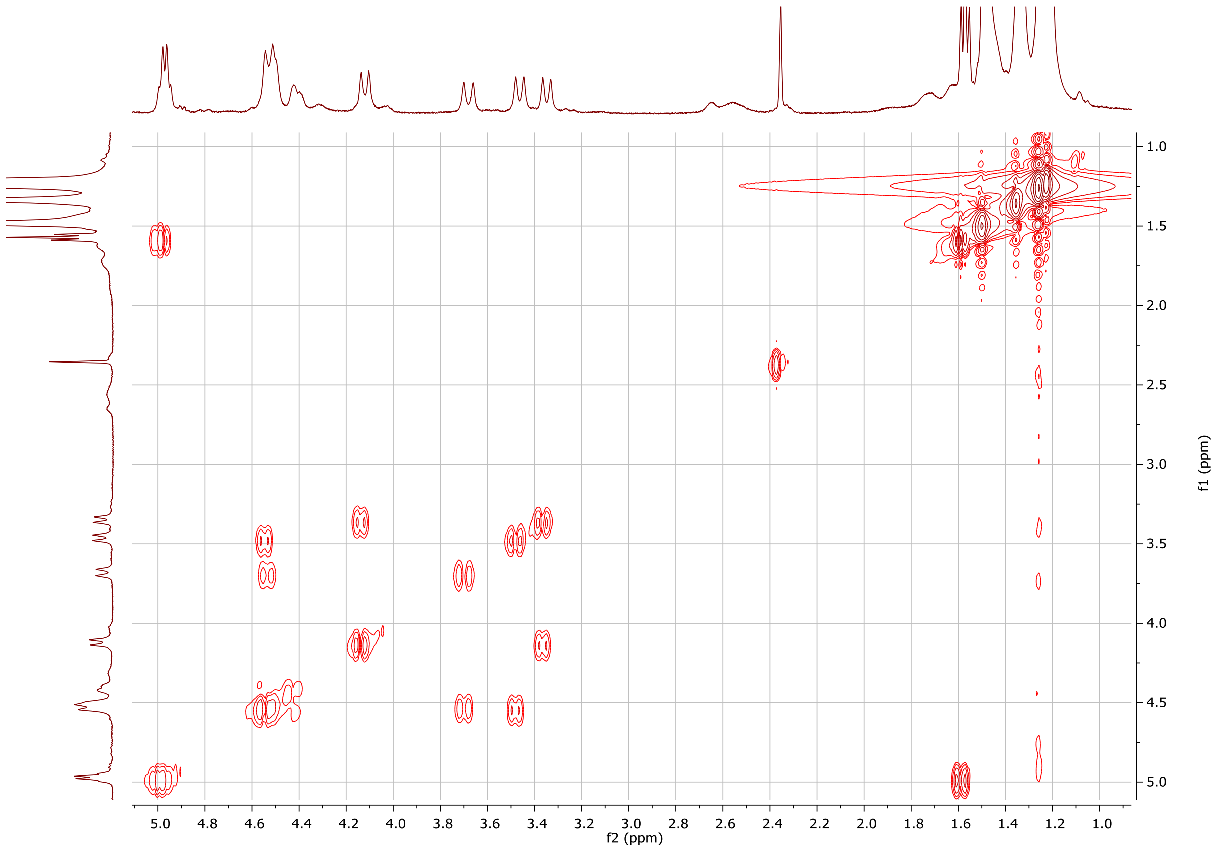

Figure S14. COSY NMR spectrum of **4b** at 233 K in $CDCl_3$

Synthesis of niobium species $[L^{tBu}Nb(OEt)(\delta\text{-VL})]^+[SbF_6]^-$, 5a

To a solution of **2a** (0.77 mmol, 600 mg) and δ-VL (2.00 mmol, 200 mg) in toluene (6 ml) in a vial in the glove box at ambient temperature, was added $AgSbF_6$ (1.07 mmol, 366 mg). The mixture was then shaken vigorously for 30 seconds, and syringe filtered through a 0.2 µm PTFE filter to remove the AgCl by-product. The solution was then allowed to stand for 2 hours, after which crystallisation of **5a** had occurred. The solvent was removed by decantation, and the solid material washed with toluene (2x10 ml), which was removed by syringe and needle. The solid was then dissolved in DCM (~2 ml) and precipitated by addition of hexane (~10 ml). **5a** was then isolated by decantation and dried briefly under dynamic vacuum. Crystals suitable for diffraction were obtained by an analogous procedure, without washing and recrystallization/ precipitation steps.

Yield: 353 mg, 40%

^{1}**H NMR** (500 MHz, $CDCl_3$, 298 K, δ_H, ppm); 7.35 (1H, *s*, Ar*H*), 7.31 (2H, *s*, Ar*H*), 7.20 (2H, *s*, Ar*H*), 7.17 (1H, *s*, Ar*H*), 4.97 (2H, *q*, *J* = 7.05 Hz, OC*H*$_2$), 4.45 (1H, *broad*, uncoordinated δ-VL OC*H*$_2$)*, 4.40 (2H, *t*, *broad*, *J* = 5.61 Hz, Nb-δ-VL OC*H*$_2$), 4.09 (<1H, *obscured by NCH$_2$*, PVL OC*H*$_2$)**, 3.85 (6H, *broad*, NC*H*$_2$), 2.57, (1H, *broad*, uncoordinated δ-VL C*H*$_2$)*, 2.36 (<1H, *obscured by toluene CH$_3$ signal*, PVL C*H*$_2$)** 1.94 (2H, *t*, *broad*, *J* = 6.68 Hz, Nb-δ-VL C*H*$_2$), 1.89 (2H, *broad*, uncoordinated δ-VL 2C*H*$_2$)*, 1.69 (<1H, *broad*, PVL C*H*$_2$)**, 1.62 (3H, *t*, *J* = 7.00 Hz, OCH_2CH_3), 1.60 (2H, *broad*, Nb-δ-VL C*H*$_2$), 1.55 (2H, *t*, *broad*, *obscured by tert-butyl signals, J* = 5.98 Hz, Nb-δ-VL C*H*$_2$), 1.52 (9H, *s*, C(C*H*$_3$)$_3$), 1.36 (18H, *s*, C(C*H*$_3$)$_3$), 1.29 (18H, *s*, C(C*H*$_3$)$_3$), 1.28 (9H, *s*, C(C*H*$_3$)$_3$). ^{1}**H NMR** (400 MHz, $CDCl_3$, 233 K, δ_H, ppm); 7.32 (1H, *d*, *J* = 1.89 Hz, Ar*H*), 7.29 (1H, *broad*, Ar*H*), 7.21 (2H, *broad*, Ar*H*), 7.16 (1H, *d*, *J* = 1.89 Hz, Ar*H*), 7.09 (1H, *broad*, Ar*H*), 4.94 (2H, *m*, OC*H*$_2$CH$_3$), 4.55 (1H, *d*, *J* = 15.88 Hz, NC*H*H), 4.48 (1H, *s*, *obscured by uncoordinated δ-VL*, Nb-δ-VL OCH*H*), 4.47 (1H, *s*, uncoordinated Nb-δ-VL OC*H*$_2$)*, 4.38 (2H, *d*, *broad*, *J*

= 13.30 Hz, NCH*H and* Nb-δ-VL OCH*H*), 4.06 (<1H, PVL OC*H₂*)**, 3.98 (1H, *d*, *J* = 13.50 Hz, NCH*H*), 3.60 (1H, *d*, *J* = 15.88 Hz, NCH*H*), 3.39 (1H, *d*, *J* = 13.90 Hz, NCH*H*), 3.30 (1H, *d*, *J* = 13.30 Hz, NCH*H*), 2.56 (1H, uncoordinated δ-VL C(O)CH_2)*, 2.36 (<1H, PVL C(O)CH_2), 1.97 (1H, *broad*, Nb-δ-VL CH*H*), 1.89 (2H, uncoordinated δ-VL CH_2)*, 1.84 (1H, *broad*, Nb-δ-VL CH*H*), 1.73 (1H, *broad*, Nb-δ-VL CH*H*), 1.67 (1H, PVL CH_2)**, 1.62 (3H, *t*, *J* = 7.01 Hz, OCH_2CH_3), 1.49 (10H, *s*, C(CH_3)$_3$ *and* Nb-δ-VL CH*H*), 1.40 (1H, *broad*, Nb-δ-VL CH*H*), 1.32 (9H, *s*, C(CH_3)$_3$), 1.28 (9H, *s*, C(CH_3)$_3$), 1.25 (27H, *s*, C(CH_3)$_3$). **^{13}C NMR** (101 MHz, $CDCl_3$, 298 K, δ_C, ppm); 183.4 (Nb-δ-VL O=*C*), 156.4 (*Ar*O), 146.3 (*Ar*), 145.9 (*Ar*), 137.2 (*Ar*), 137.0 (*Ar*), 129.1 (*Ar*), 127.8 (*Ar*), 125.4 (*Ar*H), 125.0 (*Ar*H), 124.8 (*Ar*H), 124.4 (*Ar*H), 124.0 (*Ar*H), 123.9 (*Ar*H), 76.8 (O*C*H_2CH_3), 75.2 (Nb-δ-VL O*C*H_2), 61.5 (N*C*H_2), 60.3 (N*C*H_2), 35.2 (*C*(CH_3)$_3$), 35.2 (*C*(CH_3)$_3$), 34.8 (*C*(CH_3)$_3$), 31.7 (C(*C*H_3)$_3$), 31.6 (C(*C*H_3)$_3$), 30.0 (C(*C*H_3)$_3$), 29.9 (C(*C*H_3)$_3$), 29.2 (Nb-δ-VL *C*H_2), 21.1 (Nb-δ-VL *C*H_2), 18.6 (OCH_2*C*H_3), 17.1 (Nb-δ-VL *C*H_2).

ESI-MS (m/z): 906.5002; calc. for $[C_{52}H_{79}NO_6Nb]^+$ (***5a***): 906.4966.

(m/z): 806.4483; calc. for $[C_{47}H_{71}NO_4Nb]^+$ (***5a*** *after loss of δ-VL*): 806.4441.

* *Residual δ-VL that was not removed by repeated washing with apolar solvents (toluene and hexane), ~0.35 equivalents.*

** *PVL formed in-situ in $CDCl_3$ solution via slow ROP of residual δ-VL. The absence of any significant ethoxy end group signal in the ^1H NMR spectrum indicates a negligible fraction of the sample of **4b** underwent initiation to produce this contaminant.~0.45 equivalents.*

*Elemental Analysis was not carried out, due to the observed instability of **4b** toward loss of coordinated ε-CL on prolonged exposure to vacuum, and presumed instability of **5a** to loss of δ-VL. ^1H and ^{13}C NMR confirmed the purity of our bulk samples of **5a**, apart from some residual toluene (not removed due to presumed instability to vacuum), uncoordinated δ-VL, and small amounts of PVL (see above). The masses of those impurities were accounted for when carrying out catalytic studies. Both the cationic fragment of **5a**, $[L^{tBu}Nb(OEt)(\delta\text{-}VL)]^+$, and the species $[L^{tBu}Nb(OEt)]^+$, formed on loss of δ-VL from **5a**, were detected via high-resolution mass spectrometry. **5a** was fully soluble in DCM, $CDCl_3$ and, on heating during catalytic use, in toluene (and toluene-d_8), yielding colourless solutions.*

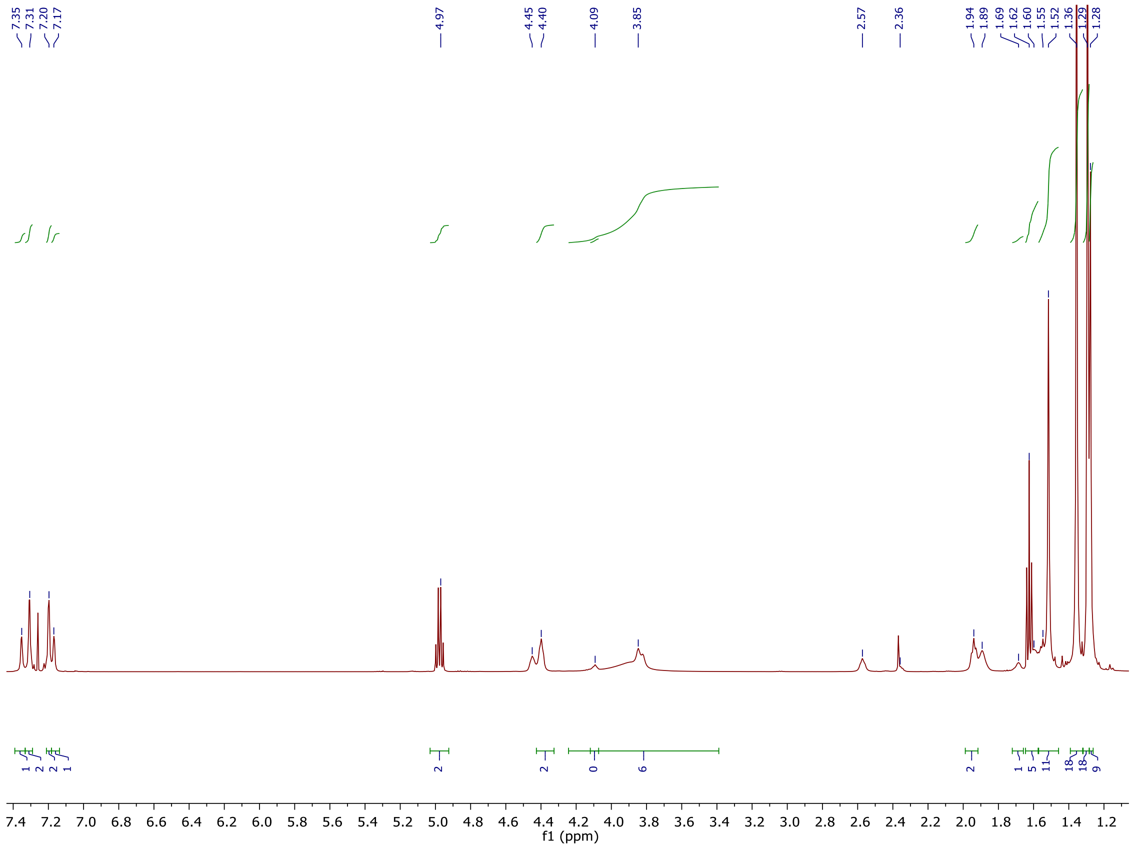

Figure S15. ^{1}H NMR spectrum of **5a** at 298 K in $CDCl_3$

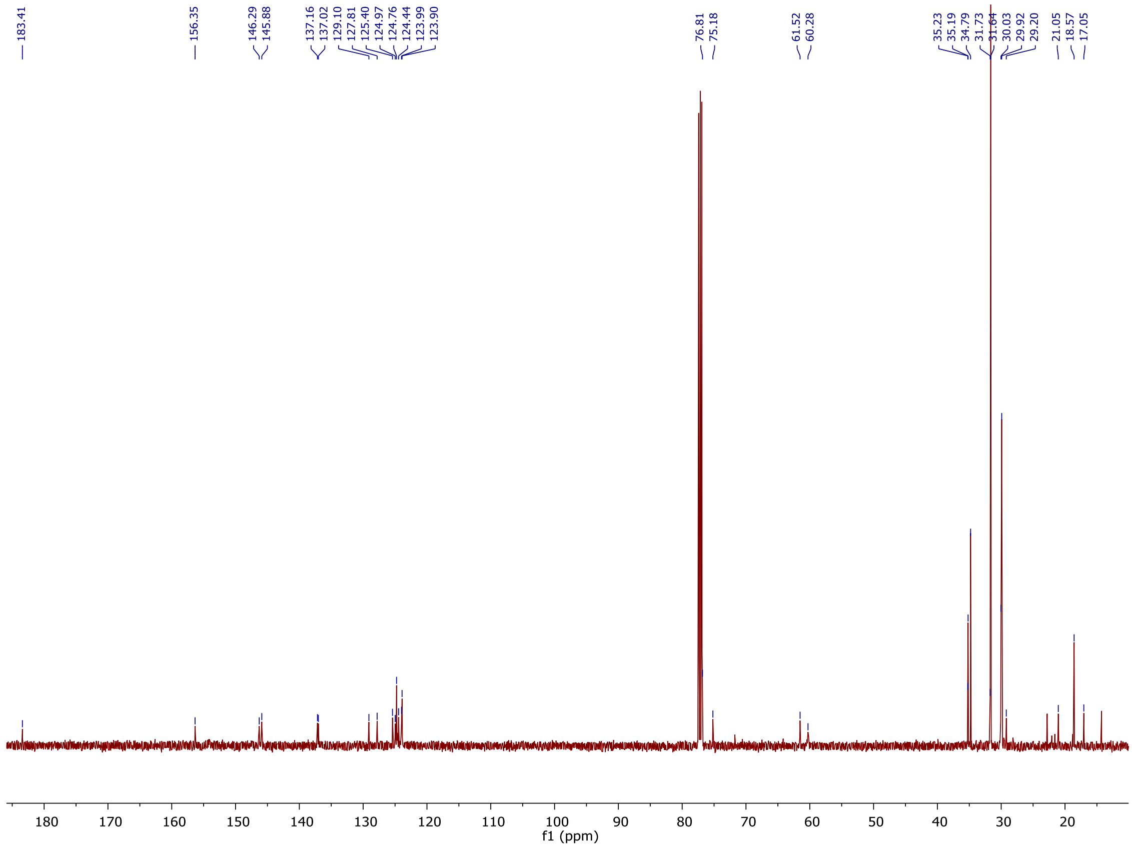

Figure S16. $^{13}C\{^{1}H\}$ NMR spectrum of **5a** at 298 K in $CDCl_3$

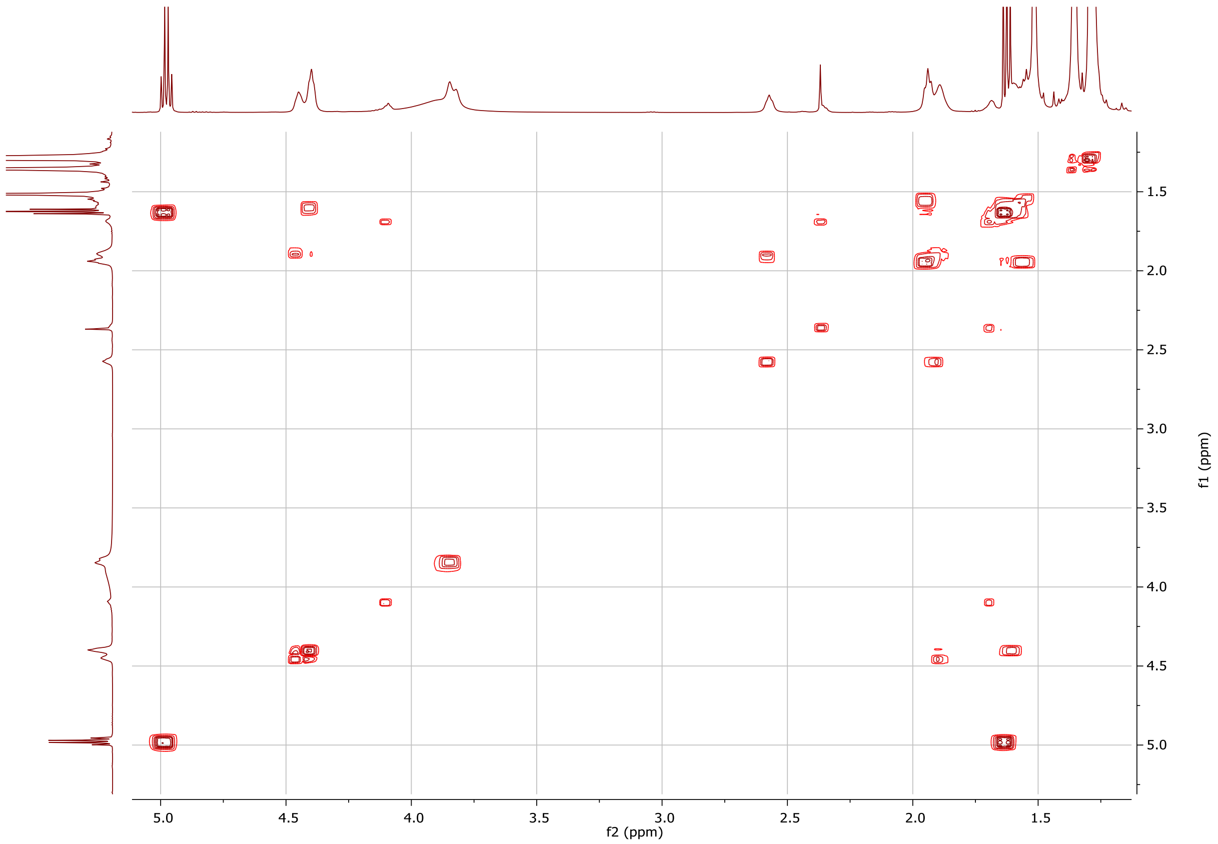

Figure S17. COSY NMR spectrum of **5a** at 298 K in $CDCl_3$

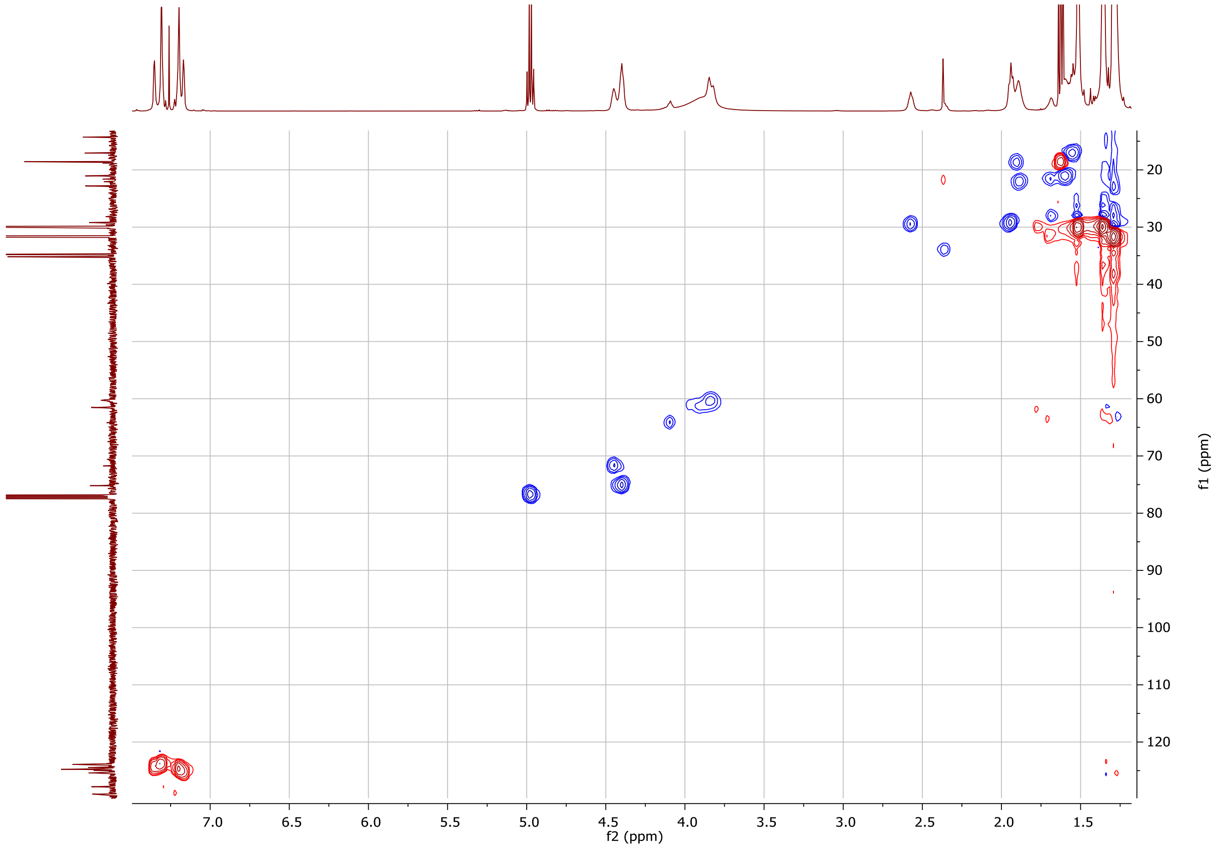

Figure S17. HSQC NMR spectrum of **5a** at 298 K in $CDCl_3$

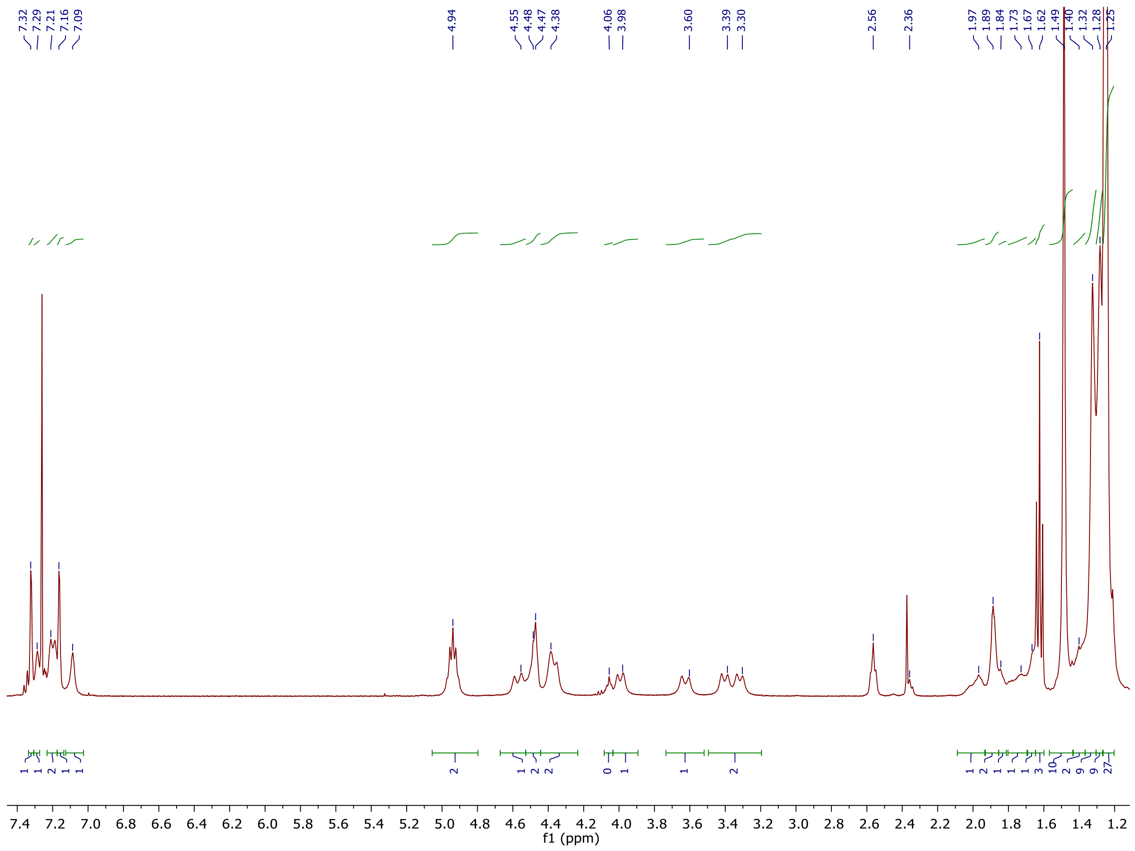

Figure S19. ^{1}H NMR spectrum of **5a** at 233 K in $CDCl_3$

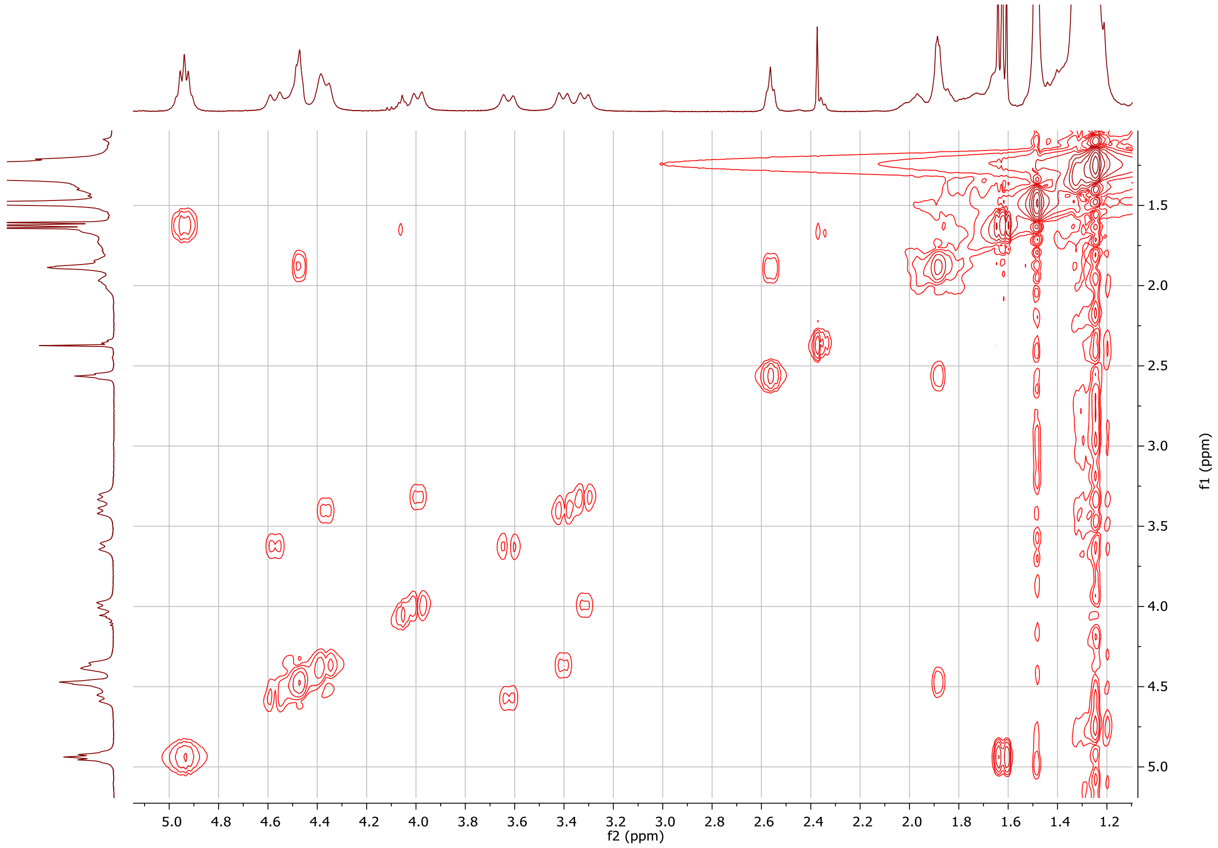

Figure S20. COSY NMR spectrum of **5a** at 233 K in $CDCl_3$

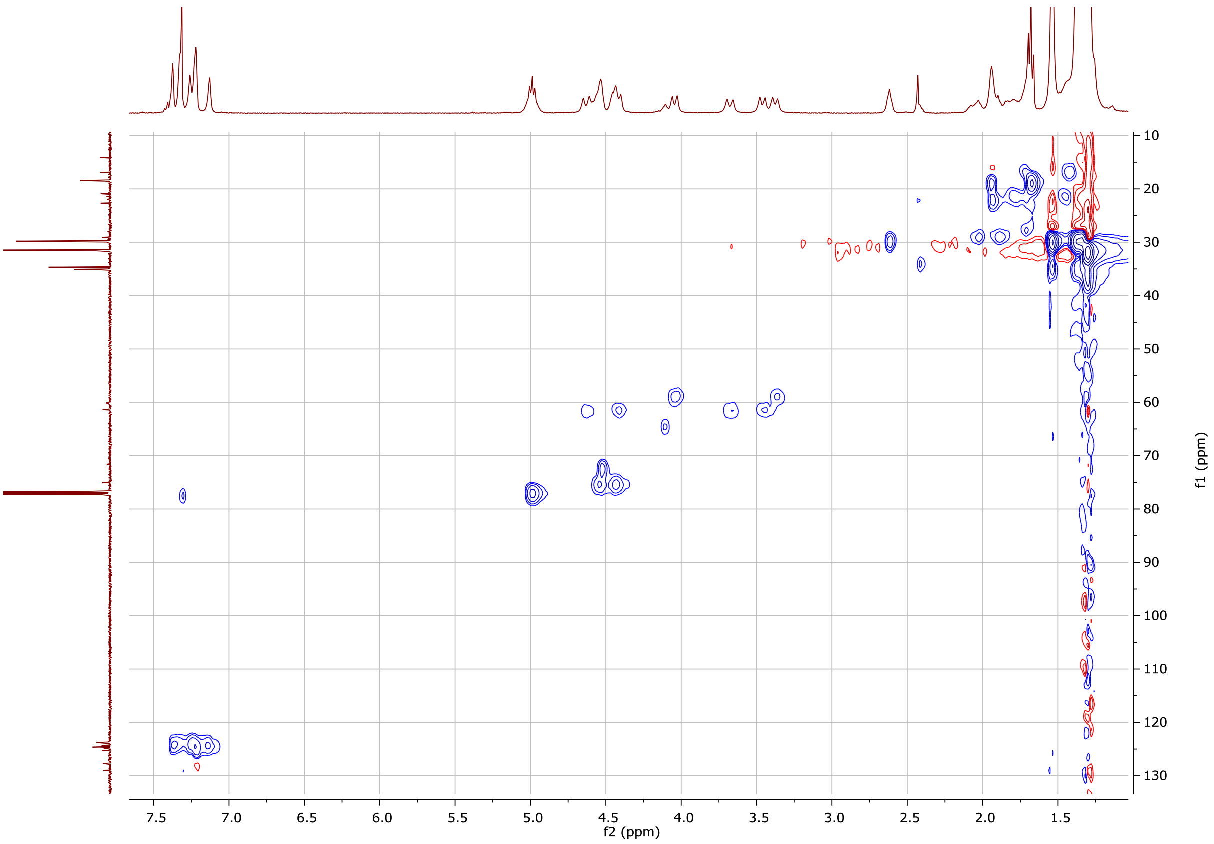

Figure S21. HSQC NMR spectrum of **5a** at 233 K in $CDCl_3$

Synthesis of tantalum species $[L^{tBu}Ta(OEt)(\delta\text{-VL})]^+[SbF_6]^-$, 5b

To a solution of **2b** (0.25 mmol, 230 mg) and δ-VL (0.69 mmol, 69 mg) in toluene (2.3 ml) in a vial in the glove box at ambient temperature, was added $AgSbF_6$ (0.37 mmol, 127 mg). The mixture was then shaken vigorously for 30 seconds, and syringe filtered through a 0.2 µm PTFE filter to remove the AgCl by-product. The solid product, **5b**, immediately crystallised, and was dissolved in DCM (~1 ml) and syringe filtered again. The product was then precipitated by addition of hexane (~6 ml), yielding crystals suitable for diffraction. The solvent was removed by decantation, and the solid material washed twice with hexane (2x10 ml), which was removed by syringe and needle. **5b** was then dried briefly under dynamic vacuum.

Yield: 180 mg, 58 %

^{1}H NMR (500 MHz, $CDCl_3$, 298 K, δ_H, ppm); 7.39 (1H, *d*, *J* = 2.28 Hz, Ar*H*), 7.35 (2H, *d*, *J* = 2.14 Hz, Ar*H*), 7.17 (2H, *d*, *J* = 2.05 Hz, Ar*H*), 7.14 (1H, *d*, *J* = 7.19 Hz, Ar*H*), 5.03 (2H, *q*, *J* = 7.03 Hz, OCH_2), 4.45 (<1H, uncoordinated δ-VL OCH_2)*, 4.43 (2H, *t*, *obscured by uncoordinated* δ-VL, *J* = 5.59 Hz, Ta-δ-VL OCH_2), 4.09 (<1H, *obscured by* NCH_2, PVL OCH_2)**, 3.94 (6H, *broad*, NCH_2), 2.59, (<1H, uncoordinated δ-VL CH_2)*, 2.36 (<1H, *obscured by toluene* CH_3 *signal*, PVL CH_2)** 1.95 (2H, *t*, *J* = 6.66 Hz, Ta-δ-VL CH_2), 1.90 (<2H, *m*, uncoordinated δ-VL 2CH_2)*, 1.69 (<1H, *broad*, PVL CH_2)**, 1.60 (3H, *t*, *J* = 7.03 Hz, OCH_2CH_3), 1.58 (4H, *broad*, *obscured by* $OCH_2\mathbf{CH_3}$ *signals,* Ta-δ-VL CH_2), 1.51 (9H, *s*, $C(CH_3)_3$), 1.36 (18H, *s*, $C(CH_3)_3$), 1.29 (18H, *s*, $C(CH_3)_3$), 1.27 (9H, *s*, $C(CH_3)_3$). **^{1}H NMR** (500 MHz, $CDCl_3$, 233 K, δ_H, ppm); 7.35 (1H, *s*, Ar*H*), 7.32 (1H, *broad*, Ar*H*), 7.24 (1H, *broad*, *obscured by* $CDCl_3$ *signal,* Ar*H*), 7.18 (1H, *broad*, Ar*H*), 7.13 (1H, *s*, Ar*H*), 7.07 (1H, *broad*, Ar*H*), 5.01 (2H, *q*, *broad*, *J* = 6.58 Hz, OCH_2CH_3), 4.58 (1H, d, *J* = 16.11 Hz, NCH*H*), 4.53 (<1H, uncoordinated δ-VL OCH_2), 4.48 (2H, *s*, *obscured by adjacent signals*, Ta-δ-VL OCH_2), 4.41 (1H, *broad*, *obscured by Ta-δ-VL* OCH_2 *signal*, NCH*H*), 4.11 (1H, *d*, *J* = 10.89 Hz, NCH*H*), 4.05 (<1H, PVL OCH_2), 3.68 (1H, *d*, *J* = 14.97 Hz, NCH*H*), 3.44 (1H, *d*, *J* = 12.70 Hz, NCH*H*), 3.35 (1H, *d*, *J* = 12.93 Hz, NCH*H*), 2.59 (<1H, uncoordinated δ-VL C(O)CH_2), 1.97 (1H, *broad*, Ta-δ-VL CH*H*), 1.90 97 (~3H, *s*, *broad*, Ta-δ-VL CH*H and* uncoordinated δ-VL CH_2), 1.74 (1H, *broad*, Ta-δ-VL CH*H*), 1.66 (<1H, PVL CH_2), 1.59 (2H, *t*, *J* = 7.00 Hz, OCH_2CH_3), 1.48 (9H, *s*, $C(CH_3)_3$), 1.33 (9H, *s*, $C(CH_3)_3$), 1.28 (9H, *s*, *obscured by adjacent signal*, $C(CH_3)_3$), 1.25 (27H, *s*, $C(CH_3)_3$). **^{13}C NMR** (101 MHz, $CDCl_3$, 298 K, δ_C, ppm); 184.1 (Ta-δ-VL O=*C*), 154.7 (*Ar*O), 146.0 (*Ar*), 145.6 (*Ar*), 138.0 (*Ar*), 137.9 (*Ar*), 125.2 (*Ar*H), 124.7 (*Ar*H), 124.6 (*Ar*H), 124.5 (*Ar*H), 124.4 (*Ar*H), 123.5 (*Ar*H), 75.8 (Ta-δ-VL C(O)CH_2), 74.1 (OCH_2CH_3), 71.5 (uncoordinated δ-VL C(O)CH_2)*, 61.7 (NCH_2), 60.4 (NCH_2), 35.1 ($C(CH_3)_3$), 34.7 ($C(CH_3)_3$), 31.7 ($C(CH_3)_3$), 30.0 ($C(CH_3)_3$), 29.9 ($C(CH_3)_3$), 29.6 (uncoordinated δ-VL CH_2)*, 29.3 (Ta-δ-VL CH_2), 22.1 (uncoordinated δ-VL CH_2)*, 21.0 (Ta-δ-VL CH_2), 18.8 (uncoordinated δ-VL CH_2)*, 18.7 (OCH_2CH_3), 16.9 (Ta-δ-VL CH_2).

ESI-MS (m/z): 994.5415; calc. for $[C_{52}H_{79}NO_6Ta]^+$ (***5b***): 994.5382.

(m/z): 894.4900; calc. for $[C_{47}H_{71}NO_4Ta]^+$ (***5b*** *after loss of δ-VL*): 894.4858.

* *Residual δ-VL that was not removed by repeated washing with apolar solvents (toluene and hexane). ~0.40 equivalents.*

** *PVL formed in-situ in* $CDCl_3$ *solution via slow ROP of residual δ-VL. The absence of any significant ethoxy end group signal in the* ^{1}*H NMR spectrum indicates a negligible fraction of the sample of* ***5b*** *underwent initiation to produce this contaminant. ~0.20 equivalents.*

Elemental Analysis was not carried out, due to the observed instability of ***4b*** *toward loss of coordinated ε-CL on prolonged exposure to vacuum, and presumed instability of* ***5b*** *to loss of δ-VL.* ^{1}*H and* ^{13}*C NMR confirmed the purity of our bulk samples of* ***5b****, apart from some residual toluene (not removed due to presumed instability to vacuum), uncoordinated δ-VL, and small amounts of PVL (see above). The masses of those impurities were accounted for when carrying out catalytic studies. Both the cationic*

*fragment of **5b**, [L^{tBu}Ta(OEt)(δ-VL)]$^{+}$, and the species [L^{tBu}Ta(OEt)]$^{+}$, formed on loss of δ-VL from **5b**, were detected via high-resolution mass spectrometry. **5b** was fully soluble in DCM, $CDCl_3$ and, on heating during catalytic use, in toluene (and toluene-d_8), yielding colourless solutions.*

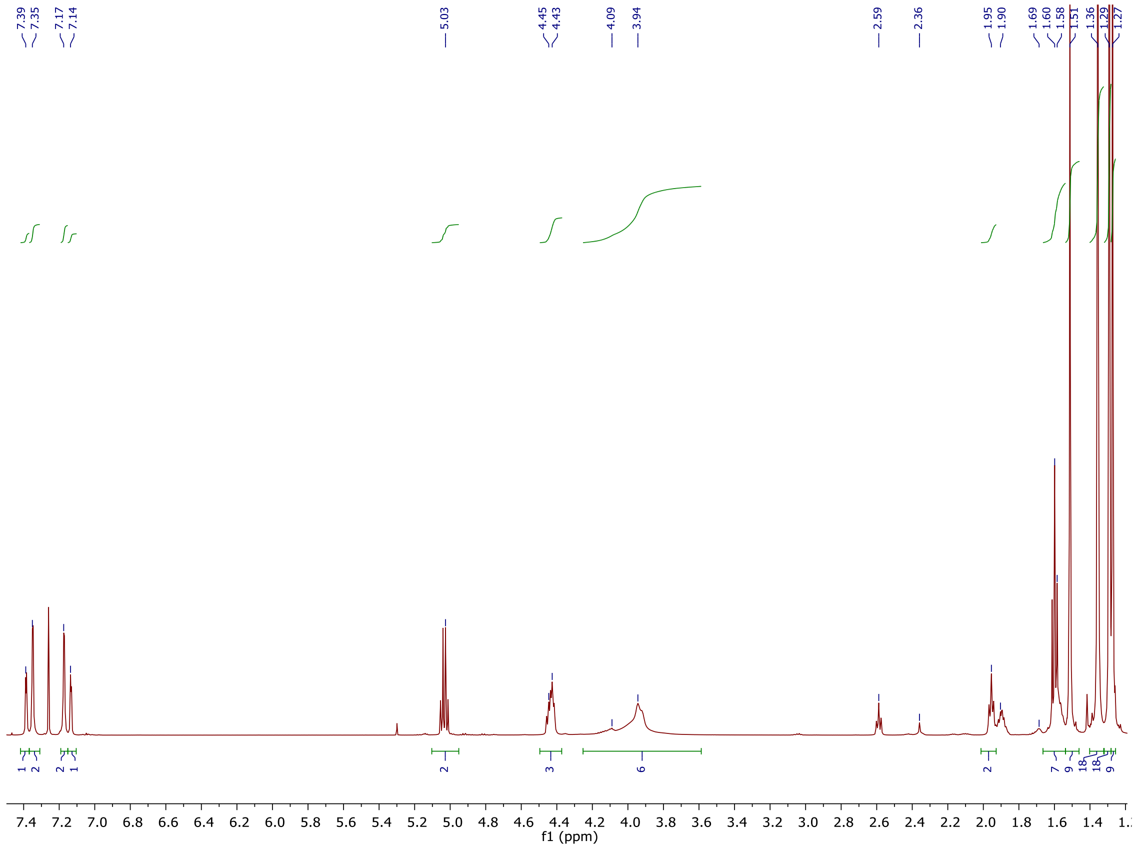

Figure S22. ^{1}H NMR spectrum of **5b** at 298 K in $CDCl_3$

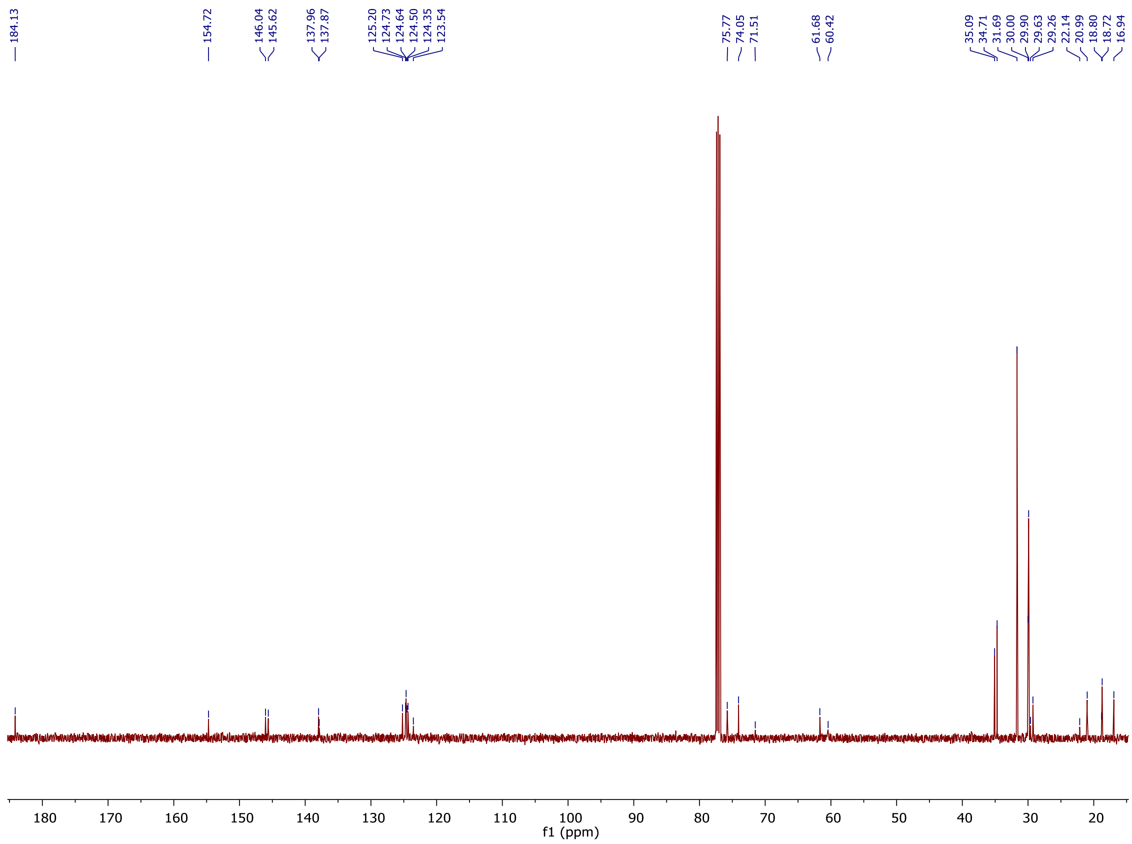

Figure S23. $^{13}C\{^{1}H\}$ NMR spectrum of **5b** at 298 K in $CDCl_3$

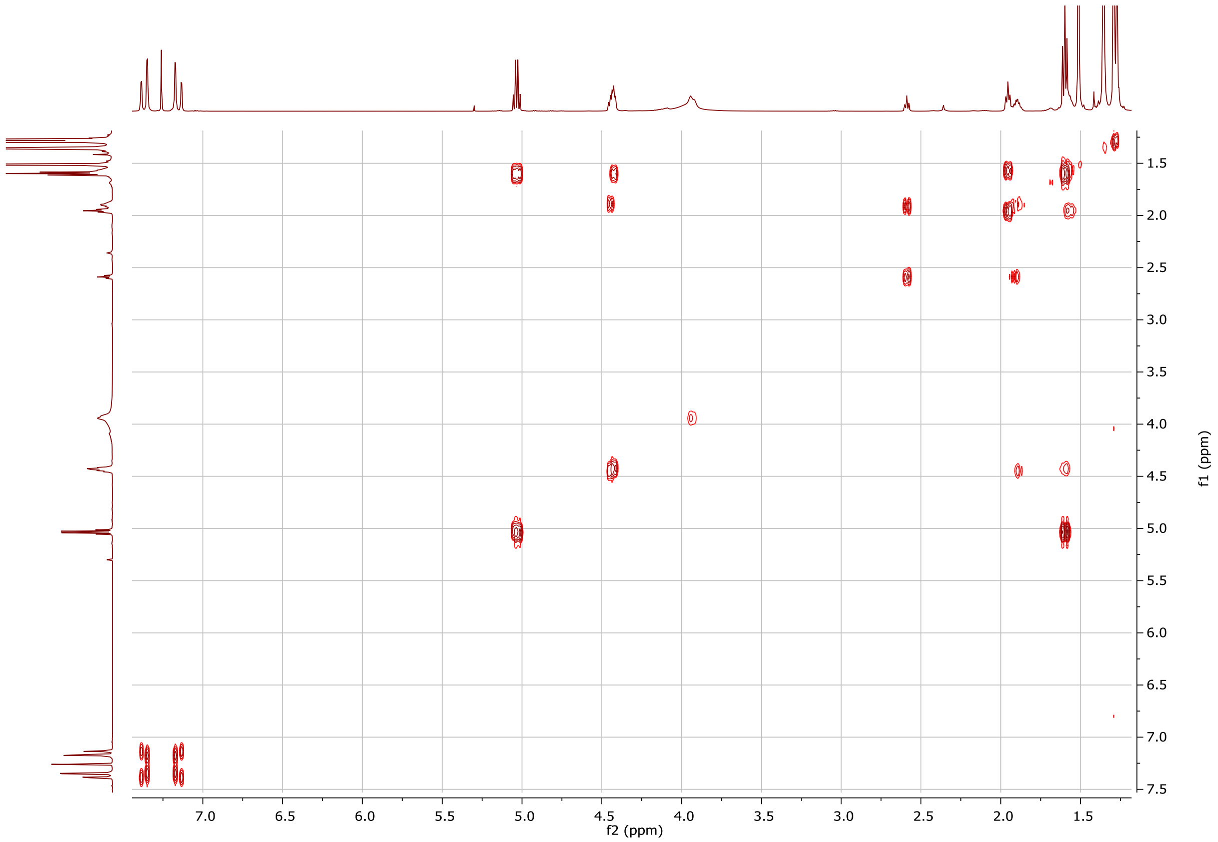

Figure S24. COSY NMR spectrum of **5b** at 298 K in $CDCl_3$

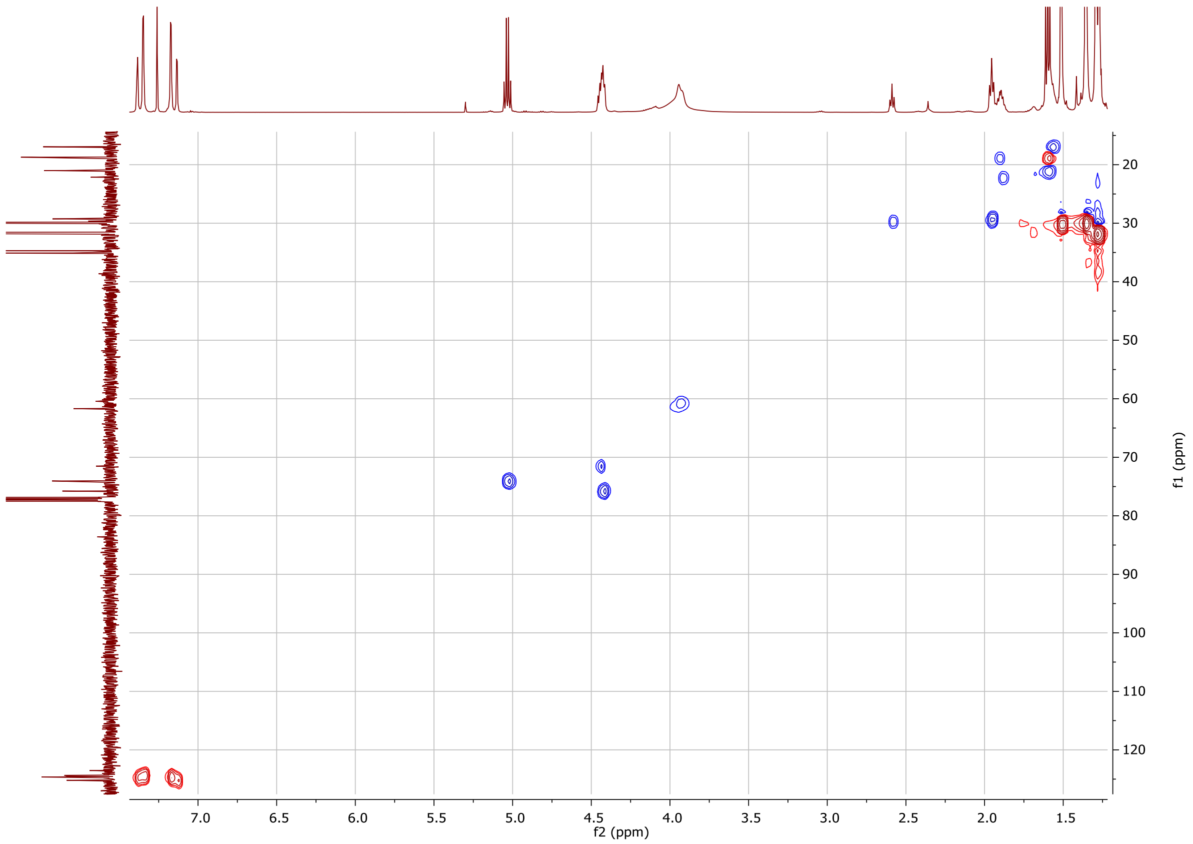

Figure S25. ^{1}H NMR spectrum of **5b** at 298 K in $CDCl_3$

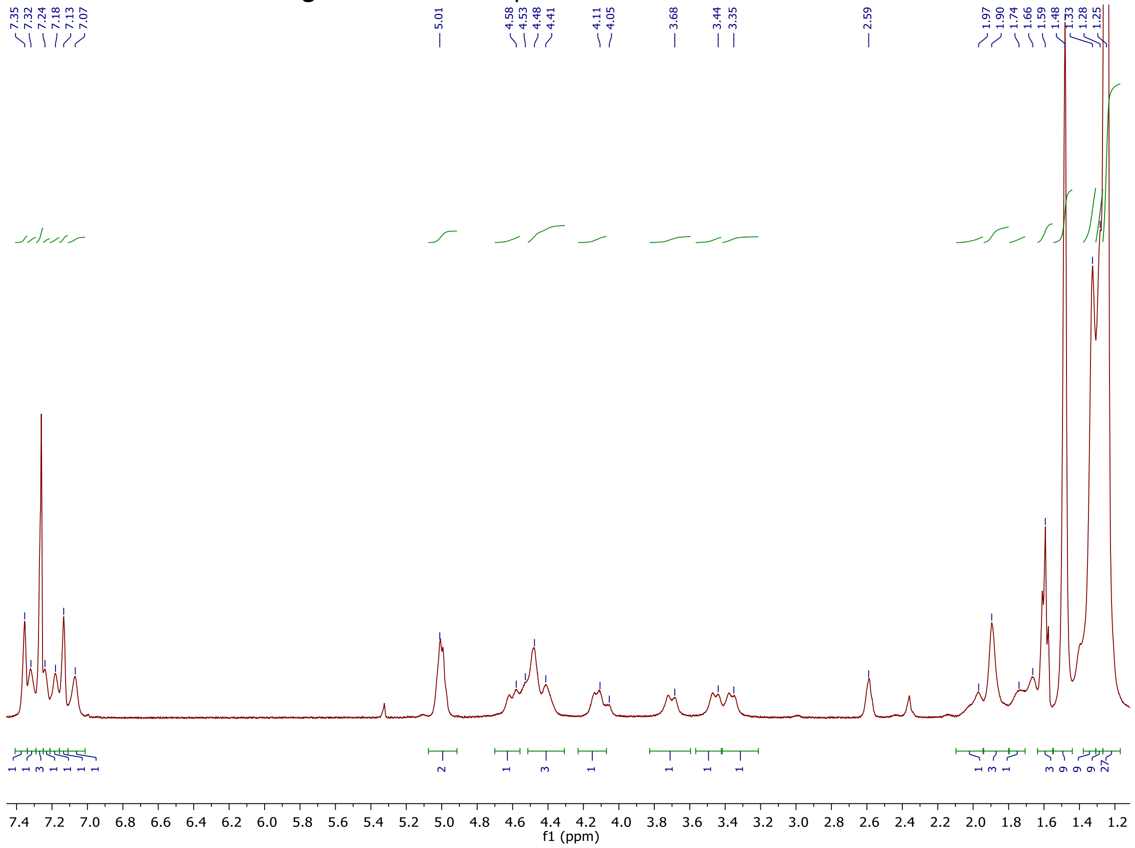

Figure S26. ^{1}H NMR spectrum of **5b** at 233 K in $CDCl_3$

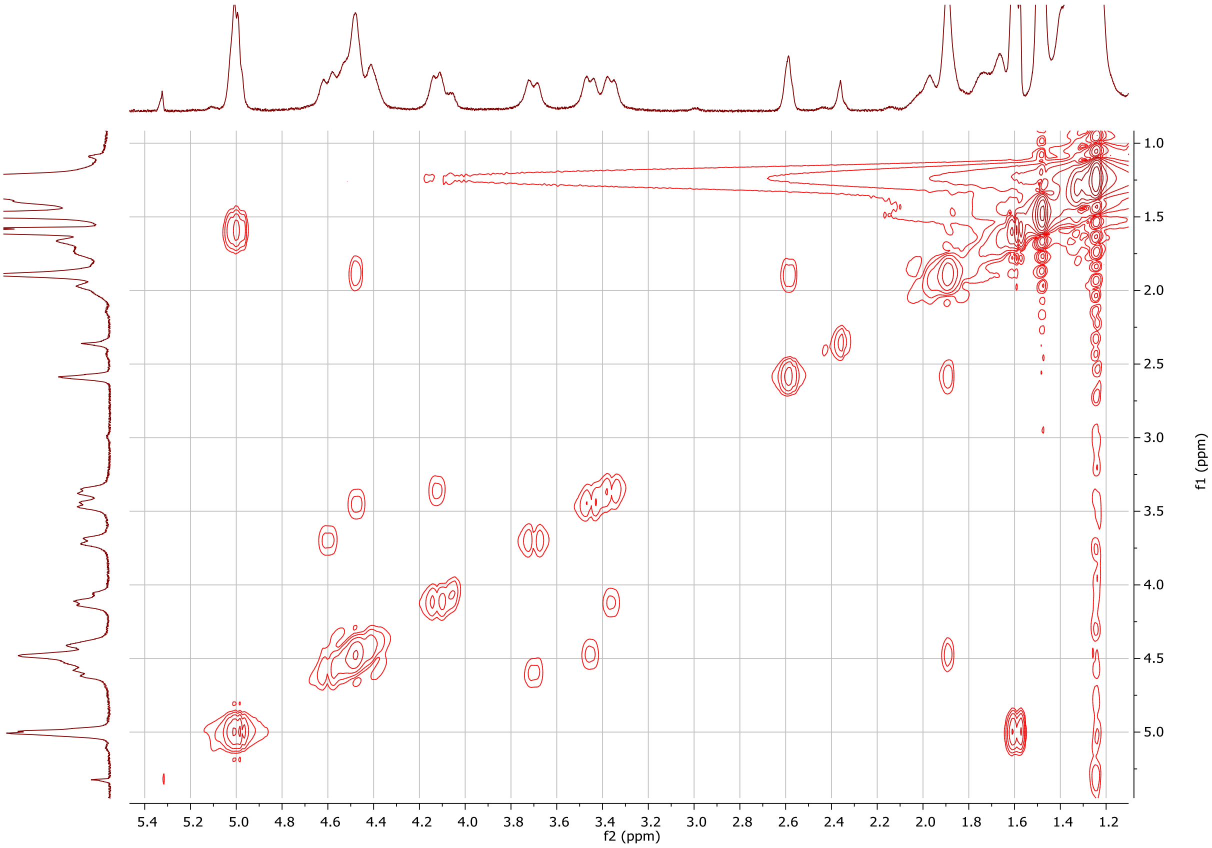

Figure S27. COSY NMR spectrum of **5b** at 233 K in $CDCl_3$

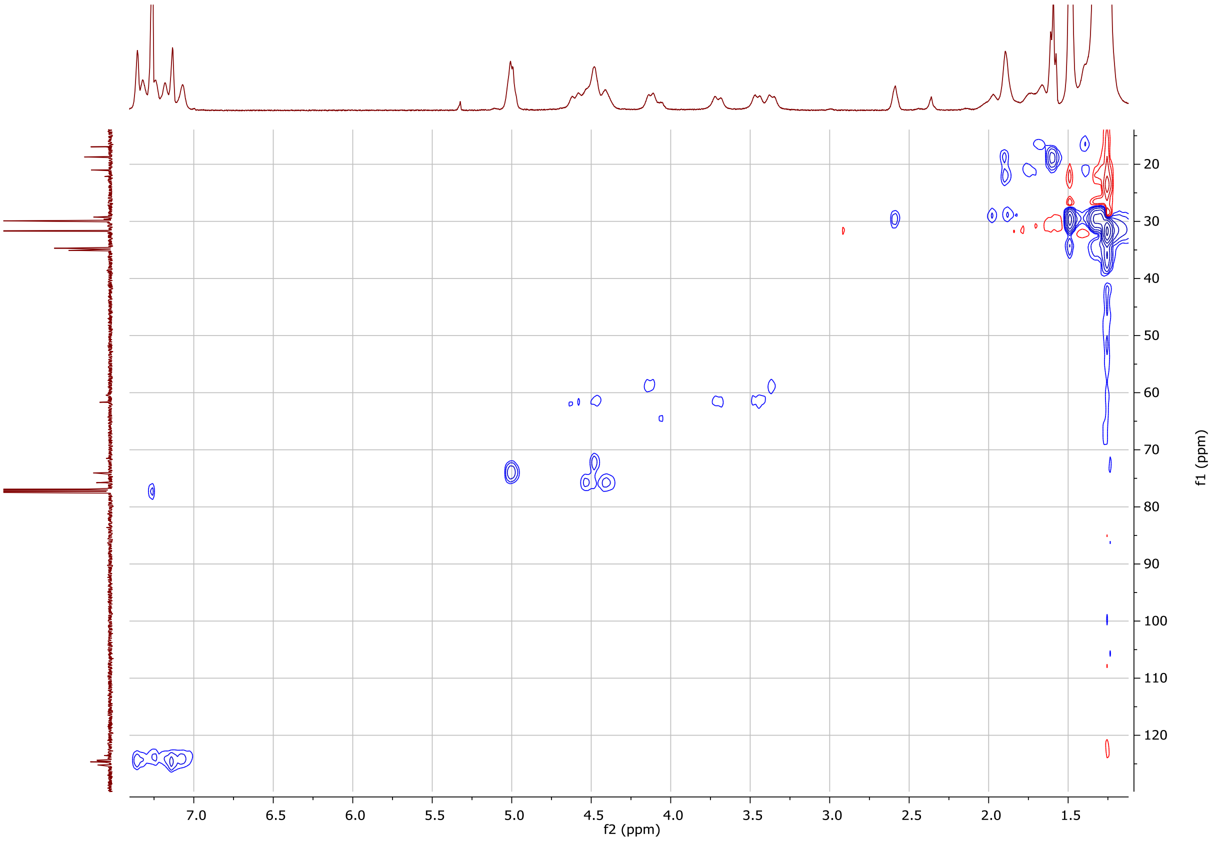

Figure S28. HSQC NMR spectrum of **5b** at 233 K in $CDCl_3$

Stoichiometric Experiments

Preparation of 5a from 3a by addition of δ-valerolactone

We have previously reported the formation of Nb ε-CL complex **4a** on addition of ε-CL to a suspension of **3a** in $CDCl_3$ at ambient temperature.[3] On addition to a suspension of **3a** in $CDCl_3$ at ambient temperature δ-VL exhibited analogous reactivity to ε-CL, yielding **5a**. Following addition of δ-VL ^1H NMR spectra were acquired at 298 K and 233 K, for comparison with ^1H NMR spectra of the pure complexes **3a** and **5a**, in $CDCl_3$ at each respective temperature. At 298 K, the well-resolved alkoxide OCH_2 signal of **3a** can be seen to shift from δ = 5.0 ppm to δ = 4.9 ppm on addition of δ-VL, characteristic of cleavage of the homobimetallic **3a** to yield **5a**.

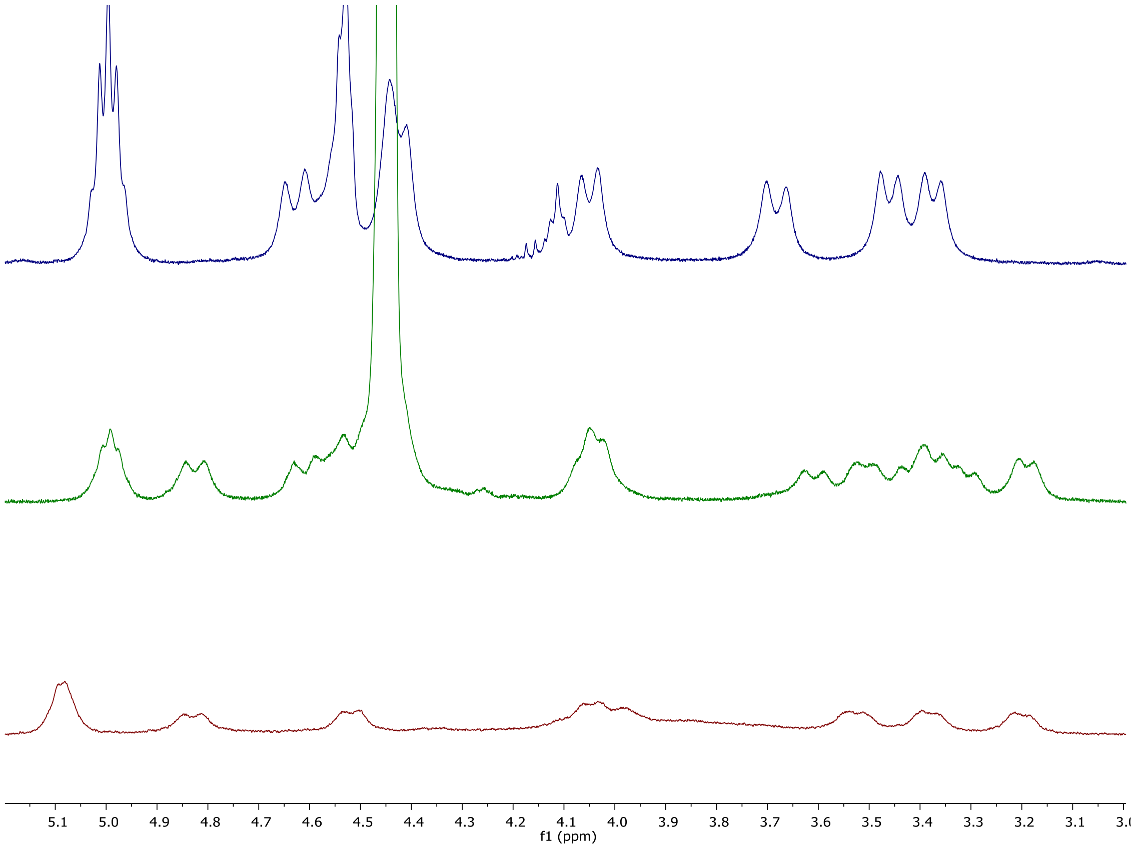

Figure S29. Stacked ^1H NMR spectra acquired at 233 K in $CDCl_3$, showing the methylene region of **3a** (red, bottom), **5a** (blue, top) and **3a** in the presence of excess δ-VL, showing formation of **5a** (green, middle). The intense signal at δ = 4.45 ppm corresponds to the OCH_2 protons of excess δ-VL.

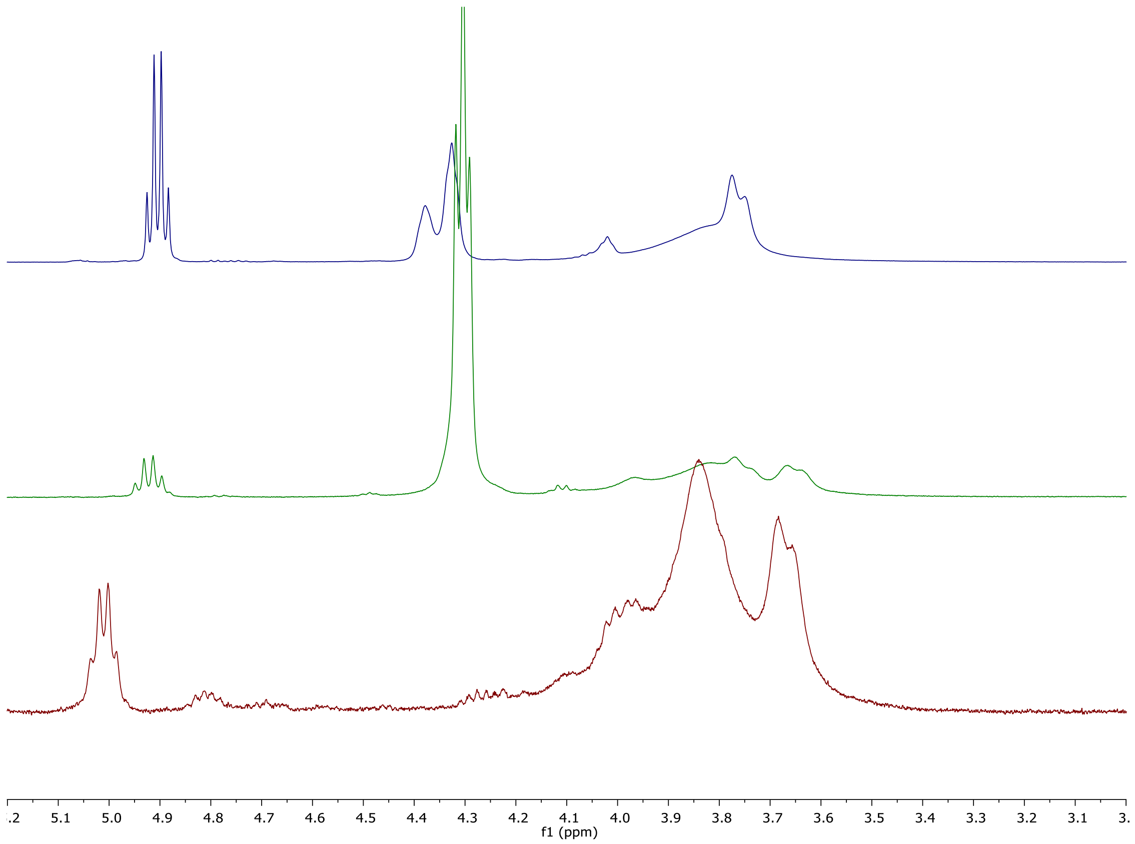

Figure S30. Stacked ^{1}H NMR spectra acquired at 298 K in $CDCl_3$, showing the methylene region of **3a** (red, bottom), **5a** (blue, top) and **3a** in the presence of excess δ-VL, showing formation of **5a** (green, middle). The intense signal at δ = 4.45 ppm corresponds to the OCH_2 protons of excess δ-VL.

Thermal stability of Nb complex 4a and Ta complex 4b

Two J Young's NMR tubes containing 1 ml of 0.017 mol dm^{-3} solutions of **4a** and **4b**, respectively, in $CDCl_3$ were heated simultaneously to 65 °C for 30 minutes, and then to 75 °C for a total of 165 minutes and 105 minutes, respectively, by submersion in a thermostat-controlled oil bath. The samples were periodically removed from the oil bath and analysed *via* ^{1}H NMR spectroscopy at ambient temperature. **4a** did not react at all when heated to 65 °C for 30 minutes, and complete consumption of that species was not observed until the sample had been heated to 75 °C for 165 minutes. By contrast, a small portion of the sample of **4b** was observed to have reacted at 65 °C, and complete conversion had occurred after 45 minutes heated to 75 °C. The ^{1}H NMR spectra acquired after this treatment showed that the methylene signals of the ethoxide moiety had migrated from δ = 4.96 ppm, and δ = 5.02 ppm, for **4a** and **4b** respectively, to δ = 4.85 ppm in both cases, corresponding to intramolecular nucleophilic attack yielding an ethyl ester of a single metal-coordinated, ring-opened ε-CL molecule. A signal also appeared in both cases at δ = 5.18 ppm, corresponding to the OCH_2 methylene protons of the metal-coordinated, ring-opened ε-CL moiety. Further signals were also present corresponding to the OCH_2 protons of oligo(ε-CL), originating from the ring-opening of residual (not metal-cordinated) ε-CL present in the samples of **4a** and **4b**. This was especially noticeable in the case of **4b** (see synthesis and characterisation of **4b**, above, and see main paper). On heating, both solutions of **4a** and **4b** gradually precipitated a white solid, attributed to decomposition of the complex formed following intramolecular attack, this presumably being kinetically unstable in the absence of further equivalents of ε-CL to occupy the resulting vacant coordination site.[3]

These observations confirm that initiation in the presence of either **4a** and **4b** proceeds by an identical route, and are entirely consistent with the proposed coordination-insertion mechanism. Furthermore,

whilst the more active initiator **4b** reacted fully during the heating time described, the ethoxide moiety of **4a** was only partially converted to the ethyl ester. This confirms that **4b** is more thermally unstable, and thus more active, than **4a**. The observed slow consumption of the ethoxide resonances of **4a** and **4b**, to yield the ethyl ester without formation of observable intermediate species, is consistent with the rate-determining step being the intramolecular nucleophilic attack, in agreement with previous computational studies concerning **4a**.[3] Finally, whilst the various Nb and Ta amine tris(phenolate) complexes are known to be sterically congested,[3] the difference in initiation rate for **4a** and **4b**, where in both cases ε-CL is already coordinated to the metal, rules out the coordination event as the origin of their difference in activity, although this is already known from our previous computational studies to be an exergonic event.[3]

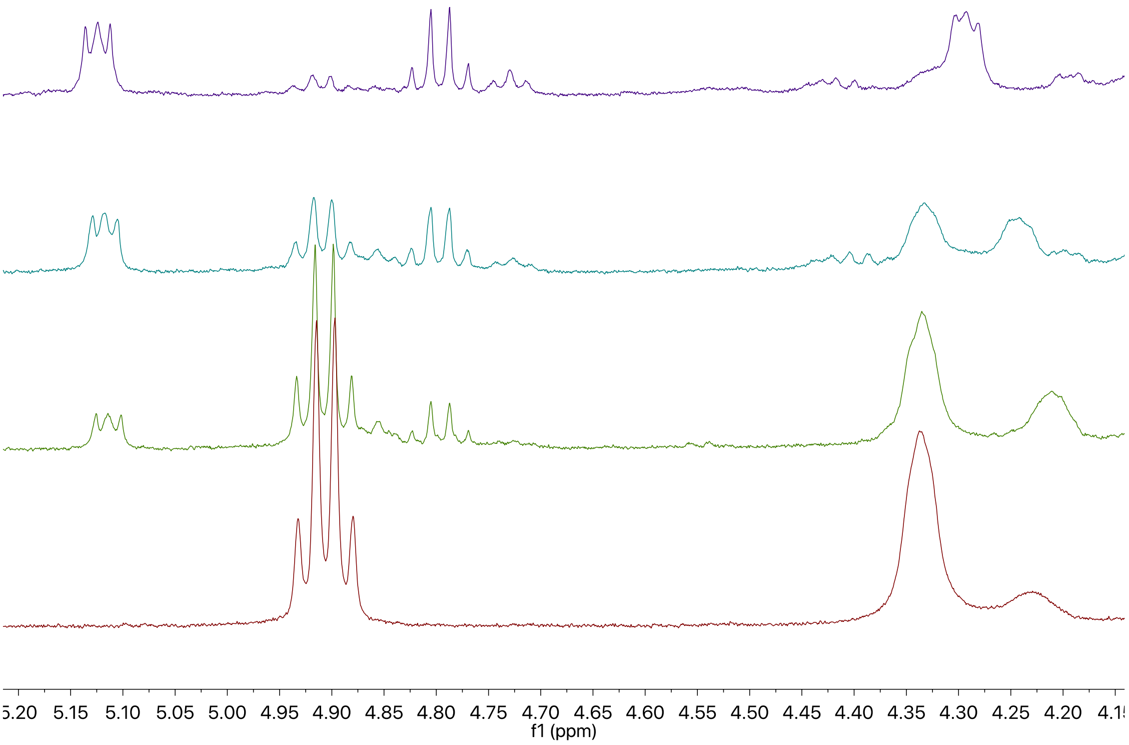

Figure S31. Stacked ^{1}H NMR spectra acquired at 298 K in $CDCl_3$, showing the methylene region of **4a** before heating (red, bottom) and after heating to 65 °C for 30 minutes and then to 75 °C for: 45 minutes (green, second from bottom), 105 minutes (blue, second from top) and 165 minutes (purple, top).

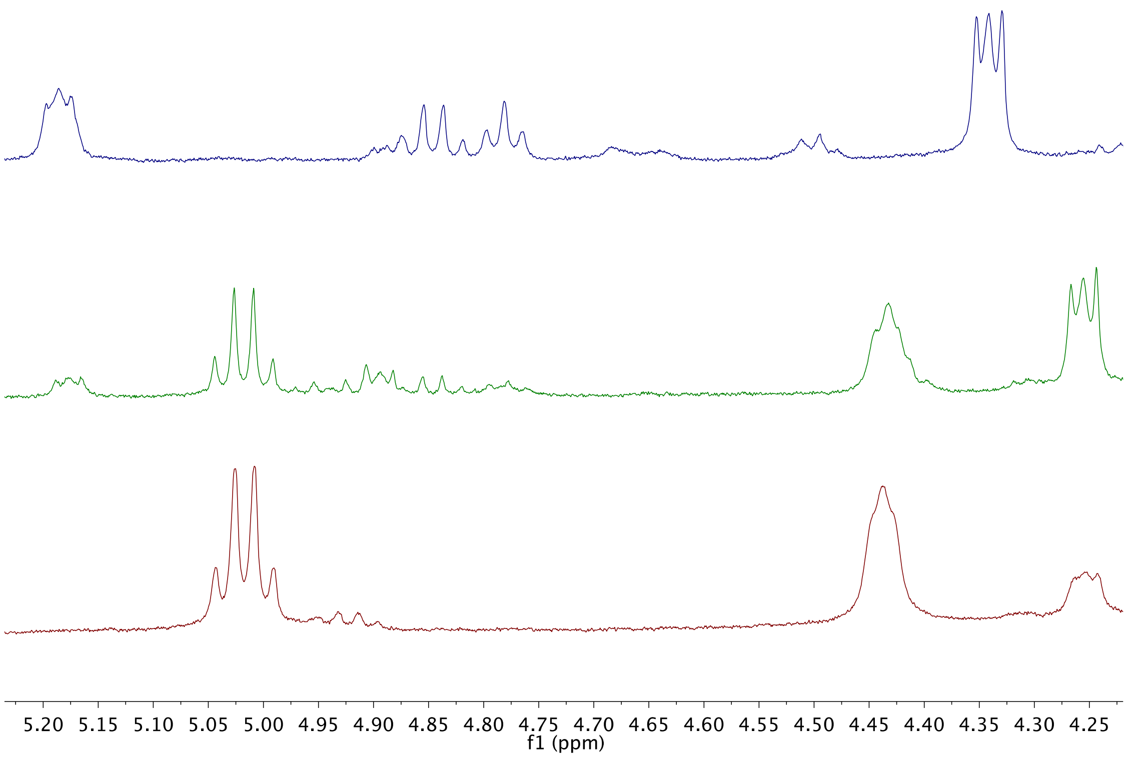

Figure S32. Stacked ^{1}H NMR spectra acquired at 298 K in $CDCl_3$, showing the methylene region of **4b** before heating (red, bottom), after heating to 65 °C for 30 minutes (green, middle) and after heating to 75 °C for a further 45 minutes (blue, top). Intramolecular nucleophilic attack to yield a metal-coordinated ethyl ester has proceeded to quantitative conversion.

Polymerisation studies

Example polymerisation procedure

In the glove box, a J Young's polymerisation tube (Schlenk flask) containing a magnetic stirring bead was charged with the solid catalyst. Then, 2.0 ml dry SPS toluene was added, followed by 200 mg ε-caprolactone (resulting in a monomer concentration of 0.80 mol dm^{-3}). The flask was sealed, removed from the glove box and placed into a silicone oil bath heated to 80 °C. After the desired reaction time had passed, the flask was cooled to 298 K, opened, and quenched by addition of 100 µL of a 0.75 mol dm^{-3} solution of benzoic acid in dichloromethane. 8 ml of dichloromethane was added to the reaction mixture, which was then transferred to a 25 ml round-bottomed flask.

Polymer analysis

For polymerizations of ε-caprolactone and δ-valerolactone, the solvent was removed with a rotary evaporator, and the remaining material retained for analysis. Conversion was determined by integration of the ^{1}H NMR methylene resonances corresponding to monomer and polymer species, at δ = 4.20 ppm and δ = 4.05 ppm (ε-CL), or δ = 4.34 ppm and δ = 4.07 ppm (δ-VL), respectively, in $CDCl_3$. Polymer molecular weights (g mol^{-1}) were determined *via* gel permeation chromatography (GPC), using a refractive index (RI) detector. Molecular weight values are reported after application of a conversion factor.[6,7] For polymerizations of β-butyrolactone, an aliquot of the reaction mixture was removed immediately after quenching with benzoic acid. This was added to a large excess of $CDCl_3$, and conversion determined by integration of ^{1}H NMR methine resonances corresponding to monomer and polymer species, at δ = 4.68 ppm and δ = 5.25 ppm, respectively. Where relevant, the ratio of ε-caprolactone units and ethoxy end groups in purified PCL samples was determined by integration of

OCH_2 methylene and methyl resonances corresponding to backbone and end group, respectively, in the ^{1}H NMR spectrum in benzene-d_6, $\left\{\left(M_{r,\varepsilon-CL} \times \frac{[H_{\varepsilon-CL}]}{[H_{OEt}]}\right) + M_r, EtOH\right\}$. Determination of P_r, the probability of racemic enchainment, for P3HB samples was *via* inverse gated (quantitative) ^{13}C NMR spectroscopy. The relative integrations of the methylene signals corresponding to *rr*, *rm*, *mr*, and *mm* triads were used, as described by Carpentier and co-workers.[8]

General procedure for kinetic studies

Kinetic experiments were carried out identically to the manner described above, except 100 mg of monomer was used, in 1.00 mol dm^{-3} solution in toluene-d_8 or chloroform-*d* in a J Young's NMR tube, without stirring. Immediately following addition of the monomer, the NMR tube was sealed, removed from the glove box, and cooled to 77 K for transport to the spectrometer. The probe was then heated to the desired temperature. The sample was warmed to room temperature, placed into the probe, and data acquisition begun immediately. Quenching and work-up procedures were identical to those described above.

Polymer analysis

Conversion for kinetic experiments was determined directly *via* integration of ^1H NMR methylene resonances in spectra acquired immediately prior to removal of the sample from the spectrometer. The signals corresponding to monomer and polymer species in toluene-d_8, respectively appeared at δ = 3.86 ppm and δ = 5.28 ppm for β-BL (methine), δ = 3.77 ppm and δ = 3.96 ppm for δ-VL (methylene), and δ = 3.65 ppm and δ = 3.98 ppm for ε-CL (methylene). In all cases, percentage conversion for kinetic plots of conversion *versus* time was calculated using Equation S1, where $A_{monomer}$ and $A_{polymer}$ are the integrated area of the monomer and polymer signals, respectively.

$$Conversion = 100 \times \left(\frac{A_{polymer}}{A_{polymer} + A_{monomer}}\right)$$

Equation S1.

Polymerization of δ-valerolactone

Poly(δ-valerolactone) produced in the presence of various loadings of both **4a** and **4b** exhibited a linear relationship between M_n^{Theo} and M_n^{GPC}, characteristic of a controlled living polymerization. Molecular weight control by addition of exogenous BnOH was also facile, confirming the immortal nature of the polymerisation kinetics.

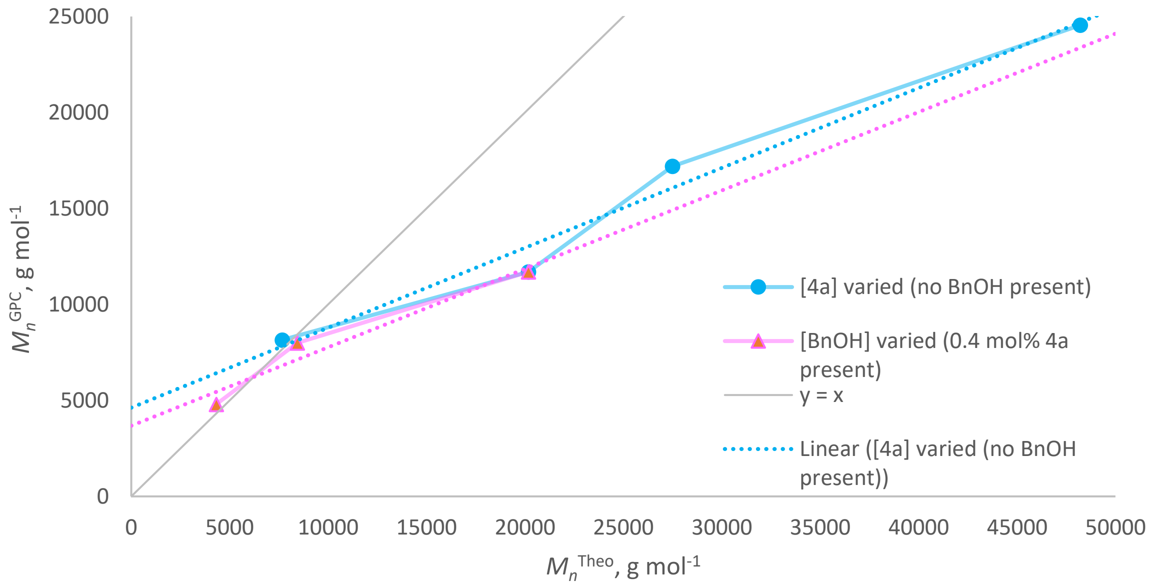

Figure S33. Plot of number average molecular weight (determined *via* GPC) against theoretical molecular weight for the ROP of δ-VL in the presence of Nb complex **4a** and in the presence of BnOH where relevant. Although adherence to theoretical values is not good, the linear relationship between theoretical and experimental values allows predictable control of molecular weight.

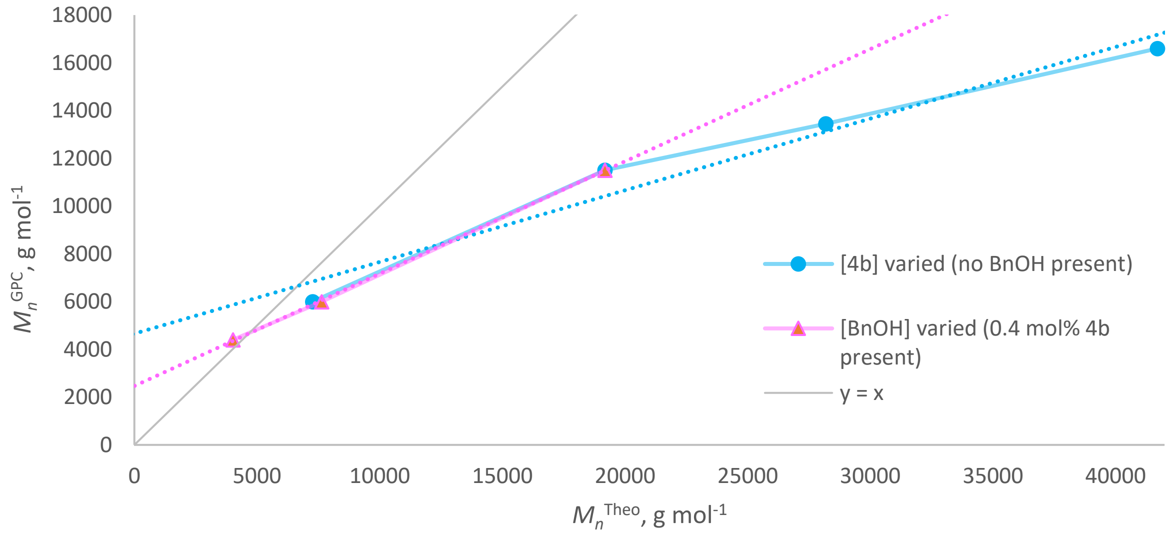

Figure S34. Plot of number average molecular weight (determined *via* GPC) against theoretical molecular weight for the ROP of δ-VL in the presence of Ta complex **4b** and in the presence of BnOH where relevant. Although adherence to theoretical values is not good, the linear relationship between theoretical and experimental values allows predictable control of molecular weight.

Polymerization of *rac*-β-butyrolactone

Data corresponding to the ROP of *rac*-β-butyrolactone is presented in Table S2. Kinetic studies were carried out in the presence of **4a** and **4b** at 80 °C, 60 °C, and 25 °C. At 80 °C and 60 °C molecular weight control was extremely poor, but **4b** was much more active than **4a** (see main paper). At 25 °C, both **4a** and **4b** were inactive. Kinetic studies of the ROP of β-BL in the presence of **3a** were also undertaken, at 60 °C and 25 °C, exhibiting activity without an induction period in both cases (see main paper and relevant sections, below). Molecular weight control studies were carried out in the presence of **4a** and **4b** at 65 °C, in the presence of **3a** at 65 °C and 25 °C, and in the presence of **5a** and **5b** at 65 °C. Molecular weight control was extremely poor in all cases, with extremely low M_n^{GPC} values attributed to cyclisation of the P3HB product, although $Đ_M$ remained consistently low.

Table S1. Polymerization data for the ROP of β-BL in the presence of **4a**, **4b**, **3a**, **5a** and **5b**

Entry	Catalyst	[Monomer]/[Cat.]	Duration, min	Temp, °C	[h]Conversion, %	[i,j]M_n^{Theo}, g mol^{-1}	[k]M_n^{GPC}, g mol^{-1}	[k]$Đ_M$	[l]P_r	[m]k_{obs}, min^{-1}
1[a]	**4a**	100	100	65	100	8769[i]	1550	1.27	0.44	N/A
2[b]	**4a**	400	120	65	100	34596[i]	1850	1.30	0.50	N/A
3[b]	**4a**	1000	240	65	100	86250i	2050	1.33	0.47	N/A
4[a]	**4b**	100	60	65	100	8769i	1550	1.39	0.48	N/A
5[b]	**4b**	400	80	65	100	34596i	1900	1.33	0.46	N/A
6[b]	**4b**	1000	140	65	100	86250[i]	2100	1.35	0.42	N/A
7[b]	**4b**	200	15	65	65	11352[i]	1350	1.25	N/A	N/A
8[a]	**3a**	100	90	65	100	8655[j]	1800	1.39	N/A	N/A
9[b]	**3a**	400	150	65	100	34482[j]	1900	1.34	N/A	N/A
10[b]	**3a**	500	120	65	100	43091[j]	1850	1.39	N/A	N/A
11[b]	**3a**	1000	150	65	100	86136[j]	2000	1.36	N/A	N/A
12[b]	**3a**	1000	30	65	55	47396[j]	1700	1.36	N/A	N/A
13[c]	**3a**	100	150	25	89	7708[j]	2200	1.25	N/A	N/A
14[c]	**3a**	200	300	25	60	10377[j]	2600	1.37	N/A	N/A
15[c]	**3a**	300	450	25	42	10893[j]	3000	1.17	N/A	N/A
16[d]	**3a**	400	300	25	49	16920[j]	1350	1.47	N/A	N/A
17[d]	**3a**	1000	720	25	27	23290[j]	2100	1.52	N/A	N/A
18[e]	**4a**	200	21	80	96	16689[i]	1500	1.30	N/A	0.36
19[e]	**4b**	200	9	80	95	16517[i]	1650	1.34	N/A	0.75
20[e]	**4a**	200	100	60	99	17206[i]	1700	1.30	0.37	0.065
21[e]	**4a**	400	125	60	99	34252[i]	1750	1.30	0.49	0.030
22[e]	**4b**	200	54	60	99	17206[i]	1800	1.31	0.42	0.098
23[e]	**4b**	400	70	60	99	34252[i]	1850	1.32	0.48	0.049
24[e]	**3a**	100	69	60	97	8397[j]	N/A	N/A	N/A	0.032[n]
25[e]	**3a**	400	204	60	98	33793[j]	2250	1.38	N/A	0.013[n]
26[f]	**4a**	100	2956	25	16	1538[i]	N/A	N/A	N/A	0.0003
27[f]	**4b**	100	680	25	22	2054[i]	N/A	N/A	N/A	0.0005
28[f]	**3a**	100	1110	25	100	8655[j]	2750	1.47	N/A	0.0095
29[g]	**5a**	100	90	60	100	8755[i]	1600	1.30	N/A	0.1491
30[g]	**5a**	200	100	60	100	17364[i]	1700	1.26	N/A	0.0666
31[g]	**5b**	100	50	60	100	8755[i]	1300	1.30	N/A	N/A[o]
32[g]	**5b**	200	45	60	100	17364[i]	1450	1.28	N/A	0.2066

Conditions: [a] 200 mg β-BL; 0.80 mol dm^{-3} in toluene. [b] 400 mg β-BL; 0.80 mol dm^{-3} in toluene. [c] 200 mg β-BL; 0.80 mol dm^{-3} in dichloromethane. [d] 400 mg β-BL; 0.80 mol dm^{-3} in dichloromethane. [e] 100 mg β-BL; 1.00 mol dm^{-3} in toluene-d_8, in a J Young's NMR tube, unstirred (kinetic studies). [f] 100 mg β-BL; 1.00 mol dm^{-3} in chloroform-*d*, in a J Young's NMR tube, unstirred (kinetic studies). [g] 100 mg β-BL; 1.00 mol dm^{-3} in toluene-d_8 in a J Young's NMR tube, unstirred (kinetic studies). [h] Conversion determined *via* ^{1}H NMR spectroscopy, by integration of the monomer and polymer OC*H* methine resonances. [i] M_n^{Theo} for polymerizations in the presence of **4a**, **4b**, **5a** or **5b** calculated from conversion and catalyst concentration, $\left\{\left(M_{r,monomer} \times \frac{\%_{conv}}{100} \times \frac{[monomer]}{[Cat.]}\right) + M_{r,EtOH} + M_{r,coordinated\ lactone}\right\}$, where the coordinated lactone is either ε-CL (**4a**, **4b**) or δ-VL (**5a**, **5b**). [j] M_n^{Theo} for polymerizations in the presence of **3a** calculated from conversion and catalyst concentration, $\left\{\left(M_{r,monomer} \times \frac{\%_{conv}}{100} \times \frac{[monomer]}{[Cat.]}\right) + M_{r,EtOH}\right\}$. [k] Determined *via* GPC analysis in THF using a refractive index detector and with application of a conversion factor of 0.54.[7] [l] Determined *via* inverse-gated (quantitative) ^{13}C NMR spectroscopy. [m] Determined *via* ^{1}H NMR spectroscopic reaction monitoring. [n] significant deviation from pseudo-first order kinetics observed, due to slow catalyst solubilization. [o] Technical difficulties encountered during acquisition of kinetic data.

Kinetics of *rac*-β-butyrolactone ROP at 80 °C in toluene-d_8

Although both **4a** and **4b** were highly active at 80 °C, the reaction proceeded too quickly for accurate kinetic study using the techniques described herein. It was also considered that, unstirred in a J Young's NMR tube, the period required for the sample to reach the required temperature, and for the poorly-soluble catalyst to enter a homogeneous solution would be significant with respect to the timescale of the reaction at 80 °C. As such, all further studies were carried out at lower temperatures. Nonetheless, a considerable catalyst induction period is apparent in the cases of both initiators, and 4b exhibited much higher activity than **4a**; observations that were confirmed and explored further *via* kinetic studies at 60 °C.

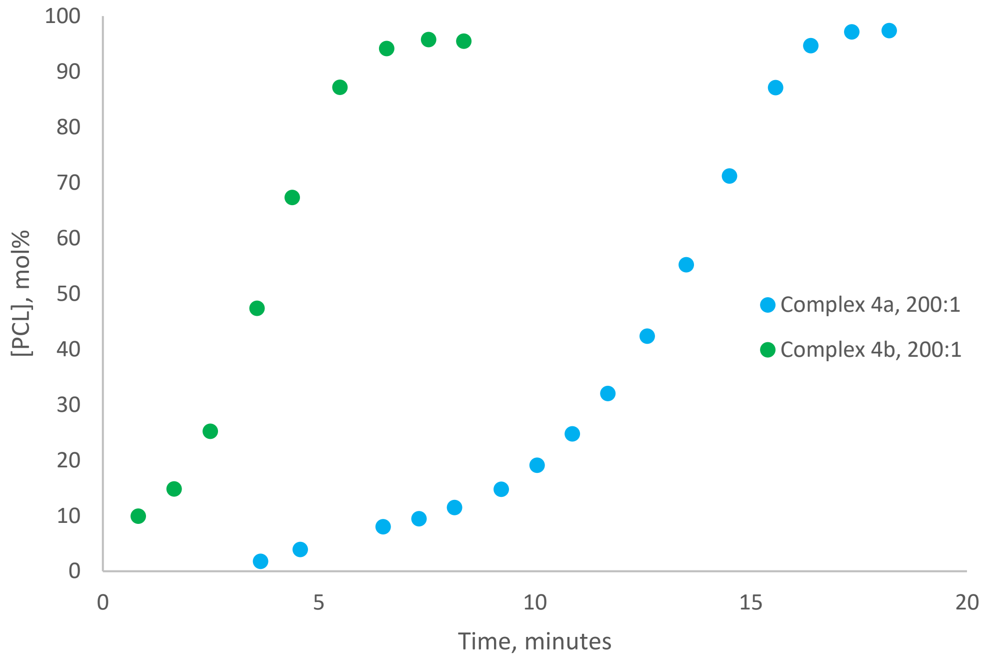

Figure S35. Plots of percentage conversion *versus* time for the ROP of β-BL in toluene-d_8 at 80 °C in the presence of 0.5 mol% of initiators **4a** and **4b**, respectively

Comparison of *rac*-β-butyrolactone ROP kinetics in the presence of 3a and 4a, at 60 °C in toluene-d_8

The ROP of β-BL in the presence of initiators **3a** and **4a** at 60 °C in toluene produced distinct reaction profiles. Whereas **4a** exhibited an induction period, attributed to slow ring-opening of metal-coordinated ε-CL, **3a** did not exhibit such an induction period. This is due to the facile elimination of [$L^{tBu}NbF_2$] on addition of β-BL, to form the active species, without requiring the prior intramolecular nucleophilic attack, and ring-opening of a less reactive, coordinated, monomer. Although use of **3a** eliminated the induction period, the time required to achieve full conversion under the conditions used for kinetic experiments, was similar, and the maximum rate was lower, than for **4a**. This is due to the extremely poor solubility of **3a** in toluene-d_8, meaning the effective catalyst concentration in solution remained very low throughout the reaction. It is feasible that in a more polar solvent, and with more vigorous stirring, **3a** may offer a significant reduction in reaction time, *versus* **4a**. The catalyst loading was reduced to 0.25 mol% for this study in an effort to minimise the difference in the concentration of dissolved Nb species between those polymerisations in which **3a** and **4a** were used, arising from the low solubility of **3a**. However, this effect could not be completely eliminated, because for experiments on the NMR-scale, masses of initiator corresponding to loadings below 0.25 mol% could not be accurately weighed out, and the extremely poor solubility of **3a** in $CDCl_3$ in the absence of the monomer precluded the possibility of preparation of a stock solution. The observed absence of an induction period for **3a**, however, is only more significant given the poor solubility of that species.

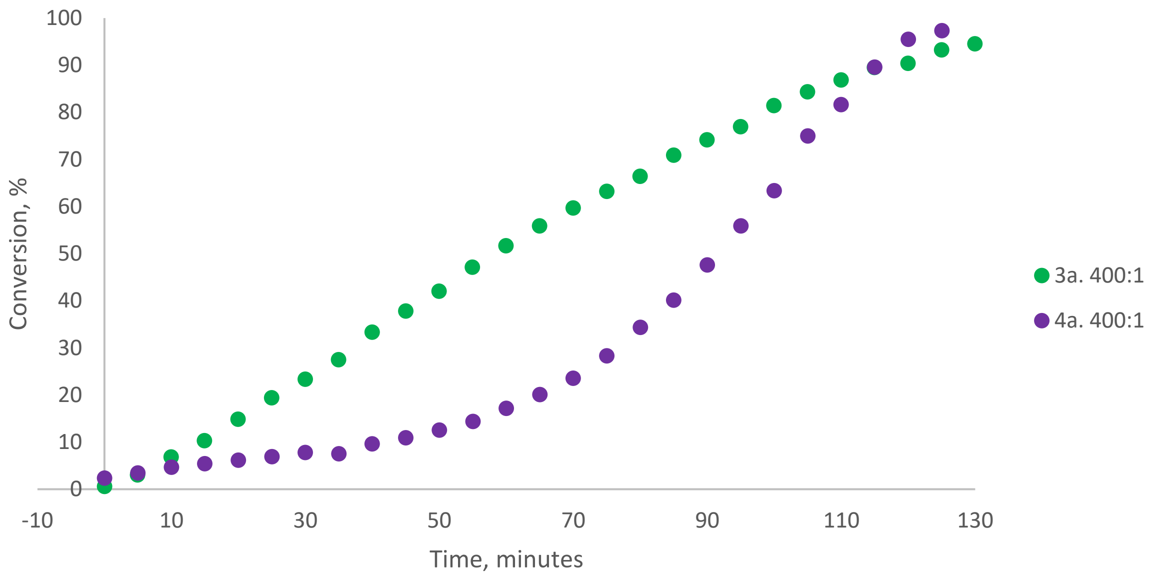

Figure S36. Plots of percentage conversion *versus* time for the ROP of β-BL in toluene-d_8 at 60 °C in the presence of 0.25 mol% of initiators **3a** and **4a**, respectively

Comparison of of *rac*-β-butyrolactone ROP kinetics in the presence of 3a, 4a and 4b, at 25 °C in $CDCl_3$

Both **4a** and **4b** exhibited negligible activity for the ROP of β-BL at 25 °C, whereas pre-catalyst **3a** readily polymerised β-BL under the same conditions. This is consistent with the ROP of β-BL in the presence of **4a** and **4b** (and presumably **5a** and **5b**) at higher temperatures proceeding first *via* intramolecular nucleophilic attack and ring-opening of the metal-coordinated lactone moiety of the pre-catalyst. **3a** does not bear a coordinated lactone molecule, and is known to readily undergo cleavage to yield **4a** or **5a** on addition of ε-CL or δ-VL, respectively. It is therefore expected that displacement of the neutral fragment [$L^{tBu}NbF_2$] would readily occur on addition of β-BL to **3a**, yielding an active cationic species. The facile nature of the subsequent polymerisation of β-BL precludes isolation of such a complex. The ring-opening of ε-CL or δ-VL is not favourable at 25 °C, and thus those systems for which initiation proceeds first *via* such an event, are inactive at that temperature.

The failure of β-BL to displace coordinated ε-CL or δ-VL molecules is presumably kinetic in origin, as otherwise even a more weakly-coordinating monomer may be anticipated to yield some activity when added to the ε-CL- or δ-VL-bearing initiator in 100-fold excess.

For this study, the catalyst loading was increased to 1 mol%, due to the expected slow reaction kinetics at 25 °C. As anticipated, much of the initiator **3a** remained undissolved under those conditions (although **3a** appears much more soluble in $CDCl_3$ than in toluene-d_8). However, the resulting reduction in the concentration of **3a** relative to **4a** and **4b** in their respective solutions only increases the significance of the observed dramatic difference in activity between the bimetallic (**3a**) and monometallic (**4a**, **4b**) systems.

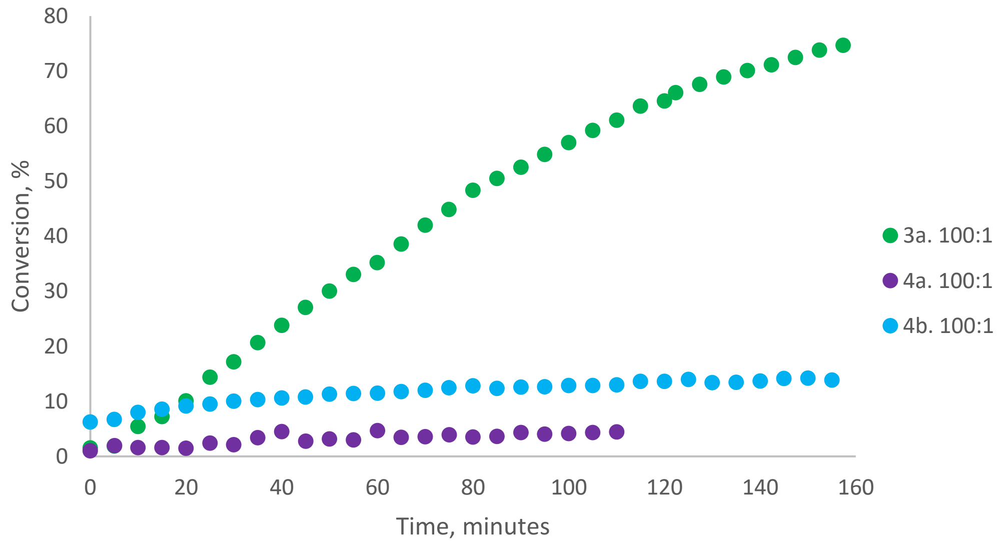

Figure S37. Plots of percentage conversion *versus* time for the ROP of β-BL in $CDCl_3$ at 25 °C in the presence of 1 mol% of initiators **3a**, **4a** and **4b** respectively.

Additional data for δ-valerolactone ROP at 60 °C in toluene-d_8

The ROP of δ-VL in the presence of each of **4a** and **4b** was carried out at two catalyst loadings, exhibiting an apparent first order rate dependency with respect to the catalyst concentration. Moreover this confirms the observed inconsistency in the rate difference observed between δ-VL and ε-CL polymerisations with Nb and Ta systems (Significant difference observed for Nb but not for Ta; see main paper).

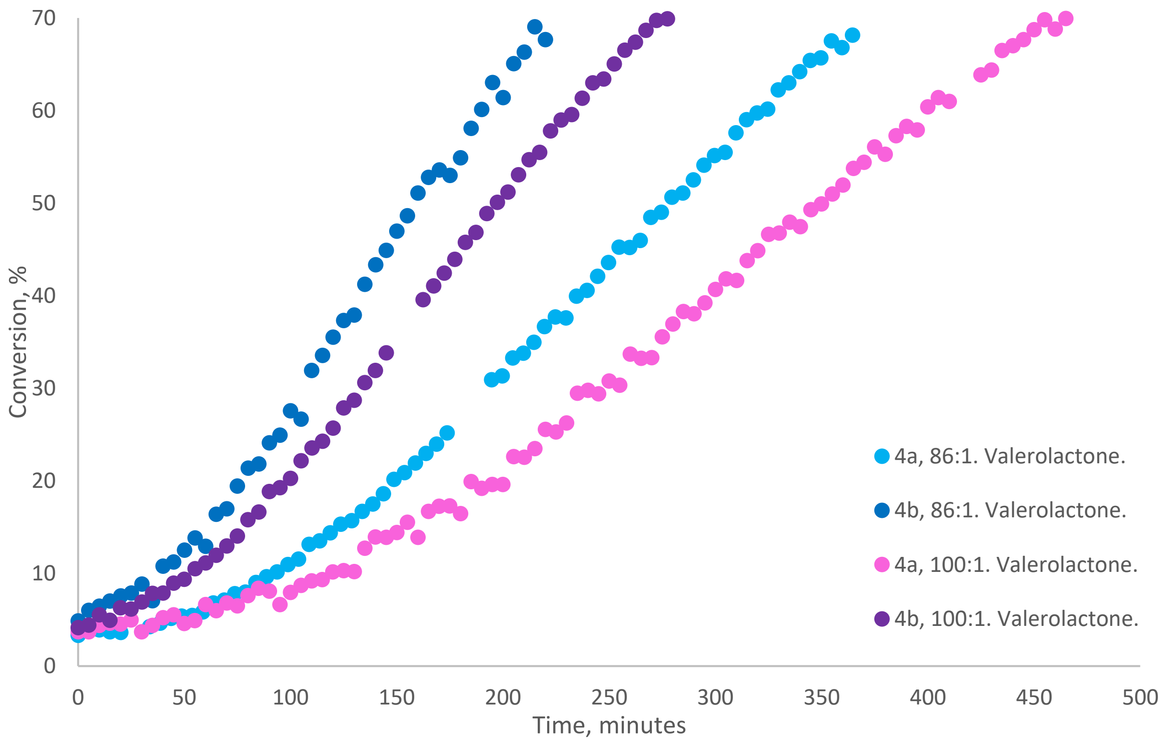

Figure S38. Plots of percentage conversion *versus* time for the ROP of δ-VL in toluene-d_8 at 60 °C in the presence of 1 mol% and 1.16 mol% of initiators **4a** and **4b**, respectively.

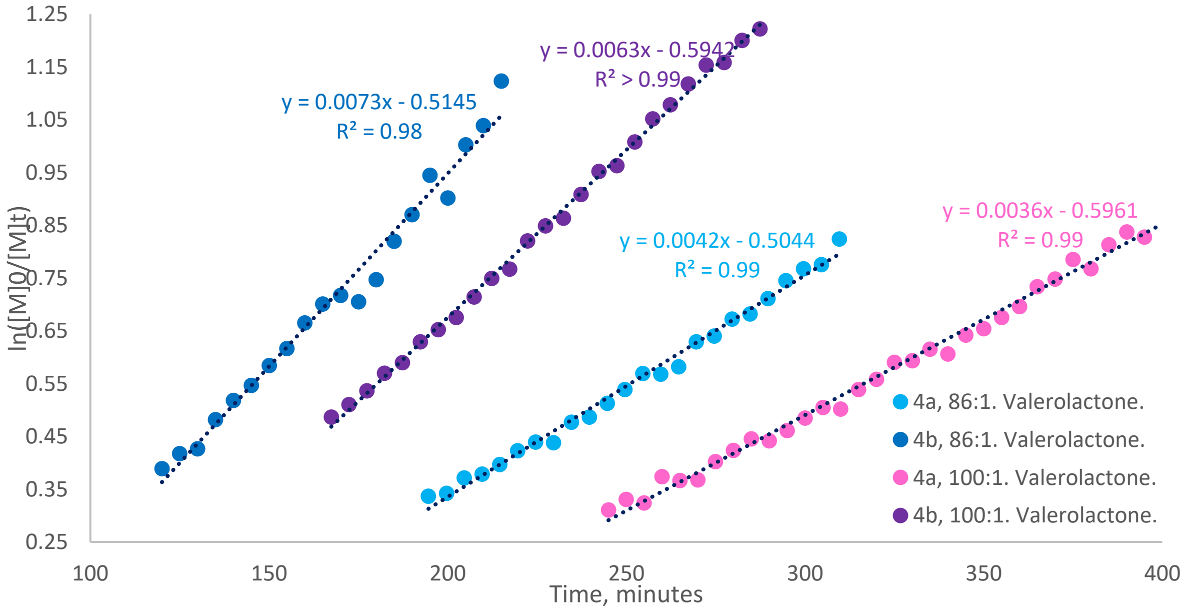

Figure S39. Semi-logarithic initial rate plots for the ROP of δ-VL in toluene-d_8 at 60 °C in the presence of 1 mol% and 1.16 mol% of initiators **4a** and **4b**, respectively.

Control experiments

Attempted ROP of *L*-lactide and *rac*-lactide

The polymerization of lactides was attempted in the presence of Ta complex **4b** at 80 °C in *protio*-toluene. In all cases, no activity was observed. This is attributed to steric congestion arising from the methyl groups of the lactide monomer α to the carbonyl and the constrained nature of the active site.[3]

Table S2. Data for the attempted ROP of lactides in the presence of **4a** and **4b**

Entry	*Monomer*	*Catalyst*	*[Monomer]/[Cat.]*	*[Cat.], mol%*	*[a] Conversion, %*
1	*L*-LA	**4b**	100	1	0
2	*rac*-LA	**4b**	100	1	0
3	*L*-LA	**4b**	220	0.45	0
4	*rac*-LA	**4b**	220	0.45	0

Conditions: 250 mg lactide; 0.80 mol dm^{-3} in toluene; 360 minutes at 80 °C. [a] Determined *via* ^{1}H NMR spectroscopy.

Attempted catalytic use of neutral Nb and Ta complexes for ROP of lactones

Neutral group 5 metal alkoxide complexes **1b** and **2b**, synthetic precursors to cationic species **4b** and **5b**, were screened as initiators for the ROP of ε-CL at 80 °C in *protio*-toluene, exhibiting no activity (Nb complexes **1a** and **2a** have been shown previously to be inactive for the ROP of ε-CL under the same conditions). Similarly, complexes **1a**, **1b**, **2a** and **2b** were screened for activity in the ROP of β-BL, which in the current work has been found to be much more reactive in the presence of cationic Nb and Ta species than ε-CL or δ-VL. All of the neutral complexes were found to be entirely inactive for the ROP of β-BL.

Table S3. Data for the attempted ROP of ε-CL and β-BL in the presence of neutral complexes **1a, 1b, 2a** and **2b**

Entry	*Monomer*	*Catalyst*	*[Monomer], mol dm3*	*[a] Conversion, %*
1	ε-CL	**1b**	0.8	0
2	ε-CL	**2b**	0.8	0
3	β-BL	**1a**	1	0
4	β-BL	**2a**	1	0
5	β-BL	**1b**	1	0
6	β-BL	**2b**	1	0

Conditions: 200 mg monomer; 360 minutes in toluene at 80 °C; 1 mol% catalyst. [a] Determined *via* ^{1}H NMR spectroscopy.

End-group analysis

Quantification of ethoxy end groups

Where ^{1}H NMR spectroscopy was required to quantify ethoxy end groups in poly(ε-caprolactone), the polymer was purified by dissolving in dichloromethane and precipitating from methanol. The solid residue was then washed copiously with methanol over a glass frit, and dried overnight under dynamic vacuum. ^{1}H NMR spectra were then acquired in C_6D_6 at 298 K. Signals corresponding to the methylene signals of the polymer backbone, and the methyl signals of the ethoxy end group appeared at δ = 3.99 ppm and δ = 0.99 ppm, respectively.

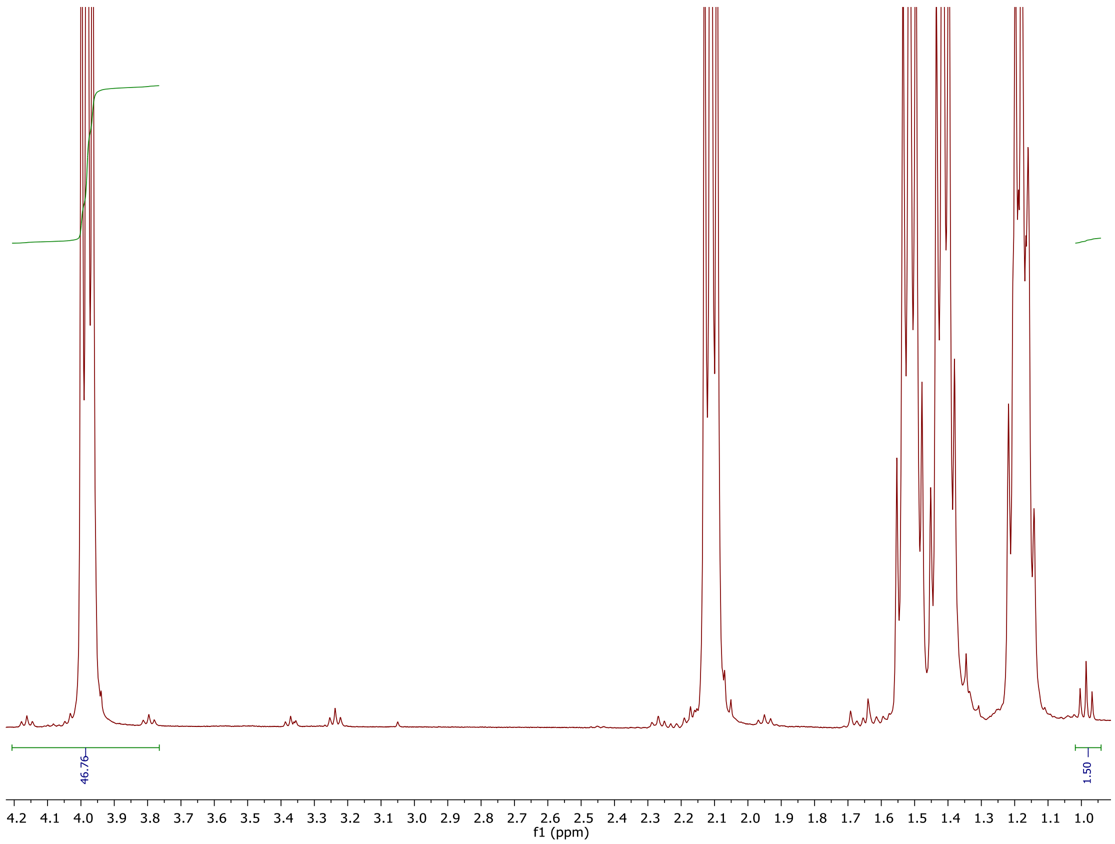

Figure S40. ^{1}H NMR spectrum in C_6D_6 at 298 K of the purified PCL product from the ROP of ε-CL at 80 °C in *protio*-toluene in the presence of 2.22 mol% of initiator **4b** ([ε-CL]:[**4b**] = 45:1). Signals at δ = 4.0 ppm and δ = 1.0 ppm correspond to PCL backbone OCH_2 and ethoxy end-group CH_3 protons, respectively.

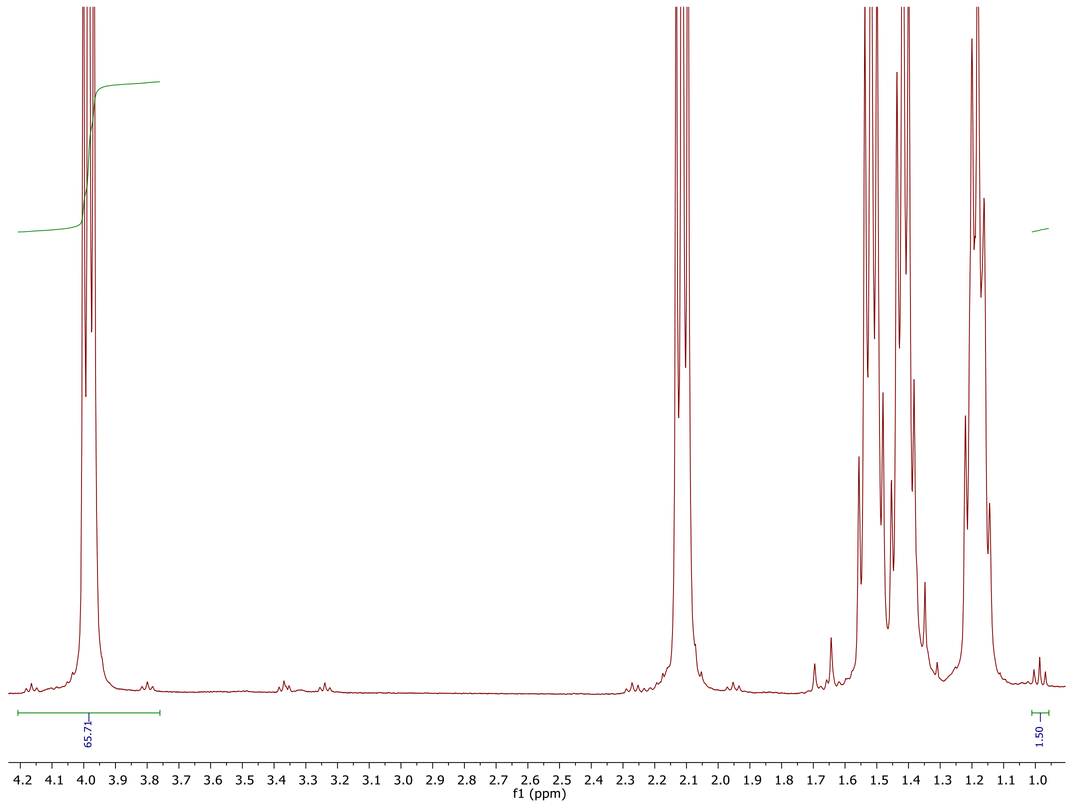

Figure S41. ^{1}H NMR spectrum in C_6D_6 at 298 K of the purified PCL product from the ROP of ε-CL at 80 °C in *protio*-toluene in the presence of 1.48 mol% of initiator **4b** ([ε-CL]:[**4b**] = 67.5:1). Signals at δ = 4.0 ppm and δ = 1.0 ppm correspond to PCL backbone OCH_2 and ethoxy end-group CH_3 protons, respectively.

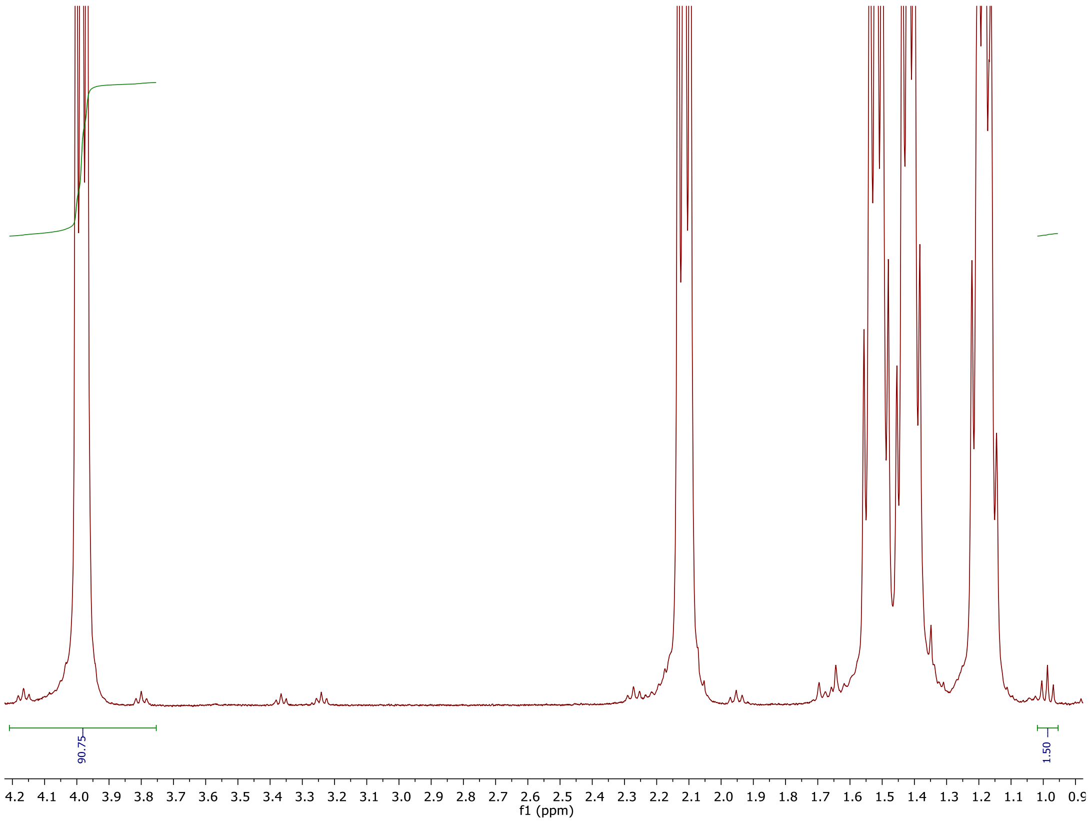

Figure S42. ^{1}H NMR spectrum in C_6D_6 at 298 K of the purified PCL product from the ROP of ε-CL at 80 °C in *protio*-toluene in the presence of 1.11 mol% of initiator **4b** ([ε-CL]:[**4b**] = 90:1). Signals at δ = 4.0 ppm and δ = 1.0 ppm correspond to PCL backbone OCH_2 and ethoxy end-group CH_3 protons, respectively.

Quantification of ε-CL unit in P3HB

Where ^{1}H NMR spectroscopy was required to quantify ε-caprolactyl residues in poly-3-hydroxybutyrate, the reaction was quenched by addition of benzoic acid at 65% conversion, and the polymer purified by the following procedure: The polymer was dissolved in cold methanol, which was then cooled on dry ice to precipitate P3HB. The methanol was then decanted, and the residue washed with further cold methanol. After drying overnight under dynamic vacuum, the solid material was dissolved in $CDCl_3$ and a ^{1}H NMR spectrum acquired at 298 K. Signals corresponding to ε-caprolactyl methylene protons appear at δ = 4.05 ppm (1 methylene group) and δ = 1.62 ppm (2 methylene groups), and β-butyrolactyl methylene and methine proton signals appear at δ = 5.24 ppm and δ = 2.35-2.80 ppm, respectively. Those signals were used to quantify the ε-caprolactyl residue in the P3HB chain. The quantity of ε-CL incorporated into the P3HB product is entirely consistent with initiation of β=BL ROP in the presence of **4b** proceeding *via* slow ring-opening of the coordinated ε-CL molecule of the initiator, as indicated by our kinetic studies (see main paper).

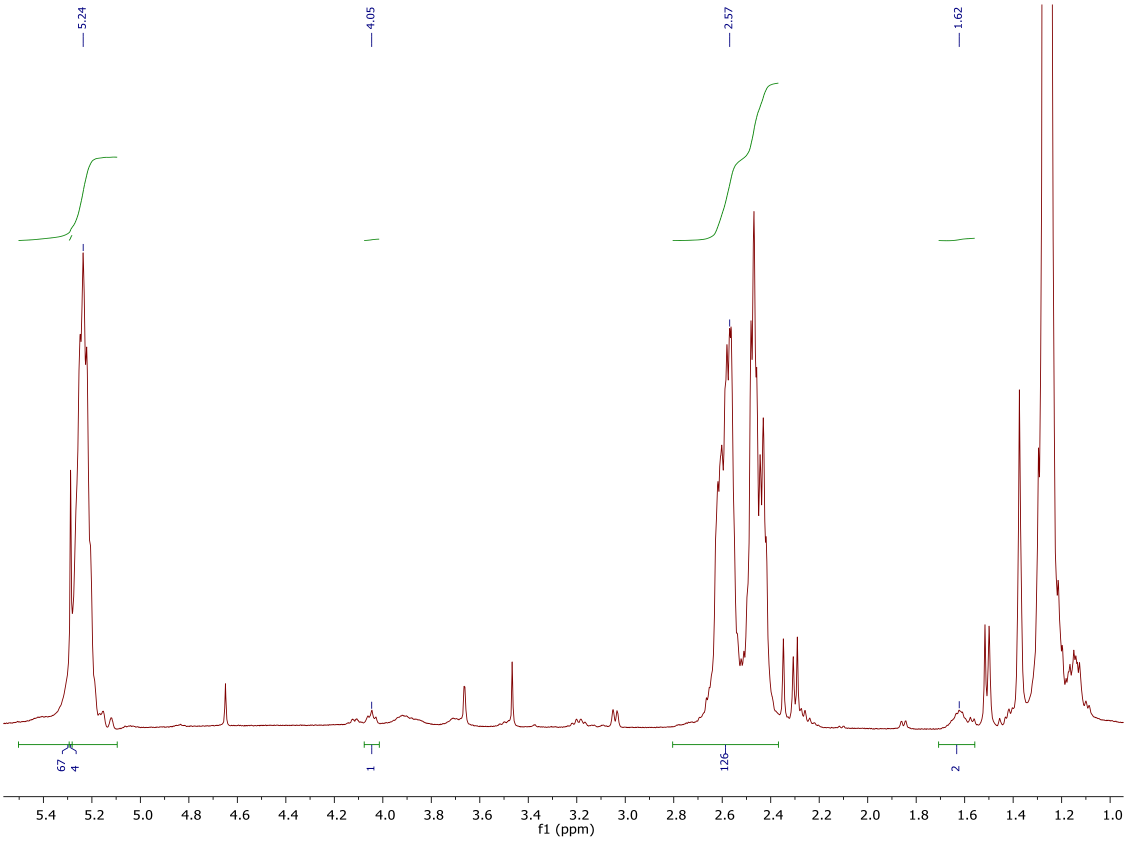

Figure S43. ^{1}H NMR spectrum in $CDCl_3$ at 293 K of the purified P3HB product from the ROP of β-BL at 65 °C in *protio*-toluene in the presence of 0.5 mol% of initiator **4b** ([β-BL]:[**4b**] = 200:1), quenched after reaching 65% conversion. Signals at δ = 5.24 ppm and δ = 2.35-2.80 ppm correspond to the $OCHCH_3$ and $O{=}CCH_2$ protons of the P3HB backbone, respectively, and signals at δ = 4.05 ppm and δ = 1.62 ppm correspond to the OCH_2 and $(CH_2)_2$ protons of a ε-caprolactone unit, respectively.

MALDI-TOF-MS spectra

MALDI-TOF-MS analysis of P3HB samples produced in the presence of **3a**, **4a** and **4b** showed that formation of cyclic polymer species was favoured, although other species were also present. All series detected had a repeat unit corresponding to that of P3HB. Polymers containing ethoxy end groups or ε-CL units were not observed. However, it is likely that formation of cyclic species *via* backbiting events would eliminate such end groups as low-molecular weight species not detectable by MALDI-TOF-MS. Selectivity toward forming cyclic P3HB was increased at lower catalyst loadings and, in the case of **3a**, at lower temperature. **3a** was more selective toward formation of cyclic P3HB than **4a** or **4b**. When **3a** was applied to the ROP of β-BL at 25 °C greater selectivity was observed toward formation of cyclic P3HB.

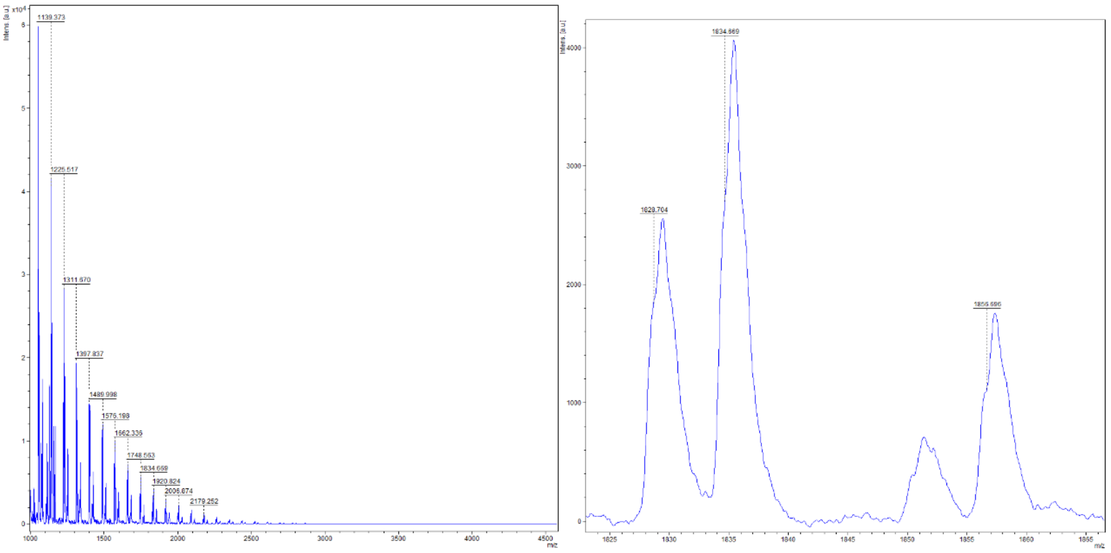

Figure S44. MALDI-TOF-MS spectrum of P3HB produced at 65 °C in the presence of 1 mol% of **4a**. Series, left, and detail, right.

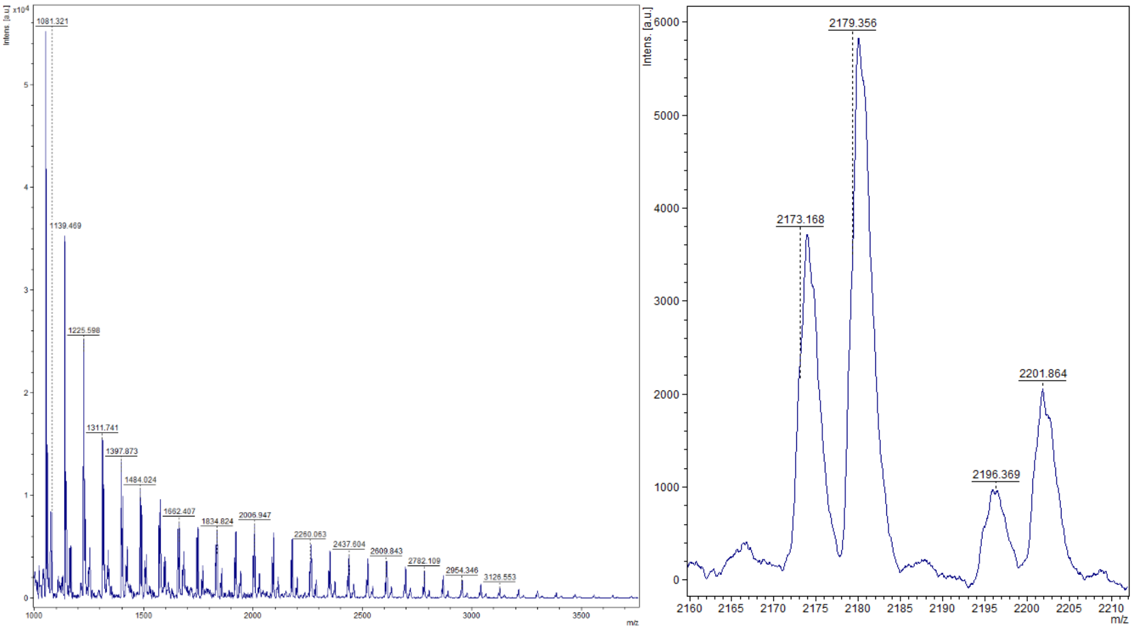

Figure S45. MALDI-TOF-MS spectrum of P3HB produced at 65 °C in the presence of 0.25 mol% of **4a**. Series, left, and detail, right.

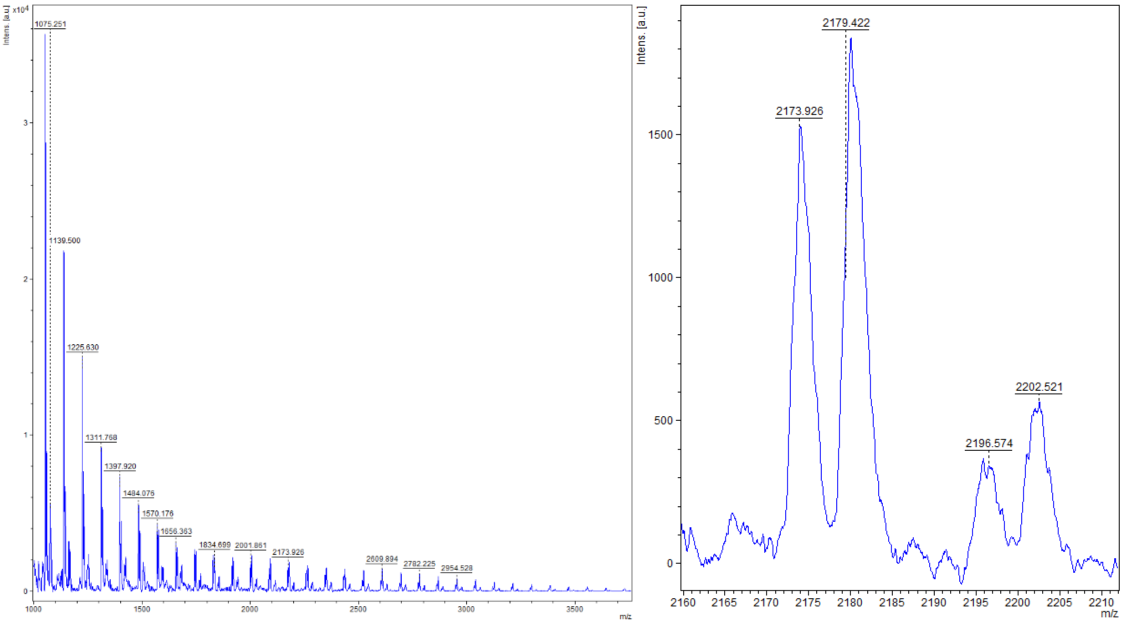

Figure S46. MALDI-TOF-MS spectrum of P3HB produced at 65 °C in the presence of 0.1 mol% of **4a**. Series, left, and detail, right.

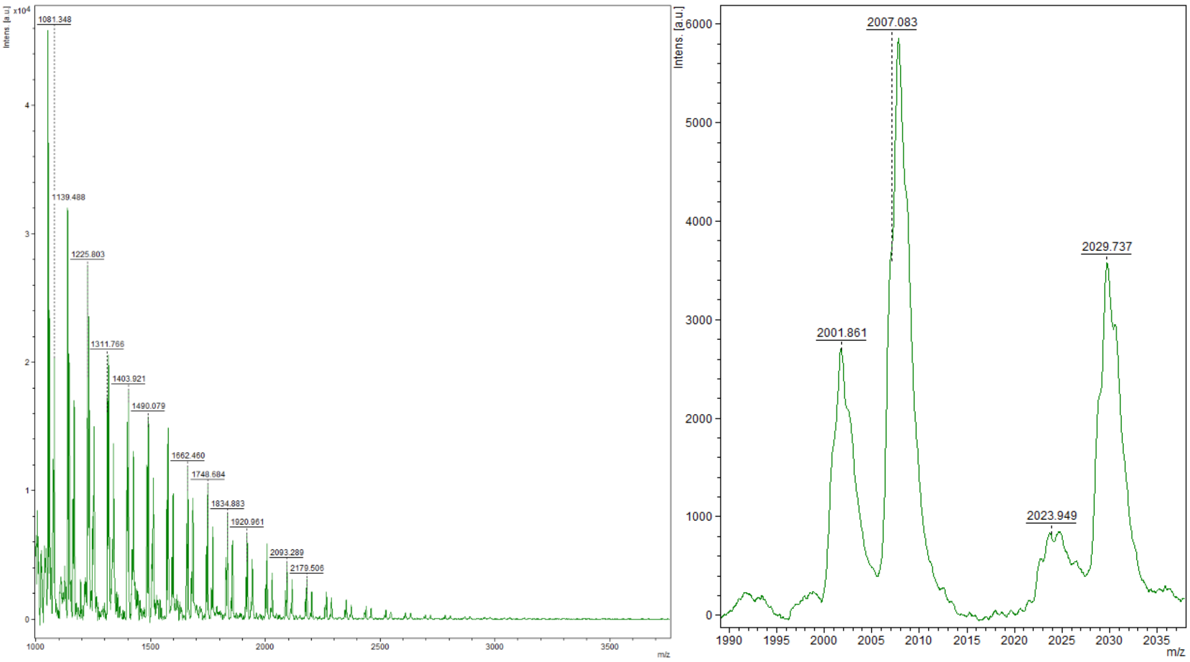

Figure S47. MALDI-TOF-MS spectrum of P3HB produced at 65 °C in the presence of 1 mol% of **4b**. Series, left, and detail, right.

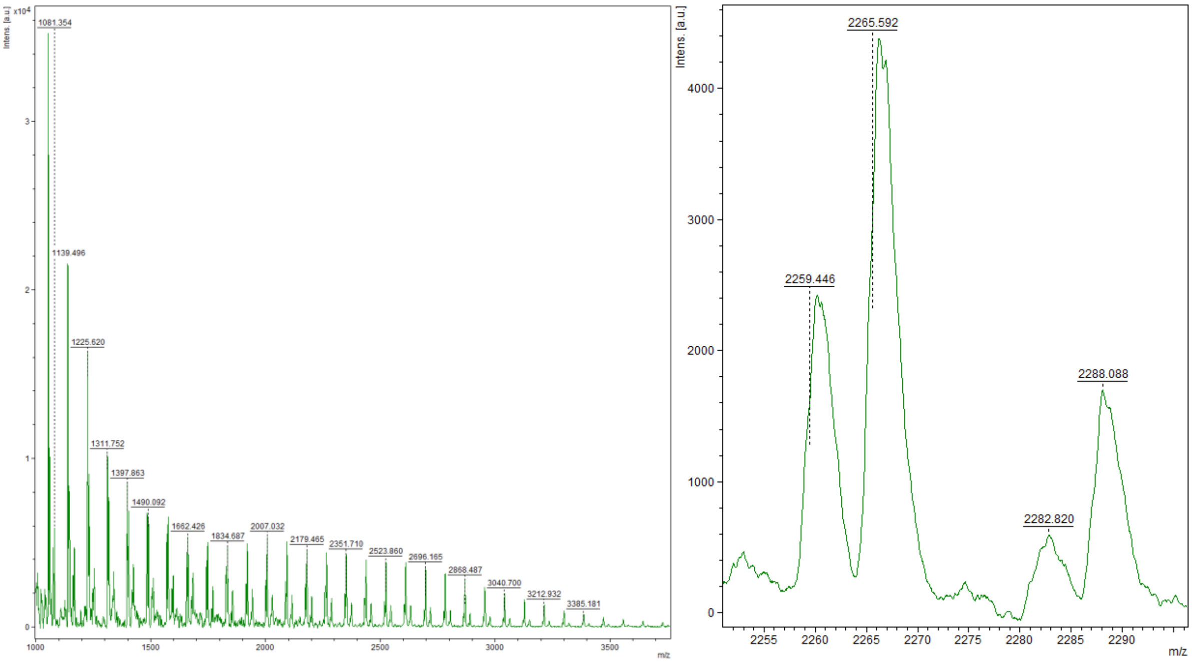

Figure S48. MALDI-TOF-MS spectrum of P3HB produced at 65 °C in the presence of 0.25 mol% of **4b**. Series, left, and detail, right.

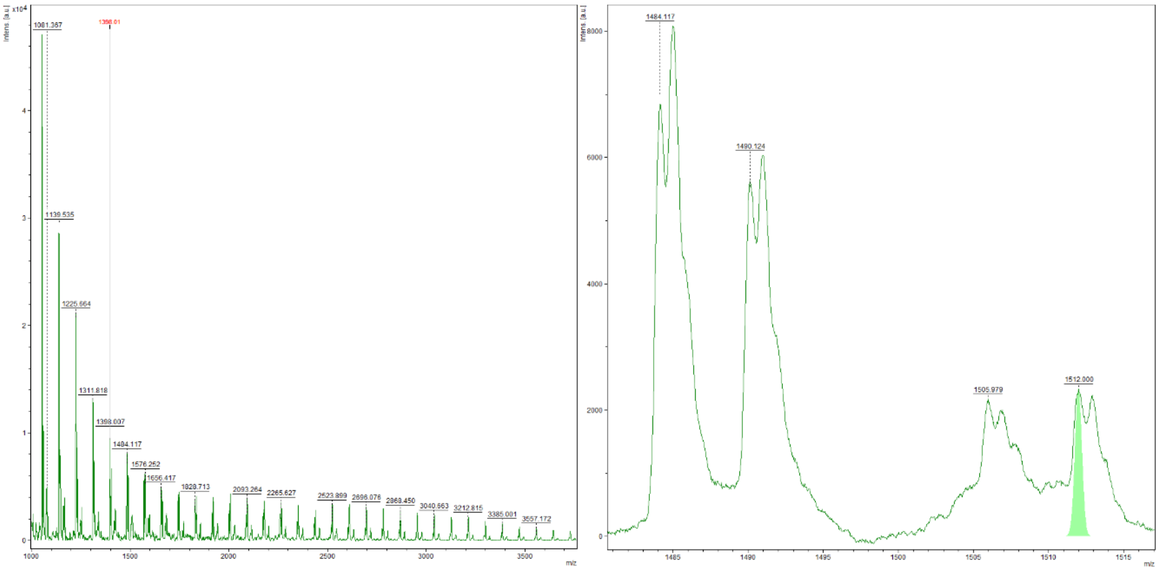

Figure S49. MALDI-TOF-MS spectrum of P3HB produced at 65 °C in the presence of 0.1 mol% of **4b**. Series, left, and detail, right.

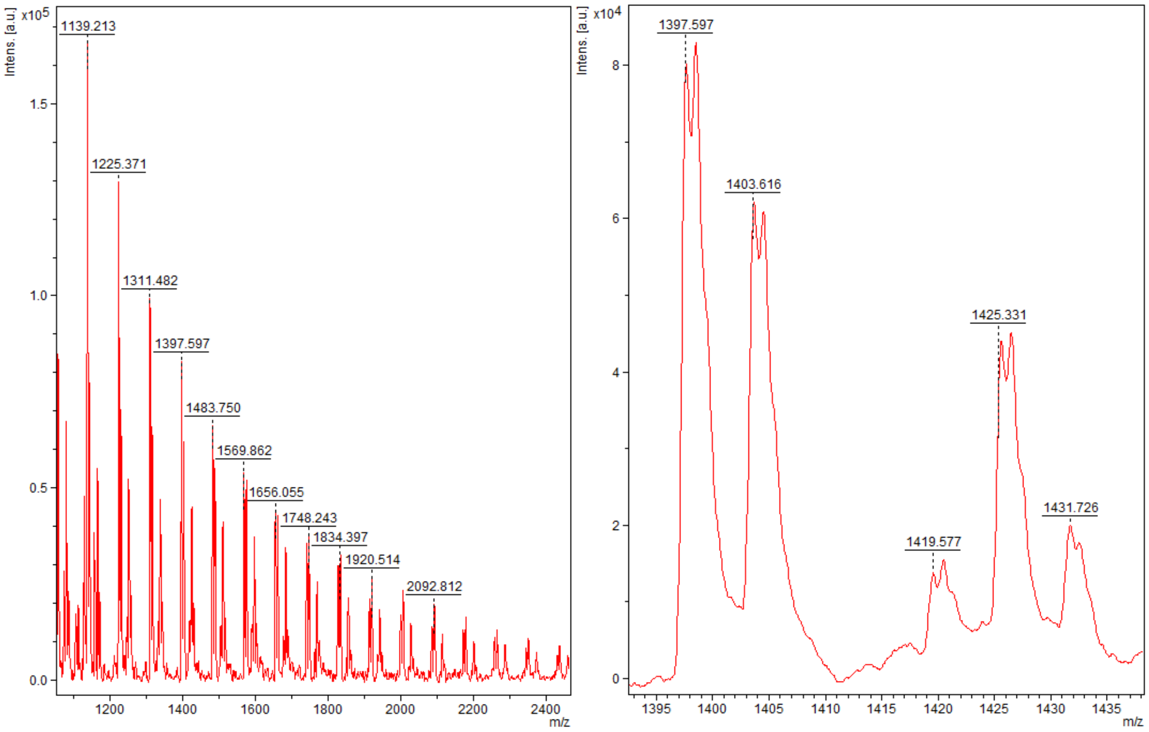

Figure S50. MALDI-TOF-MS spectrum of P3HB produced at 65 °C in the presence of 1 mol% of **3a**. Series, left, and detail, right.

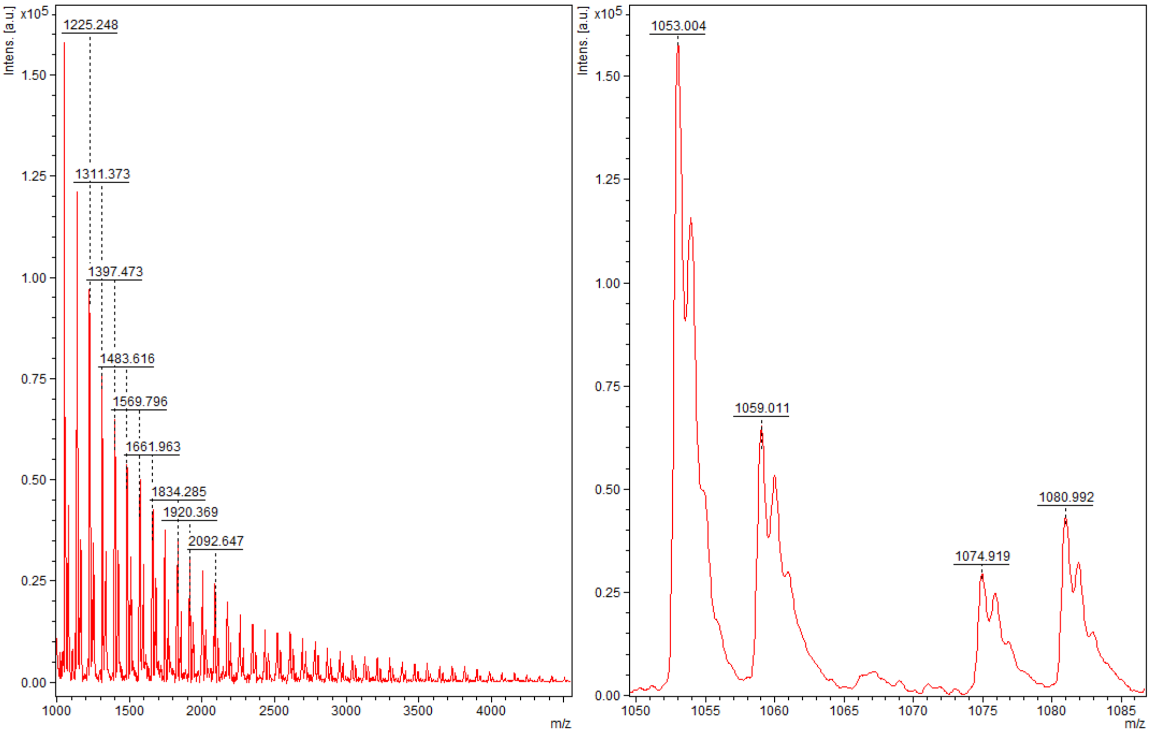

Figure S51. MALDI-TOF-MS spectrum of P3HB produced at 65 °C in the presence of 0.25 mol% of **3a**. Series, left, and detail, right.

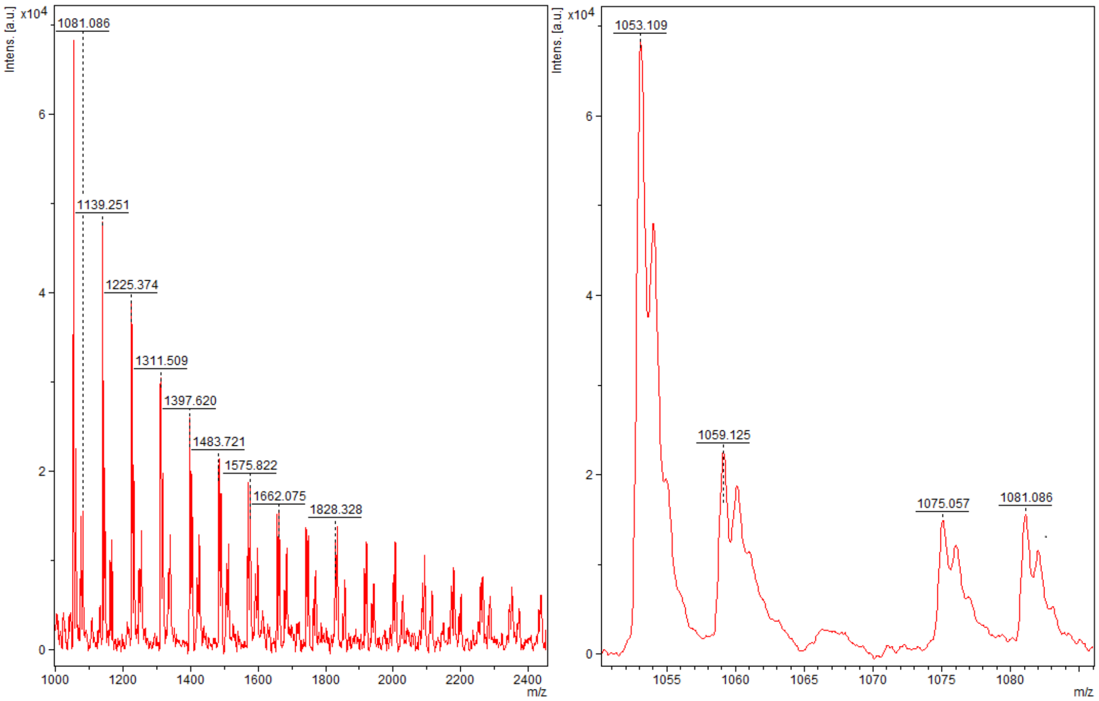

Figure S52. MALDI-TOF-MS spectrum of P3HB produced at 65 °C in the presence of 0.1 mol% of **3a**. Series, left, and detail, right.

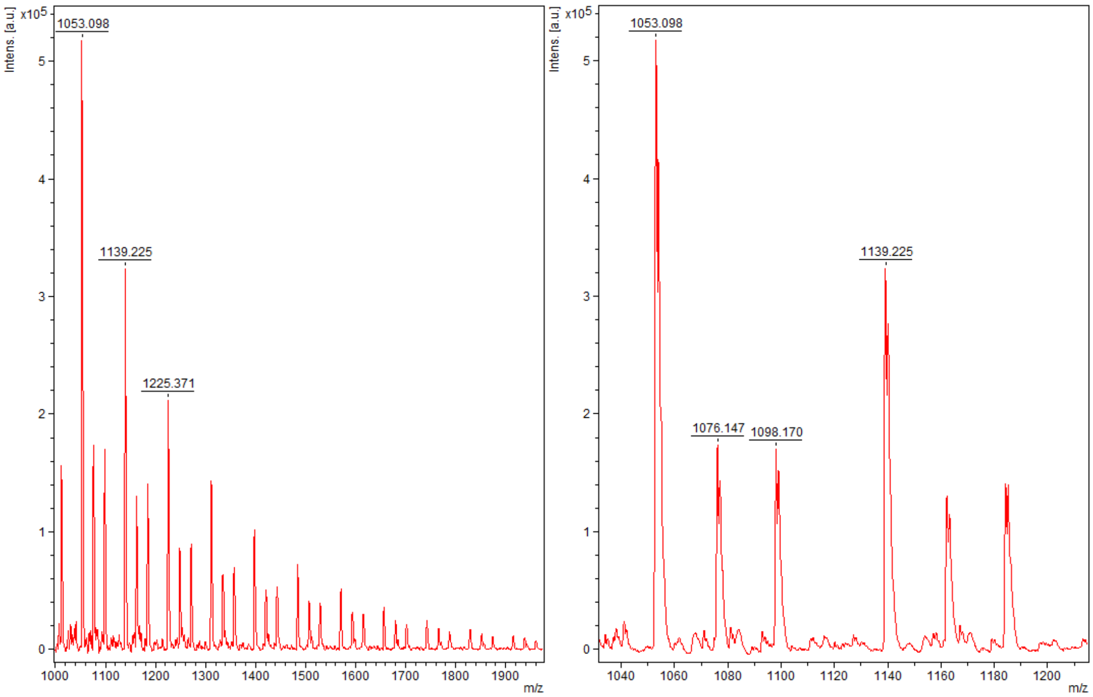

Figure S53. MALDI-TOF-MS spectrum of P3HB produced at 25 °C in the presence of 1 mol% of **3a**. Series, left, and detail, right.

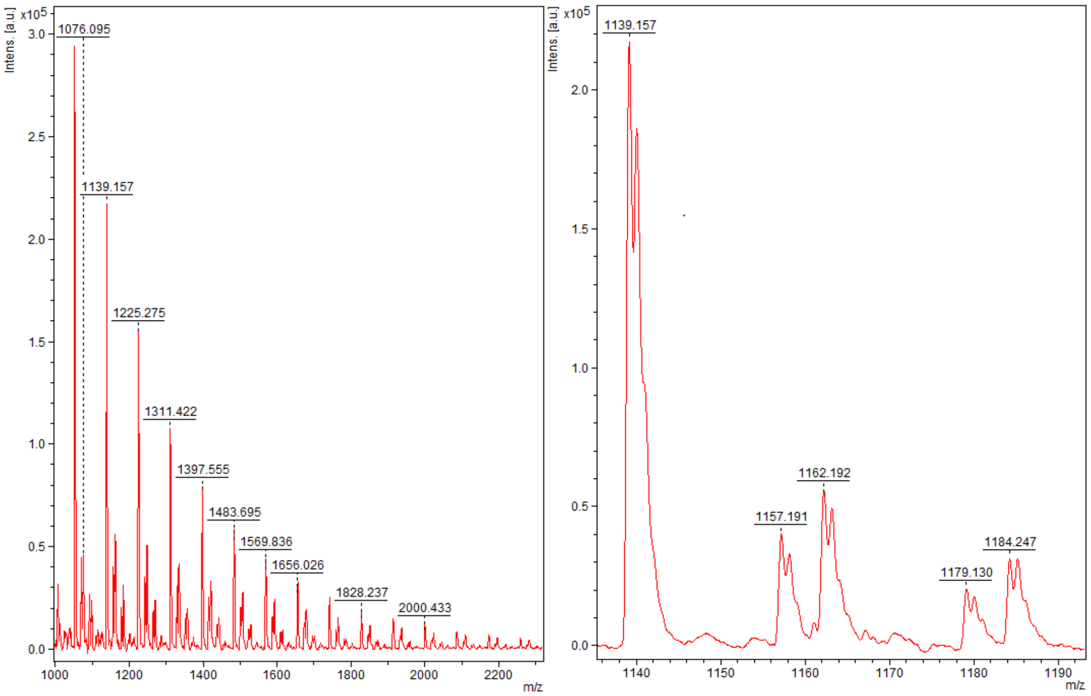

Figure S54. MALDI-TOF-MS spectrum of P3HB produced at 25 °C in the presence of 0.25 mol% of **3a**. Series, left, and detail, right.

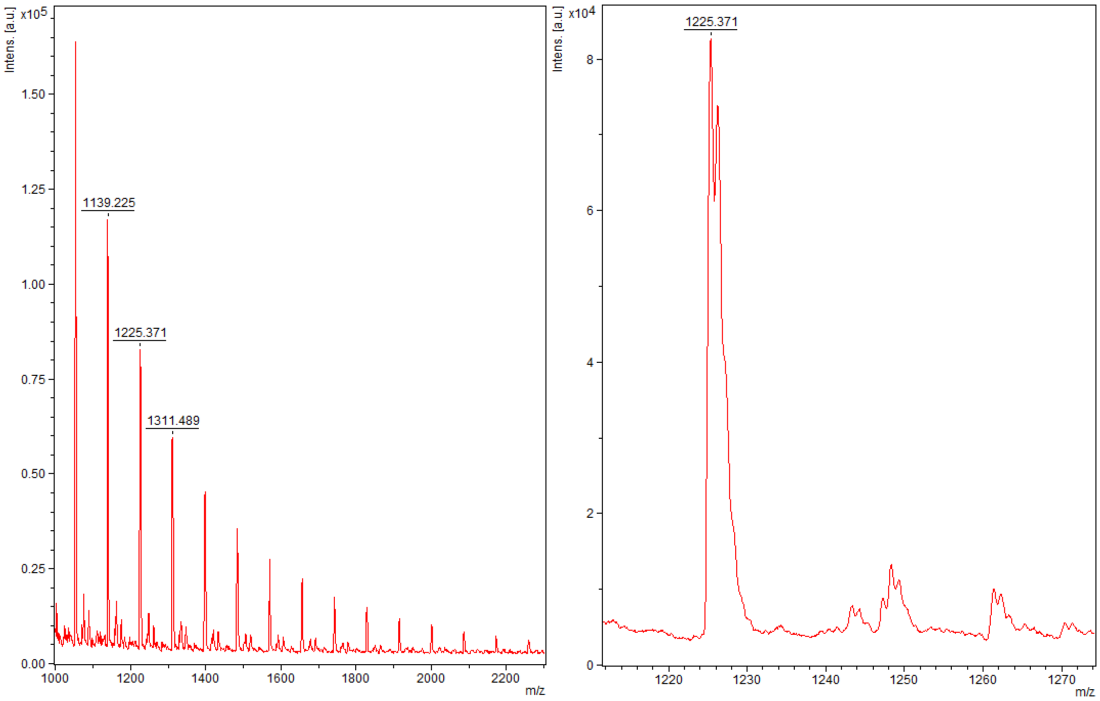

Figure S55. MALDI-TOF-MS spectrum of P3HB produced at 25 °C in the presence of 0.1 mol% of **3a**. Series, left, and detail, right.

Example GPC Traces

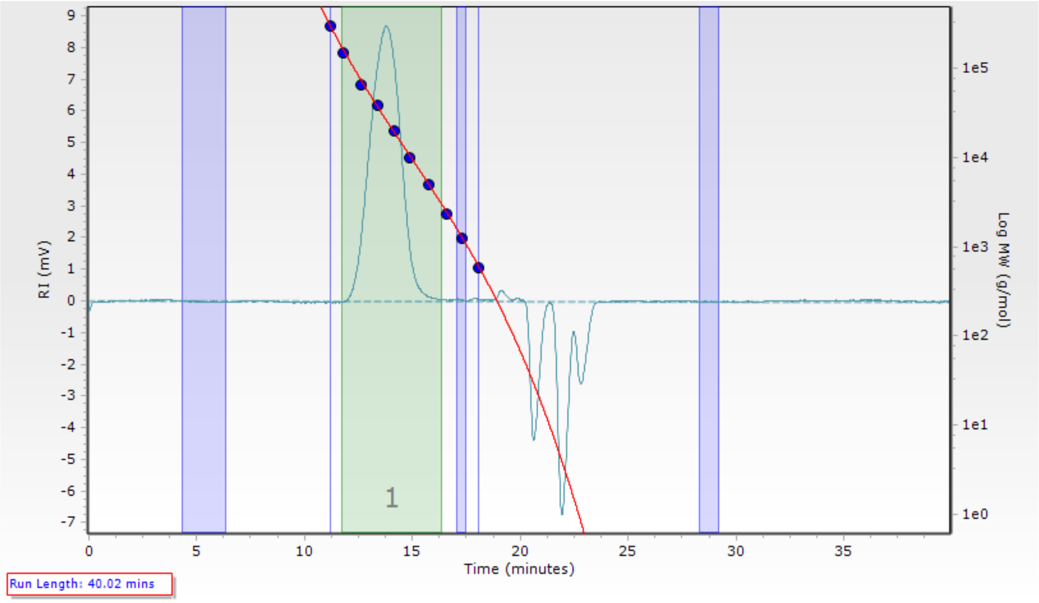

Figure S56. GPC trace for the product of ε-caprolactone polymerization at 80 °C in toluene-d_8, in the presence of 1 mol% of **4b**.

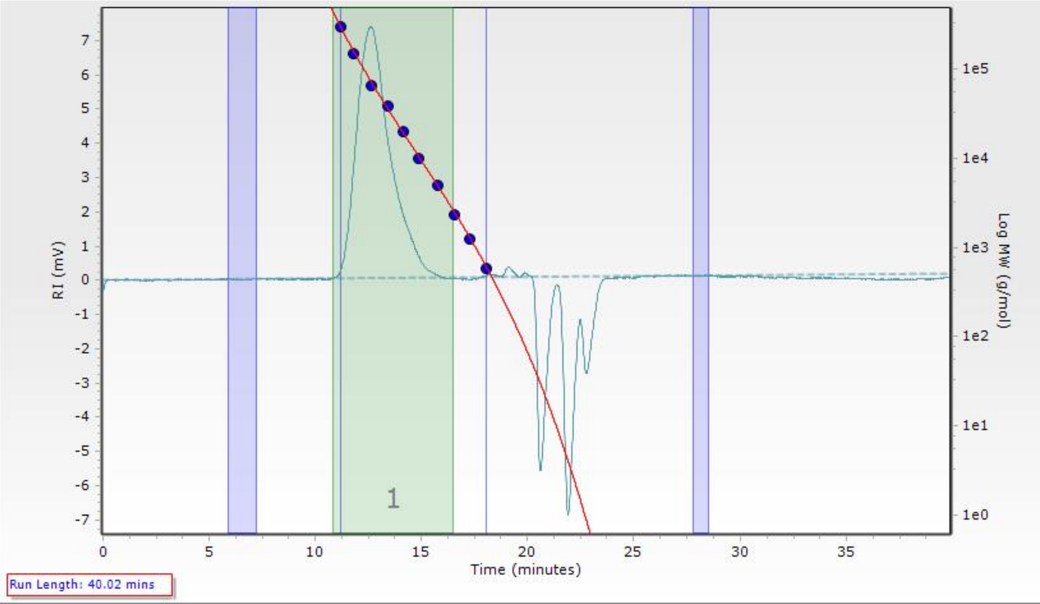

Figure S57. GPC trace for the product of ε-caprolactone polymerization at 80 °C in toluene, in the presence of 0.4 mol% of **4b**.

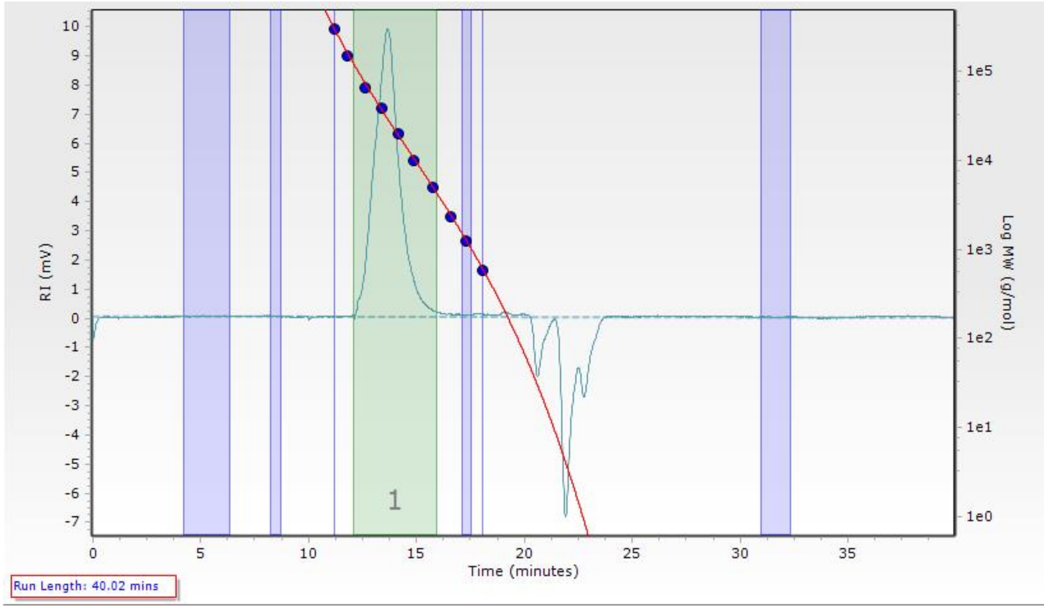

Figure S58. GPC trace for the product of ε-caprolactone polymerization at 80 °C in toluene, in the presence of 0.4 mol% of **4b** and 0.6 mol% of BnOH.

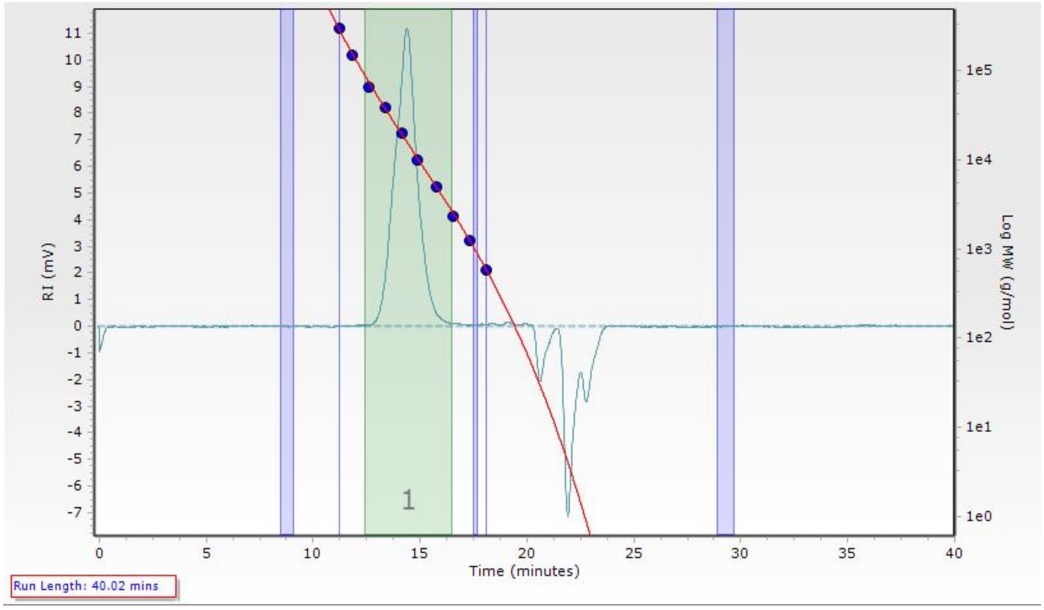

Figure S59. GPC trace for the product of ε-caprolactone polymerization at 80 °C in toluene, in the presence of 0.4 mol% of **4b** and 1.6 mol% of BnOH

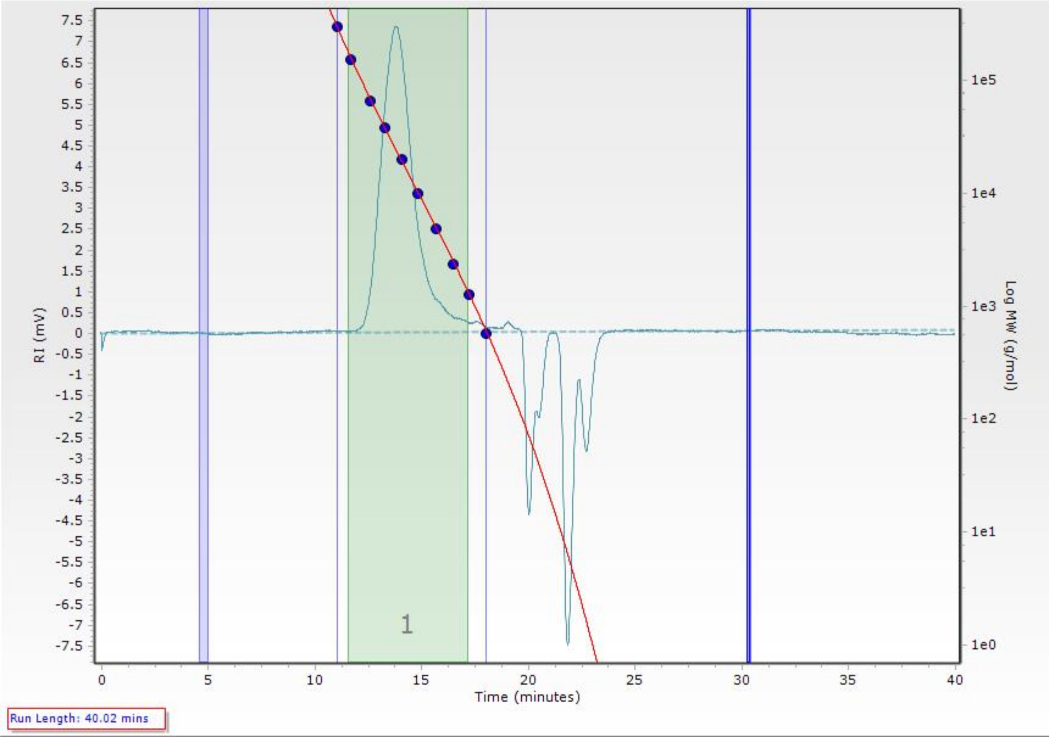

Figure S60. GPC trace for the product of δ-valerolactone polymerization at 80 °C in toluene, in the presence of 1 mol% of **4a**.

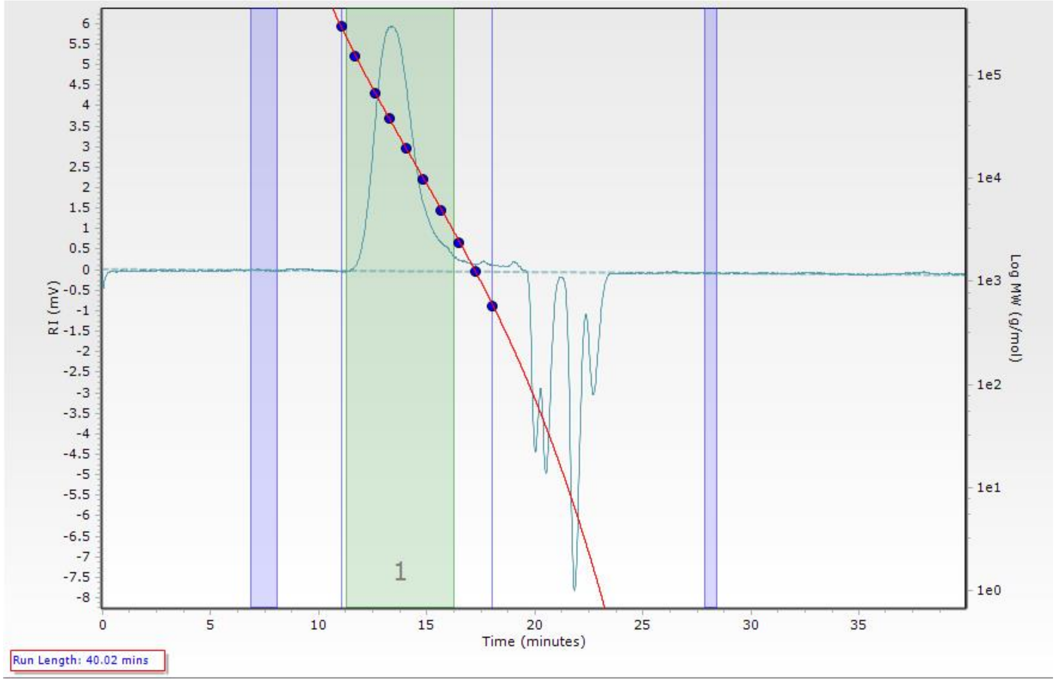

Figure S61. GPC trace for the product of δ-valerolactone polymerization at 80 °C in toluene, in the presence of 0.4 mol% of **4b**.

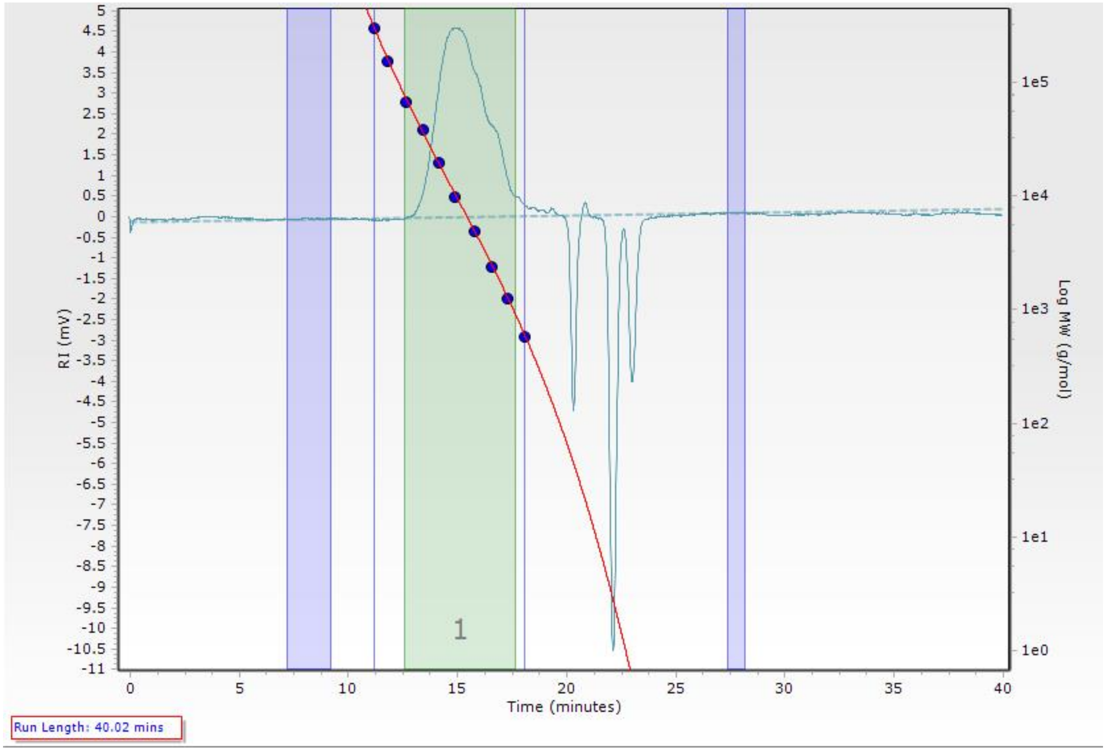

Figure S62. GPC trace for the product of δ-valerolactone polymerization at 80 °C in toluene, in the presence of 0.2 mol% of **4b**.

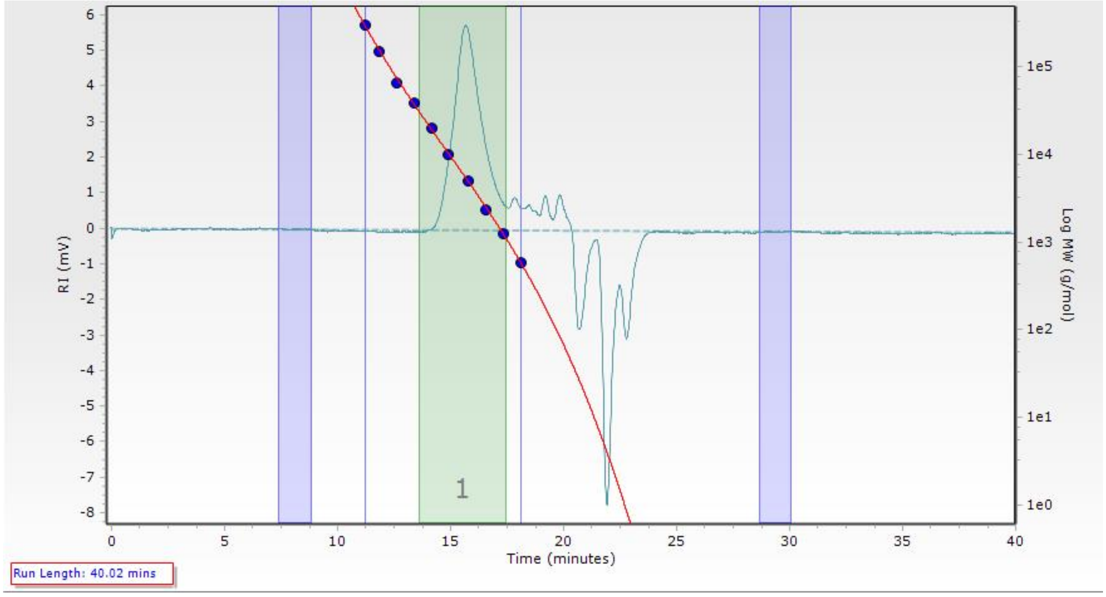

Figure S63. GPC trace for the product of *rac*-β-butyrolactone polymerization at 65 °C in toluene, in the presence of 0.1 mol% of **4b**.

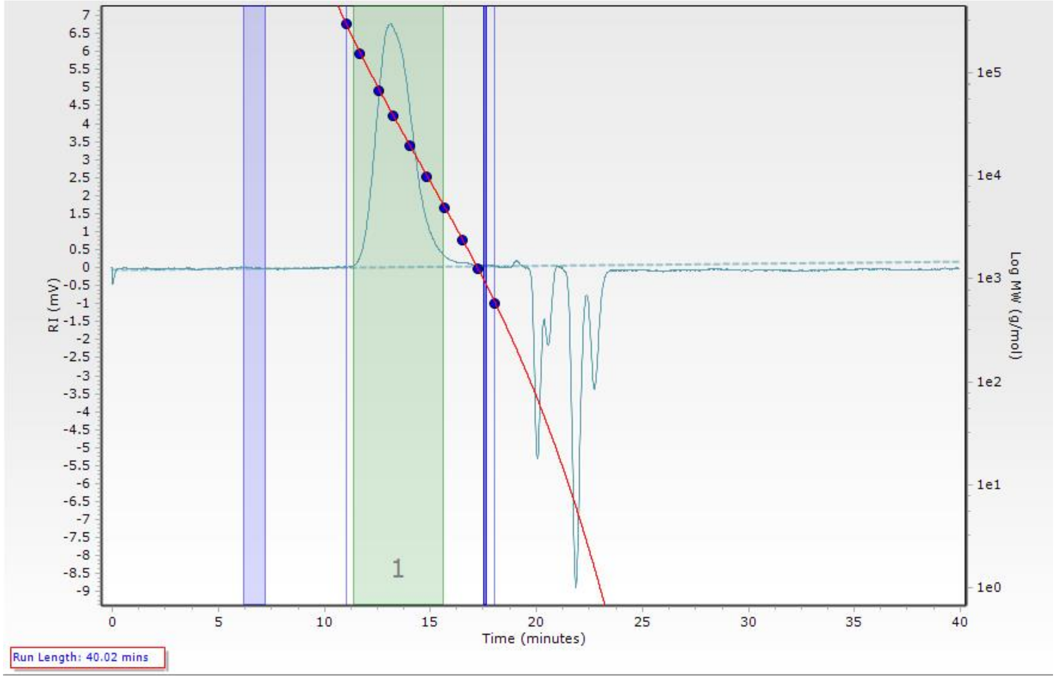

Figure S64. GPC trace for the product of ε-caprolactone polymerization at 80 °C in toluene, in the presence of 1 mol% of **5a**.

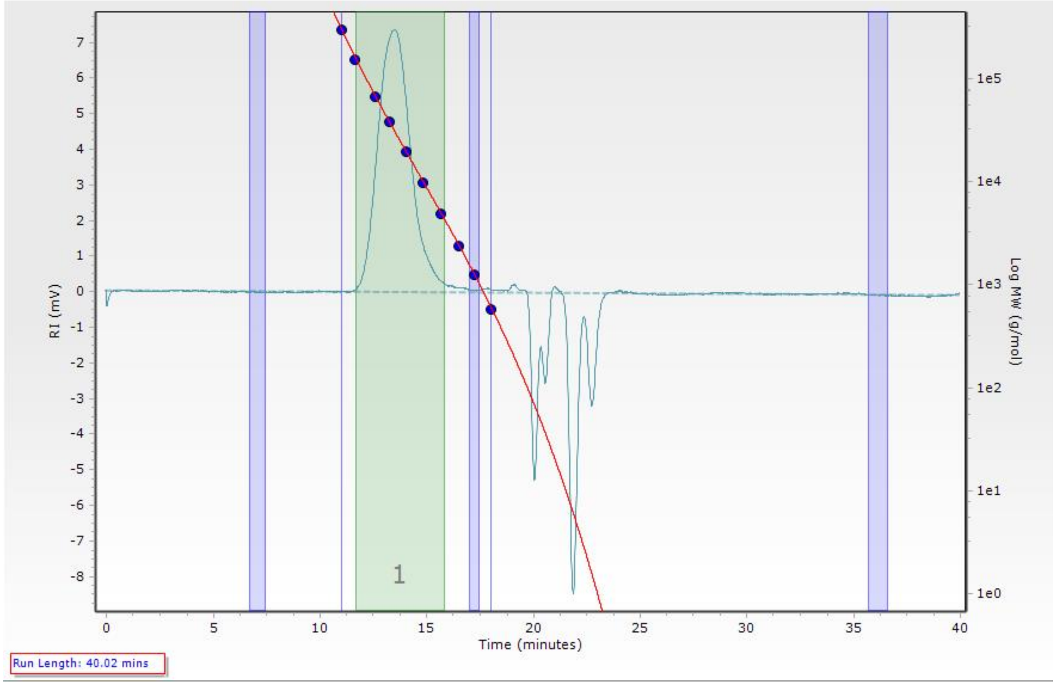

Figure S65. GPC trace for the product of ε-caprolactone polymerization at 80 °C in toluene, in the presence of 1 mol% of **5b**.

Crystallographic Parameters

Solid state structures for complexes **1b**, **2b**, **5a**, **4b** and **5b** are represented by CCDC deposition numbers 2290156-2290160.

Table S4. Crystallographic parameters for complexes **1b**, **2b**, **5a**, **4b** and **5b**

Compound reference	1b	2b	5a	4b	5b
Chemical formula	$8(C_{49}H_{76}NO_5Ta)\bullet C_{12}H_{12}$	$C_{47}H_{71}ClNO_4Ta$	$C_{52}H_{79}F_6NNbO_6$ Sb	$C_{67}H_{97}F_6NO_6$ SbTa	$C_{52}H_{79}F_6NO_6$ SbTa
Formula Mass	7676.64	930.44	1142.82	1429.15	1230.86
Crystal system	Tetragonal	Monoclinic	Monoclinic	Monoclinic	Monoclinic
a/Å	31.7034(2)	11.05400(10)	12.37780(10)	13.4337(3)	12.3899(5)
b/Å	31.7034(2)	20.8837(2)	14.41100(10)	27.1135(6)	14.419(3)
c/Å	9.85760(10)	20.3418(2)	31.5961(3)	19.0466(4)	31.554(3)
α/°	90	90	90	90	90
β/°	90	94.0040(10)	90.0800(10)	105.882(2)	90.139(6)
γ/°	90	90	90	90	90
Unit cell volume/Å^3	9907.93(16)	4684.41(8)	5636.00(8)	6672.6(3)	5637.1(13)
Temperature/K	150(2)	150(2)	150(2)	150(2)	150(2)
Space group	$P4/n$	$P2_1/n$	$P2_1/n$	$P2_1/n$	$P2_1/n$
No. of formula units per unit cell, Z	1	4	4	4	4
Radiation type	CuKα	CuKα	CuKα	MoKα	MoKα
No. of reflections measured	39264	14305	24287	54822	32788
No. of independent reflections	9882	14305	10664	13610	9529
R_{int}	0.0308	-	0.0253	0.0643	0.0516
Final R_1 values ($I > 2\sigma(I)$)	0.0302	0.0442	0.0438	0.0501	0.0448
Final $wR(F^2)$ values ($I > 2\sigma(I)$)	0.0686	0.1072	0.1150	0.1028	0.0615
Final R_1 values (all data)	0.0326	0.0541	0.0485	0.0852	0.0687
Final $wR(F^2)$ values (all data)	0.0698	0.1105	0.1177	0.1177	0.0672

References

1 M. Kol, M. Shamis, I. Goldberg, Z. Goldschmidt, S. Alfi and E. Hayut-Salant, *Inorg. Chem. Commun.*, 2001, **4**, 177–179.

2 T. R. Dargaville, P. J. De Bruyn, A. S. C. Lim, M. G. Looney, A. C. Potter, D. H. Solomon and X. Zhang, *J Polym Sci A Polym Chem*, 1997, **35**, 1389–1398.

3 A. Buchard, M. G. Davidson, G. Gobius du Sart, M. D. Jones, G. Kociok-Köhn, S. N. McCormick and P. Mckeown, *Inorg. Chem.*, 2023, **62**, 15688–15699.

4 A. Buchard, C. J. Chuck, M. G. Davidson, G. Gobius du Sart, M. D. Jones, S. N. McCormick and A. D. Russell, *ACS Catal.*, 2023, 2681–2695.

5 S. Groysman, S. Segal, I. Goldberg, M. Kol and Z. Goldschmidt, *Inorg. Chem. Commun.*, 2004, **7**, 938–941.

6 G. Gontard, A. Amgoune and D. Bourissou, *J. Polym. Sci. Part A Polym. Chem.*, 2016, **54**, 3253–3256.

7 M. Save, M. Schappacher and A. Soum, *Macromol. Chem. Phys.*, 2002, **203**, 889–899.

8 A. Amgoune, C. M. Thomas, S. Ilinca, T. Roisnel and J. F. Carpentier, *Angew. Chemie - Int. Ed.*, 2006, **45**, 2782–2784.
